# The Efficacy of Ginseng (Panax) on Human Prediabetes and Type 2 Diabetes Mellitus: A Systematic Review and Meta-Analysis

**DOI:** 10.3390/nu14122401

**Published:** 2022-06-09

**Authors:** Kaveh Naseri, Saeede Saadati, Amir Sadeghi, Omid Asbaghi, Fatemeh Ghaemi, Fatemeh Zafarani, Hua-Bin Li, Ren-You Gan

**Affiliations:** 1 Research Center for Plants and Human Health, Institute of Urban Agriculture, Chengdu National Agricultural Science & Technology Center, Chinese Academy of Agricultural Sciences, Chengdu 610213, China; naserikaveh1@gmail.com; 2 Department of Medicine, School of Clinical Sciences, Monash University, Melbourne, VIC 3168, Australia; saadatisaeede@gmail.com; 3 Gastroenterology and Liver Diseases Research Center, Research Institute for Gastroenterology and Liver Diseases, Shahid Beheshti University of Medical Sciences, Tehran 1985717413, Iran; amirsadeghimd@yahoo.com; 4 Cancer Research Center, Shahid Beheshti University of Medical Sciences, Tehran 1983963113, Iran; omid.asbaghi@gmail.com; 5 Department of Transplantation and Disease, Vice Chancellary for Treatment, Iran Ministry of Health and Medical Education, Tehran 1419943471, Iran; ghaemifa77@gmail.com; 6 Student Research Committee, Tabriz University of Medical Sciences, Tabriz 5165665931, Iran; haniehzfr@gmail.com; 7 Guangdong Provincial Key Laboratory of Food, Nutrition and Health, Department of Nutrition, School of Public Health, Sun Yat-Sen University, Guangzhou 510080, China; lihuabin@mail.sysu.edu.cn; 8 Key Laboratory of Coarse Cereal Processing (Ministry of Agriculture and Rural Affairs), Sichuan Engineering & Technology Research Center of Coarse Cereal Industrialization, School of Food and Biological Engineering, Chengdu University, Chengdu 610106, China

**Keywords:** Panax, ginseng, blood lipid, inflammation, cardiometabolic indicators, prediabetes, diabetes mellitus

## Abstract

Results from different clinical trials on the effects of ginseng on prediabetes and type 2 diabetes (T2DM) are still inconsistent. To fill this knowledge gap, we investigated the overall effects of ginseng supplementation on improving cardiometabolic biomarkers among these patients. A systematic literature search was conducted on PubMed/MEDLINE, Scopus, Web of Science, and Cochrane library. A random-effect model was applied to estimate the weighted mean difference and 95% CI for each outcome. Overall, 20 eligible RCTs were included. Meta-analyses revealed that ginseng supplementation significantly reduced serum concentration of FPG, TC, IL-6, and HOMA-IR values. It also increased HR and TNF-α levels. Ginseng supplementation changed HOMA-IR and HDL-C significantly based on dose and changed HOMA-IR and LDL-C significantly based on study duration in a non-linear fashion. Furthermore, meta-regression analyses indicated a linear relationship between ginseng dose and absolute changes in HDL-C. Moreover, subgroup analyses showed that ginseng supplementation changed TC and LDL-C when the supplementation dose was ≥2 g/day. Our findings suggest that ginseng supplementation may be an effective strategy for improving cardiometabolic profiles in individuals with prediabetes and T2DM.

## 1. Introduction

Type 2 diabetes mellitus (T2DM) is the fastest-growing metabolic disorder worldwide, imposing social, economic, and public health burdens [1,2,3]. One major diabetes comorbidity is cardiovascular disease (CVD), accounting for 32.2% of all individuals with diabetes [3]. These dysglycemia conditions are characterized by insulin resistance and β-cell dysfunction in adults [4]. Despite an increased emphasis on preemptive therapeutic options and promising new therapies, the management of T2DM remains challenging [5,6]. Meanwhile, interest in complementary and alternative medicine (CAM) remarkably continues to increase [7], becoming one of the major therapeutic approaches sought by individuals with diabetes [8].

Many herbal medications have been recommended for controlling diabetes [9]. Among them, the most popular one is ginseng, the root of plants in the Panax genus of the Araliaceae family, whose effects on complications of T2DM have been investigated extensively [10,11,12,13,14]. Panax ginseng (commonly known as Asian ginseng) and Panax quinquefolius (commonly known as American ginseng) are the most widely commercialized types [15]. Ginseng has been shown to exert various biological impacts, including anti-diabetic [6], anti-hyperlipidemic [16], anti-inflammatory [17], and hepatoprotective [18] effects. Several pathways have been proposed in this context. Major ones include reducing leptin and neuropeptide Y concentrations and appetite suppression [19], influencing inflammatory signaling pathways such as nuclear factor-kappa β (NF-κB) and activator protein 1 (AP-1) [15] and suppressing Peroxisome proliferator-activated receptor α (PPARα) [20]. 

Many clinical studies have investigated the potential protective effects of ginseng on cardiometabolic indices among individuals with diabetes; however, the results have been inconsistent [1,5,21,22,23,24,25]. Some studies documented improved levels of anthropometric indices [1], glucose-related markers [26,27], lipid profile components [5], blood pressure [28], and inflammatory markers [21] following ginseng consumption, whereas others indicated null effects [12,22,29,30]. Furthermore, previous systematic reviews and meta-analyses of randomized controlled trials (RCTs) on ginseng supplementation have only covered certain cardiometabolic indicators in various health conditions [19,31,32,33,34,35,36]. Besides, there was no prior systematic review and meta-analysis investigating the effects of ginseng supplementation on cardiometabolic indicators exclusively in individuals with prediabetes and T2DM. Thus, the objective of this study was to comprehensively summarize and analyze the evidence from clinical trials relating to the impact of ginseng supplements on various cardiometabolic outcomes among subjects with dysglycemia.

## 2. Materials and Methods

We performed and reported this systematic review and meta-analysis in accordance with the Preferred Reporting Items for Systematic Review and Meta-analysis (PRISMA) checklist [37]. 

### 2.1. Data Sources and Searches

Studies were systematically identified using electronic databases (PubMed/MEDLINE, Scopus, Web of Science, and Cochrane library) until 10 April 2022. The following keywords were used in the search strategy: ((Ginseng OR ginsenoside) AND (Intervention OR “controlled trial” OR randomized OR random OR randomly OR placebo OR “clinical trial” OR Trial OR “randomized clinical trial” OR RCT OR trial OR trials “Cross-Over Studies” OR “Cross-Over” OR “Cross-Over Study” OR parallel OR “parallel study” OR “parallel trial”) AND (“diabetes” OR “type 2 diabetes mellitus” OR “T2DM” OR “type 2 diabetes” OR “T2D” OR “prediabetes”)). Search terms used across the various databases are presented in Supplementary Table S1. No date restrictions were applied; however, only English-language articles were eligible for inclusion. Bibliographies of relevant studies or systematic reviews identified by the search strategy were screened for additional studies. If the data required for the meta-analysis was not reported in the literature, we contacted the corresponding author to provide the data. 

### 2.2. Study Selection and Eligibility Criteria

All recorded articles from electronic or manual searches were imported into Endnote software for further review. Titles and abstracts of all articles found in the initial search were reviewed independently by two researchers (S.S. and K.N.). Discussion with a third reviewer (O.A.) resolved disagreement regarding full-text eligibility. To determine article eligibility, we employed the population, intervention, comparison, outcome, and study design (PICOS) framework (Table 1). Studies were excluded from this investigation if they: (1) co-administered ginseng as a part of a mixed intervention; (2) lacked a suitable control; (3) had no viable end-point data in ginseng or control groups; (4) were carried out on children, pregnant women, or animals; and (5) were performed less than 4 weeks in duration. In addition, conference abstracts, gray literature, unpublished studies, and protocols were not included.

### 2.3. Data Extraction

The following information was extracted from each eligible clinical trial by two independent researchers: study author; year of publication; study location; study design; the number of participants; participants’ ethnicity, age, comorbidities, and body mass index; the type, dose, duration, and frequency of the intervention; and the study results (mean or median with standard deviations, standard errors, 95% CIs, or interquartile ranges) at study baseline, post-intervention, and/or changes between baseline and post-intervention. If the data for each parameter was reported in different units, we converted them to the most commonly used units.

### 2.4. Quality Assessment

The likelihood of bias in the included RCTs was explored through the Cochrane Risk of Bias Tool for clinical trials [38]. Two independent authors assessed each publication’s quality based on the following seven domains: (1) random sequence generation; (2) allocation concealment; (3) selective outcome reporting; (4) blinding of participants and personnel; (5) detection bias (blinding of evaluators); (6) incomplete outcome data; and (7) other probable sources of biases. Based on the Cochrane handbook recommendation, every article was assigned a label of bias (low risk (L), high risk (H), or unclear (U) risk of bias) (Supplementary Table S2). 

The quality of the evidence for each result was assessed using the Grading of Recommendations, Assessment, Development, and Evaluation (GRADE) approach [39]. Two independent reviewers (S.S. and K.N.) graded each outcome based on the risk of bias, inconsistency (heterogeneity), indirectness, and imprecision, as specified in the GRADE guideline [39]. Each outcome was rated high, moderate, low, or very low.

### 2.5. Data Synthesis and Meta-Analysis

We used the mean difference in the changes in the outcome variables (BW, BMI, WC, FPG, OGTT, HbA1c, fasting insulin, HOMA-IR, TG, TC, LDL-C, HDL-C, SBP, DBP, HR, CRP, IL-6, TNF-α, ALT, AST, and GGT), comparing ginseng to the control groups, to obtain the overall effect sizes. When mean changes were not reported, we computed them by considering changes in each outcome variable during the trial. We also converted standard errors (SEs), 95% confidence intervals (CIs), and interquartile ranges (IQRs) to SDs using the relevant formulas [40]. We applied a random-effects model that considers between-study variations to obtain the overall effect sizes. Heterogeneity was determined using the I^2^ statistic and the Cochrane’s Q test. The Q-test’s I^2^ value > 50% or *p* < 0.05 was characterized as significant between-study heterogeneity [41,42]. Subgroup analyses were performed to find probable sources of heterogeneity, according to the predefined criteria, including ginseng dosage (≥2/<2 g/day), length of follow-up (>8/≤ 8 weeks), baseline levels of outcome variables (abnormal/normal levels) and participants’ baseline BMI level (normal/overweight or obese). Fractional polynomial modeling was executed to determine the potential non-linear effects of ginseng dosage (g/day) on each index. Due to the lack of information on ginseng dosage in some of the included studies, we decided to perform a non-linear dose-response analysis of ginseng administration. Sensitivity analysis was used to determine the extent to which inferences might depend on a particular study. The formal test of Begg assessed the possibility of publication bias. The meta-analysis was performed using the Stata version 11.2 (StataCorp). The *p*-value < 0.05 was considered statistically significant.

## 3. Results

### 3.1. Study Selection

**Figure 1:**
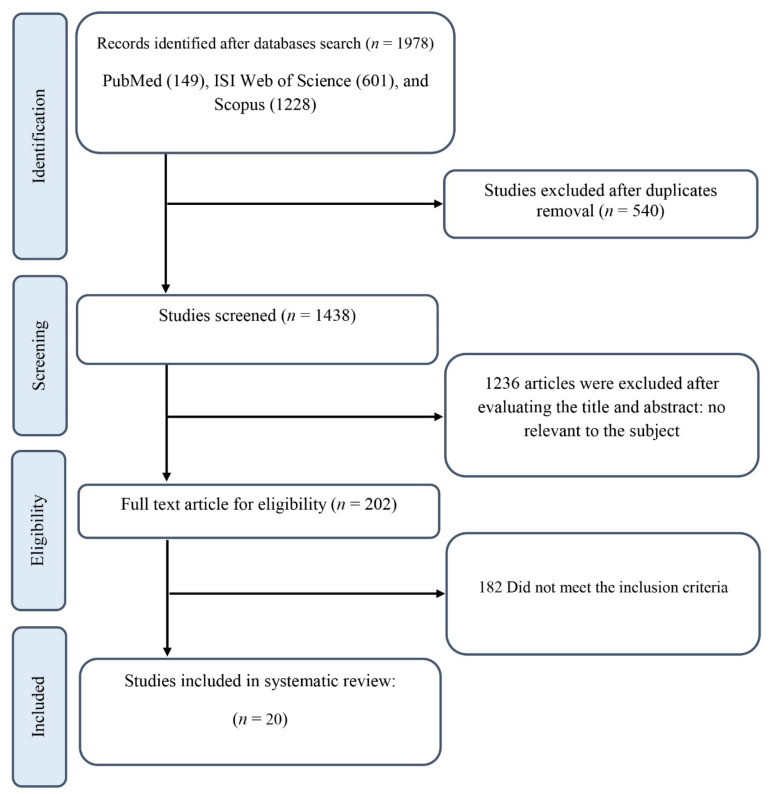
The Preferred Reporting Items for Systematic Review and Meta-analysis(PRISMA) flowchart.

Figure 1 illustrates the literature search and screening process performed for this systematic review. Initial database searches for all RCTs of ginseng supplementation yielded 1978 records, of which 540 were duplicates. Eligibility based on title and abstract was assessed for the remaining 1438 articles. Of those, 1236 RCTs were not relevant to the subject, leaving 202 records eligible for full-text review. Furthermore, after excluding 6 studies examining the impact of ginseng in combination with other ingredients due to the impossibility of determining the independent effect of ginseng [43,44,45,46,47,48], 3 eligible studies which reported in a language other than English [49,50,51], 1 study with multiple-crossover, acute dose escalation design [52], and 172 records which did not provide sufficient data and/or did not fulfill the inclusion criteria, 20 RCTs with 24 effect sizes met the inclusion criteria for qualitative synthesis.

### 3.2. Study Characteristics

The detailed characteristics of all the studies included in the qualitative synthesis are described in Table 2. The selected 20 studies were all RCTs, including 17 parallel design studies [1,21,22,23,24,25,26,27,28,29,30,53,54,55,56,57,58] and 3 crossover design studies [5,12,59]. In total, 1295 participants (675 cases and 620 controls) with age range between 44.56 ± 10.48 and 64 ± 7 years old and BMI range between 23.52 ± 0.48 and 36 ± 3.46 kg/m^2^ were recruited. These RCTs were conducted in South Korea [24,25,26,27,30,53,56], Iran [21,29,54], Canada [5,12], Croatia [23,28], Canada and Croatia [22,55], the United States [57], Australia [1], Hong Kong [59], and Finland [58], and were published between 1995 and 2021. RCTs were performed on individuals with prediabetes [1,27,56], and T2DM [5,12,21,22,23,24,25,26,28,29,30,53,54,55,57,58,59]. One study was exclusively performed on male subjects [57], and others were conducted on both genders. Of the 20 RCTs, 5 effect sizes administered Panax quinquefolius (also known as American ginseng) [5,22,23,28,55], and 13 used Panax ginseng (Korean ginseng, Asian ginseng, and Chinese ginseng) [1,12,21,22,24,25,26,27,30,53,54,55,56,57,59] as investigational products. The remaining two studies did not report the type of ginseng [29,58]. The dosage of ginseng varied from 0.1–8 g/day, and the duration of intervention differed from 4 to 24 weeks across included RCTs.

### 3.3. Effects of Ginseng Supplementation on Cardiometabolic Parameters

#### 3.3.1. Anthropometric Measurements

There was no significant difference between the ginseng and placebo groups at follow-up regarding BW (weighted mean difference (WMD):−0.54 kg; 95% CI: −2.54, 1.46; *p* = 0.598; phet = 0.998, I^2^ = 0.0%) [1,5,21,57], BMI (WMD: 0.05 kg/m2; 95% CI: −0.26, 0.38; *p* = 0.717; phet = 0.963, I^2^ = 0.0%) [1,21,24,25,30,57], and WC (WMD: 0.05 cm; 95% CI: −1.16, 1.27; *p* = 0.929; phet =0.729, I^2^ = 0.0%) (Supplementary Figure S1) [30]. It was impossible to conduct subgroup analyses for BW and WC as there were not enough studies (less than 2) in each group that reported on these measures. However, subgroup analyses did not reveal any differences in BMI irrespective of participants’ baseline BMI values, amount of supplemented ginseng, and length of follow-up (Table 3).

#### 3.3.2. Measures of Glucose Homeostasis 

##### Glycemic Control

**Figure 2:**
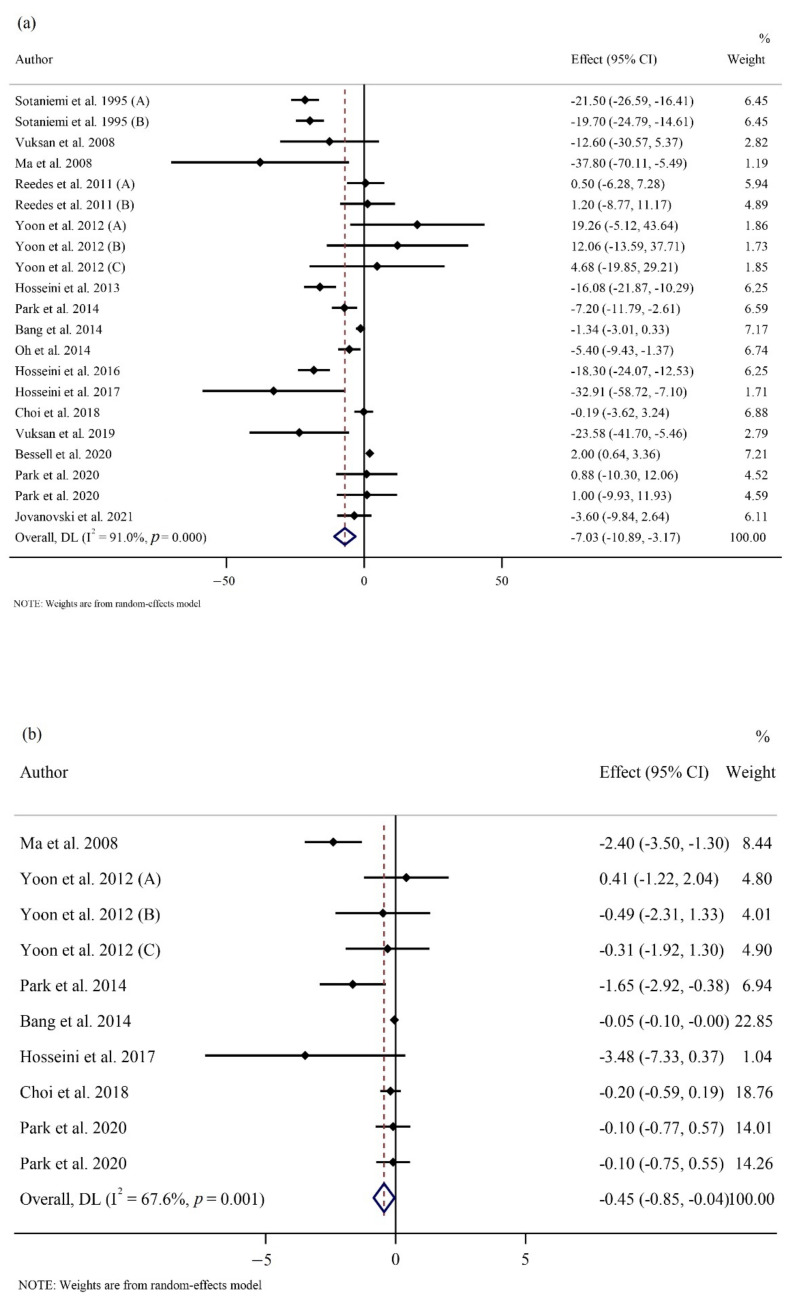

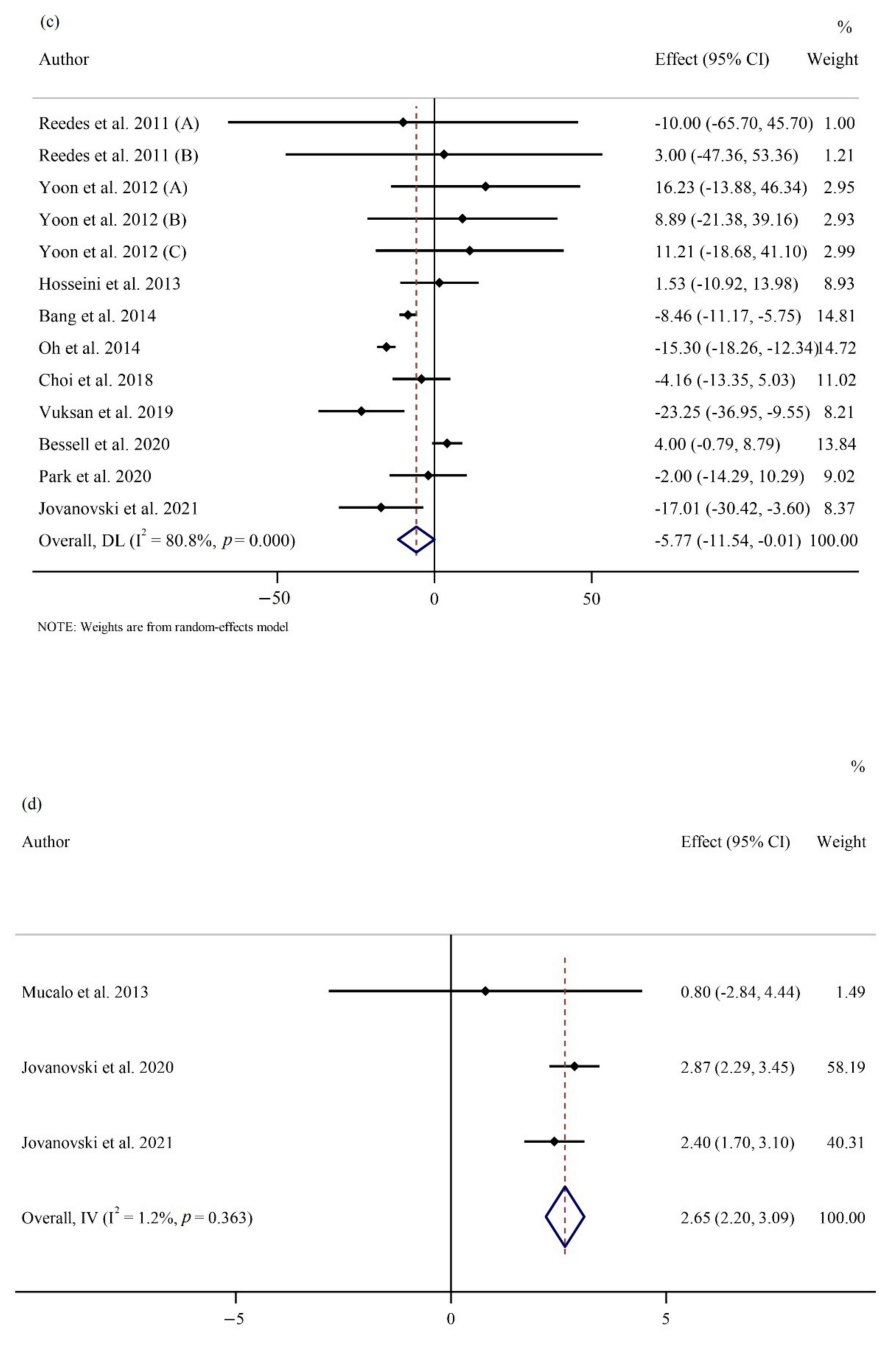

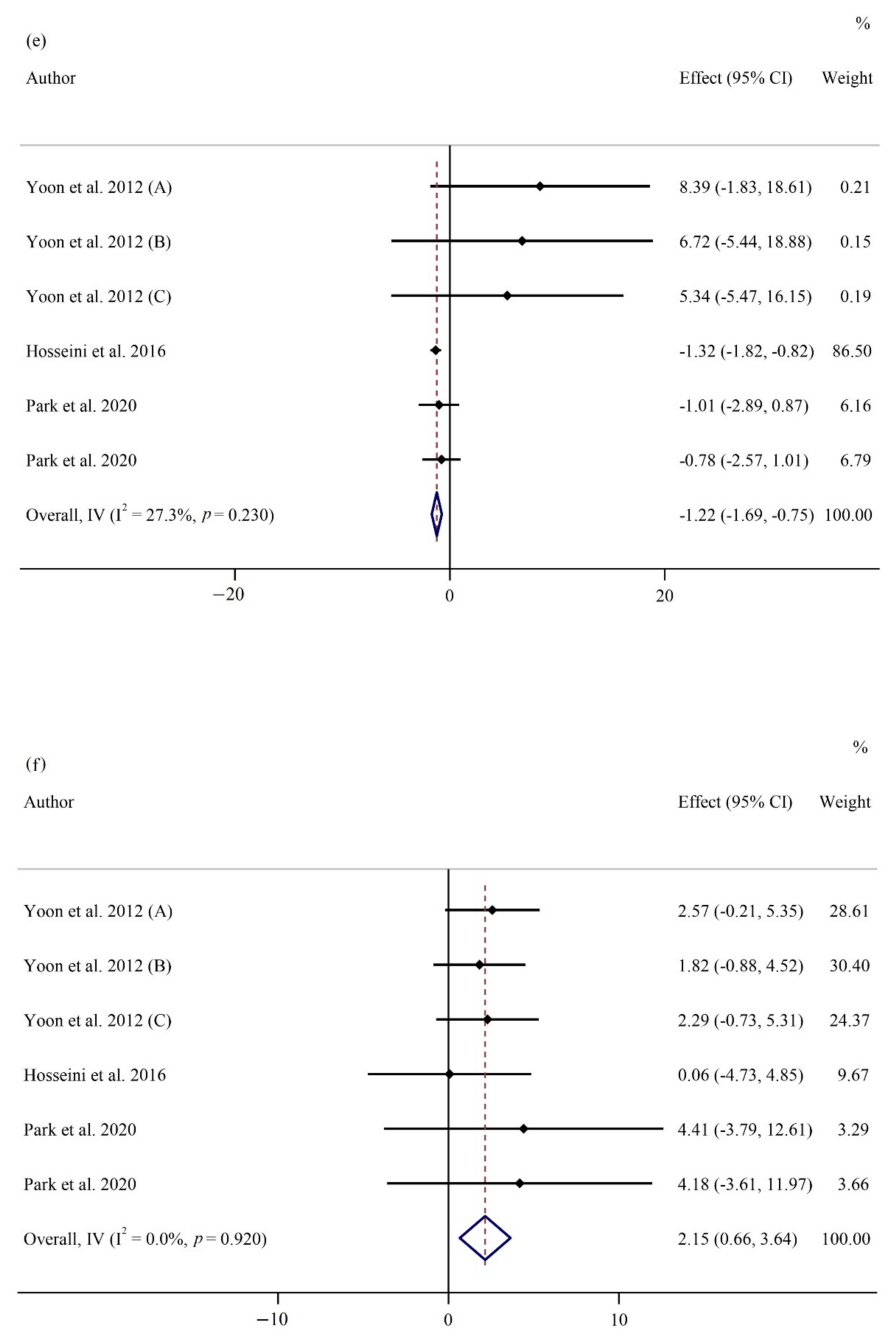
Forest plots of randomized controlled trials illustrating weighted mean difference (WMD) in biomarkers between the intervention and placebo groups for all eligible studies in overall analysis. (**a**) Fasting plasma glucose (FPG) [1,5,12,21,22,24,25,26,27,29,30,53,54,56,57,58,59]; (**b**) homeostatic model assessment for insulin resistance (HOMA-IR) [24,25,26,29,30,53,56,59]; (**c**) total cholesterol (TC) [1,5,22,25,26,27,30,53,54,57]; (**d**) heart rate (HR) [22,28,55]; (**e**) interleukin 6 (IL-6) [21,24,25,30]; (**f**) tumor necrosis factor-α (TNF- α) [21,24,25,30].

FPG was significantly reduced in the ginseng group compared to the placebo group (WMD: −7.03 mg/dL; 95% CI: −10.89, −3.17; *p* < 0.001; phet < 0.001, I^2^ = 91%) (Figure 2a) [1,5,12,21,22,24,25,26,27,29,30,53,54,56,57,58,59]. However, there was no significant difference in OGTT (WMD: −6.81 mg/dL; 95% CI: −16.77, 3.14; *p* = 0.180; phet = 0.002, I^2^ = 66.3%) [12,25,26,27,30,53,56,59], and HbA1c (WMD: −0.04 %; 95% CI: −0.16, 0.07; *p* = 0.449; phet < 0.001, I^2^ = 82.9%) [1,5,12,21,22,24,25,26,30,53,54,57] between groups (Supplementary Figure S1). Subgroup analyses revealed that FPG was only reduced in those with baseline FPG ≥ 126 mg/dL. A significant decrease in FPG was also observed in participants who consumed less than 2 g/day of ginseng for a shorter time (8 weeks or less). However, no statistical difference between groups was observed based on the duration and dose of intervention in OGTT levels. In addition, there were no statistical differences observed between the two groups for the HbA1c baseline values, dose, and duration of supplementation in HbA1c percentage (Table 3).

##### Insulin Resistance and Secretion

Our results showed a significant decrease in HOMA-IR (WMD: −0.44; 95% CI: −0.84, −0.04; *p* = 0.03; phet = 0.001, I^2^ = 67.6%) (Figure 2b) [24,25,26,29,30,53,56,59]. However, our results indicated no difference in fasting insulin levels (WMD: −0.13 µU/mL; 95% CI: −1.18, 0.90; *p* = 0.796; phet < 0.001, I^2^ = 89.8%) [5,12,22,24,25,26,27,29,30,53,56,57,59] between the groups (Supplementary Figure S1). The subgroup analyses showed that ginseng consumption was associated with a significant reduction in HOMA-IR irrespective of the follow-up length. Additionally, subgroup analyses did not reveal any differences in fasting insulin irrespective of ginseng interventional dosage and length of follow-up (Table 3).

#### 3.3.3. Cardiovascular Risk Factors

##### Lipid Profile

Pooling of 13 effect sizes showed a significant reduction in TC concentrations between ginseng and placebo groups at follow-up (WMD: −5.77 mg/dL; 95% CI: −11.53, −0.01; *p* = 0.04; phet < 0.001, I^2^ = 80.8%) (Figure 2c) [1,5,22,25,26,27,30,53,54,57]. However, ginseng supplementation had no effect on TG (WMD: 6.05 mg/dL; 95% CI: −4.37, 16.48; *p* = 0.255; phet < 0.001, I^2^ = 73.5%) [1,22,25,26,27,30,53,54,57], LDL-C (WMD: −4.16 mg/dL; 95% CI: −8.98, 0.65; *p* = 0.090; phet < 0.001, I^2^ = 78.2%) [1,5,22,25,26,27,30,53,54,57], and HDL-C (WMD: −1.28 mg/dL; 95% CI: −5.58, 3.01; *p* = 0.557; phet < 0.001, I^2^ = 96.9%) [1,5,22,25,26,27,30,53,54,57] in a pooled analysis of 12, 13, and 13, respectively (Supplementary Figure S1). On subgroup analyses, we found that ginseng supplementation significantly reduced serum TC levels in studies that included participants with baseline levels of TC below 200 mg/dL and studies with supplementation dose of ginseng at 2 g/day or above. Also, studies with 2 g/day or a higher dose of ginseng supplementation reported a reduction in serum LDL-C levels. Interestingly, TG concentration significantly elevated following ginseng supplementation in studies involving individuals with baseline TG levels below 150 mg/dL. However, there was no significant reduction in HDL-C levels in the ginseng group compared to the control group, irrespective of baseline levels of HDL-C, supplementation dose, and follow-up duration (Table 3).

##### Blood Pressure and Heart Rate

In a meta-analysis of three studies, HR significantly increased in the intervention group compared to the control group (WMD: 2.65 bpm; 95% CI: 2.20, 3.09; *p* < 0.001; phet = 0.363, I^2^ = 1.2%) (Figure 2d) [22,28,55]. However, there were no significant differences in SBP (WMD: −2.78 mmHg; 95% CI: −6.97, 1.40; *p* = 0.193; phet < 0.001, I^2^ = 87.4%) [5,12,22,24,25,28,30,53] and DBP values (WMD: −0.24 mmHg; 95% CI: −1.88, 1.39; *p* = 0.770; phet = 0.003, I^2^ = 63.4%) [5,12,22,24,25,28,30,53] between the ginseng and control groups (Supplementary Figure S1). Subgroup analyses did not reveal any differences in SBP and DBP regardless of baseline values, dose, and duration of intervention. Furthermore, subgroup analysis for HR was not possible due to the limited number of studies (n = 3) (Table 3).

#### 3.3.4. Inflammatory Markers and Adipocytokines

Ginseng administration significantly reduced serum IL-6 levels (WMD: −1.22 pg/mL; 95% CI: −1.68, −0.75; *p* < 0.001; phet = 0.230, I^2^ = 27.3%) (Figure 2e) [21,24,25,30] while increasing TNF-α concentration (WMD: 2.15 pg/mL; 95% CI: 0.66, 3.63; *p* = 0.005; phet = 0.920, I^2^ = 0.0%) (Figure 2f) [21,24,25,30] at follow-up. However, there was no significant between-group difference regarding CRP (WMD: −0.10 µg/mL; 95% CI: −0.61, 0.41; *p* = 0.696; phet = 0.046, I^2^ = 55.8%) [21,24,25,30], adiponectin (WMD: −0.27µg/mL; 95% CI: −1.41, 0.86; *p* = 0.639; phet = 0.906, I^2^ = 0.0%) [30], and leptin (WMD: −0.67 pg/mL; 95% CI: −2.01, 0.65; *p* = 320; phet = 0.847, I^2^ = 0.0%) (Supplementary Figure S1) [30]. Subgroup analysis was not conducted for CRP, IL-6, TNF-α, adiponectin, or leptin, as there were not enough studies reported on these parameters (Table 3).

#### 3.3.5. Liver Function Tests

Ginseng supplementation did not affect ALT (WMD: 0.62 U/L; 95% CI: −1.90, 3.15; *p* = 0.630; phet = 0.085, I^2^ = 46.0%) [5,22,23,30], AST (WMD: −0.28 U/L; 95% CI: −1.77, 1.19; *p* = 0.704; phet = 0.272, I^2^ = 22.3%) [12,23,30], and GGT (WMD: 2.03 U/L; 95% CI: −6.22, 10.28; *p* = 0.630; phet = 0.285, I^2^ = 20.3%) (Supplementary Figure S1) [30]. However, subgroup analyses were not performed for ALT, AST, or GGT due to the limited number of studies that reported liver function tests.

### 3.4. Sensitivity Analysis

Sensitivity analysis for HOMA-IR showed that the overall estimates were affected by the exclusion of the studies conducted by Ma et al. [59] (WMD: −0.15, 95% CI: −0.37, 0.07) and Park et al. [56] (WMD: −0.33, 95% CI: −0.71, 0.04). Exclusion of studies carried out by Bang et al. [53] (WMD: −4.70 mg/dL, 95% CI: −12.58, 3.17), Oh et al. [27] (WMD: −4.17 mg/dL, 95% CI: −10.15, 1.79), Choi et al. [26] (WMD: −5.91 mg/dL, 95% CI: −12.16, 0.33), Vuksan et al. [5] (WMD: −4.25 mg/dL, 95% CI: −10.18, 1.67), and Jovanovski et al. [22] (WMD: −4.72 mg/dL, 95% CI: −10.79, 1.34) changed the overall effect size for TC. Furthermore, the results of the sensitivity analysis for TG showed that removing the Jovanovski et al. [22] study changed the overall effect size (WMD: 9.99 mg/dL, 95% CI: 0.32, 19.66). Furthermore, overall estimates for LDL-C were influenced by the exclusion of a study performed by Yoon et al. (A) [30] (WMD: −4.88 mg/dL, 95% CI: −9.73, −0.04). Additionally, the exclusion of Hosseini et al. [21] study also changed the overall effect size for IL-6 (WMD: −0.57 pg/mL, 95% CI: −1.84, 0.69). Finally, sensitivity analysis for BW, BMI, WC, FPG, HbA1c, OGTT, fasting insulin, HDL-C, SBP, DBP, HR, CRP, TNF-α, adiponectin, leptin, ALT, AST, and GGT did not indicate any evidence of sensitivity.

### 3.5. Publication Bias

Based on visual inspection of funnel plots (Supplementary Figure S2), as well as Egger’s [60] and Begg’s [61] statistical tests (Supplementary Table S3), we found no evidence of publication bias for BW, BMI, WC, HbA1c, OGTT, fasting insulin, HOMA-IR, TG, TC, LDL-C, HDL-C, SBP, HR, CRP, TNF-α, adiponectin, leptin, ALT, AST, and GGT. The Funnel plot and Egger’s test manifested that there was publication bias for FPG (*p* = 0.03), DBP (*p* = 0.05), and IL-6 (*p* = 0.006).

### 3.6. Non-Linear Dose-Response between Dose and Duration of Ginseng Supplementation on Cardiometabolic Indicators

Dose-response analysis showed that ginseng supplementation significantly altered HOMA-IR based on dose (r = −0.26, *p*-nonlinearity = 0.02) and study duration (r = 7.18, *p*-nonlinearity = 0.04) in a non-linear fashion. Furthermore, the dose of ginseng affected HDL-C (r = −0.31, *p*-nonlinearity = 0.009) and duration of intervention affected LDL-C (r = −16.61, *p*-nonlinearity = 0.04) in a non-linear fashion. No significant associations were observed for other outcomes in non-linear dose-responses (Table 4), (Supplementary Figures S3 and S4).

### 3.7. Meta-Regression Analysis

Meta-regression using the random-effects model was undertaken to investigate the potential association between a change in cardiometabolic indicators and the dose of ginseng (g/day) and the duration of the trial. Meta-regression analysis indicated a linear relationship between dose absolute changes in HDL-C (*p* = 0.02) but not for other studied outcomes (Table 4), (Supplementary Figures S5 and S6).

### 3.8. GRADE Assessment

An evaluation of the quality of evidence using the GRADE approach is presented in Table 5. For HR and TNF-α, the quality of evidence was high since most studies had a low to moderate risk of bias with low statistical and clinical heterogeneity and narrow CIs. Moreover, the quality of evidence for TC and HOMA-IR was deemed moderate due to inconsistency (I^2^ = 80.8% and I^2^ = 67.6% for heterogeneity, respectively). In addition, the evidence regarding IL-6 was determined to be of moderate quality, owing to serious publication bias (*p* = 0.006). Moreover, there was moderate evidence on BW, BMI, WC, adiponectin, leptin, ALT, AST, and GGT, owing to serious imprecision (wide CI). For HbA1c, OGTT, fasting insulin, TG, LDL-C, HDL-C, SBP, DBP, and CRP, the evidence was deemed low quality due to serious inconsistency (I^2^ = 82.9% (HbA1c), I^2^ = 66.3% (OGTT), I^2^ = 89.8% (fasting insulin), I^2^ = 73.5% (TG), I^2^ = 78.2% (LDL-C), I^2^ = 96.9% (HDL-C), I^2^ = 87.4% (SBP), I^2^ = 63.4% (DBP), and I^2^ = 55.8% (CRP) for heterogeneity) and imprecision (Wide CI). Finally, for FPG, the quality of evidence was also low due to serious inconsistency (I^2^ = 91.0% for heterogeneity) and publication bias (*p* = 0.03).

## 4. Discussion

This meta-analysis evaluated the effects of ginseng supplementation on cardiovascular biomarkers, including anthropometric indices, glycemic and lipid profiles, blood pressure (BP), inflammatory biomarkers, adipocytokines, and liver function indicators among subjects with prediabetes and T2DM. According to the findings of this study, ginseng consumption was associated with a reduction in FPG, HOMA-IR, TC, and IL-6, and escalations in HR and TNF-α, without any significant alterations in anthropometric measurements (BW, BMI, and WC), glycemic responses (OGTT, HbA1c, and fasting insulin), lipid profile (TG, LDL-C, and HDL-C), BP (SBP and DBP), inflammatory markers and adipocytokines (CRP, adiponectin, and leptin), and liver enzymes (ALT, AST, and GGT) when compared with a control group.

A comprehensive review of human trials and in vitro and in vivo studies suggests that ginseng modulates insulin secretion, glucose uptake, and glucose metabolism through inhibition of β-cell apoptosis and raising the production of glucagon-like peptide-1 (GLP-1) to exert anti-diabetic effects [11]. However, in 2011, a systematic review and meta-analysis of four RCTs showed that ginseng intake did not change blood glucose-related indices in patients with T2DM [13]. Furthermore, data from two meta-analyses on the general population suggested conflicting reports regarding ginseng supplementation effects on FPG levels. One study reported significantly reduced levels of FPG following ginseng intake [6], and the other indicated no effect [34]; both confirmed that there was no significant effect of ginseng consumption on fasting plasma insulin and HbA1c. Similar to our results, a recent experimental study revealed that Panax quinquefolius decreased FPG levels and improved insulin resistance (IR) in T2DM [62]. In addition, Gui et al. [63], based on a meta-analysis of eight RCTs, reported that FPG and HOMA-IR were improved by ginseng consumption with no change in OGTT, HbA1c, and fasting insulin in patients with T2DM. Likewise, we extended these findings by pooling the results of 24 effect sizes (n = 1295 participants) and showed that ginseng supplementation significantly improved FPG stronger when the consumption lasted for 8 weeks or more and significantly reduced HOMA-IR values regardless of the length of follow-up. Moreover, as seen in our subgroup analyses, ginseng supplementation had a beneficial effect on the concentration of FPG for either patient with a baseline level of FPG ≥ 126 mg/dL or when the supplementation dosage of ginseng was less than two g/day. Therefore, it seems that the effect of ginseng intake on FPG depends on baseline levels of FPG, dose, and duration of intervention. In dose-response analyses, lower duration and dose of ginseng consumption had a greater lower effect on HOMA-IR values. It is noteworthy that the discrepancies between meta-analyses could be due to different numbers of included studies and various studied populations.

Insulin resistance is the driving factor that leads to the development of T2DM [64]. Long-term IR in adipocytes leads to elevated free fatty acids (FFAs) and an accelerated TG formation, which contributes to dyslipidemia in patients with T2DM [65,66]. A series of pharmacological investigations suggested hypolipidemic effects of ginseng administration, mainly through activating AMP-activated protein kinase (AMPK) among individuals with prediabetes and T2DM [5,22,62,67]. In this study, we have shown that consumption of ginseng reduced TC levels stronger among those with baseline TC < 200 mg/dL and intake of ≥ 2 g/day. Additionally, we found that patients with prediabetes and T2DM had a significantly lower level of LDL-C after consuming ≥2 g/day of ginseng. The subgroup analysis in the current study also suggests that ginseng supplementation reduces serum levels of TG in individuals with baseline TG values < 150 mg/dL. The underlying mechanisms of the lipid-lowering effects of ginseng are still unclear. However, we assumed a possible reason for the relationship between ginseng intake and levels of lipid profile components in our meta-analysis. The steroidal structure of triterpene saponins may alter gene transcription, protein synthesis, and cholesterol production in the liver through inhibition of β-Hydroxy β-methylglutaryl-CoA (HMG-CoA) reductase [68,69], which was also reported as the possible anti-diabetic mechanism of some citrus flavonoids [70]. The meta-regression and dose-response analyses also revealed that the longer the study duration, the greater the effect of ginseng supplementation on lowering LDL-C. The higher the dose, the greater the effect of ginseng supplementation on lowering HDL-C. We found a disparity in findings obtained from previous meta-analyses investigating the efficacy of ginseng administration on lipid profile parameters. One suggested no lipid-lowering effects of ginseng consumption from three previous meta-analyses conducted on healthy and unhealthy individuals [36], while others indicated significant exerted effects of ginseng intake [32,34]. Additionally, a previous meta-analysis that investigated ginseng supplementation in patients with T2DM demonstrated a significant effect of ginseng supplementation on serum TC, TG, and LDL-C levels [63]. The disagreements may be due to different types of ginseng supplements, different target populations, and a limited number of studies included in previous reviews.

Another aspect of the pathogenesis of T2DM is low-grade chronic inflammation. The inflammatory process contributes to IR and, consequently, to T2DM-associated cardiovascular complications [71]. Elevated levels of TNFα have been shown to directly affect insulin receptor signaling and decrease insulin sensitivity [71,72]. Several molecular pathways could be influenced by ginsenosides, manifesting anti-inflammatory effects. The most notable anti-inflammatory mechanisms are inhibition of toll-like receptor four signaling pathway, inhibition of NF-κB signaling pathway, activation of AMPK, and increased nuclear factor erythroid-2-related factor 2 (Nrf2) expression and translocation [73]. A growing body of evidence proposed that ginseng treatment significantly inhibits the expression of inflammatory factors and exerts a protective effect in patients with prediabetes and T2DM [74,75,76]. However, the recent meta-analyses in the general population did not show significant overall effects of ginseng consumption on CRP levels [33,35]. The second study also demonstrated significant reductions in IL-6 and TNF-α following ginseng supplementation [33]. Notably, the current study is the first systematic review and meta-analysis investigating the effectiveness of ginseng supplementation on inflammatory biomarkers in subjects with prediabetes and T2DM. Our meta-analysis was in line with the previous ones concerning CRP and IL-6 changes. However, we observed significantly elevated levels of TNF-α after ginseng consumption. The results of this study should be interpreted cautiously, and more research should be conducted on the effects of ginseng supplementation on inflammatory markers. Future long-term dose-escalation studies are necessary since the TC, HOMA-IR, and IL-6 findings were not robust due to their sensitivity.

Obesity is believed to be a promoter of T2DM [77]. Increased BMI is associated with higher leptin levels, one of the major adipokines released by adipocytes [72]. It has already been known that ginsenosides inhibit adipogenesis and lipid accumulation in adipocytes [78]. The current study is the first report investigating the effects of ginseng administration on anthropometric indices and adipocytokines among subjects with prediabetes and T2DM. Our meta-analysis showed that ginseng consumption did not affect anthropometric measurements (BW, BMI, and WC) and adipocytokines (adiponectin and leptin) in individuals with prediabetes and T2DM. The findings are consistent with previous studies [19,34], suggesting that ginseng intake did not significantly differ in anthropometric measurements between the intervention and placebo groups in the general population. The findings from the present study were concluded in a relatively small sample size (n = 648 participants). Therefore, if well-designed clinical studies establishing appropriate inclusion criteria and larger sample sizes are documented, significant results are not far from expectation on these markers.

We have also demonstrated that consumption of ginseng significantly accelerated the HR. However, its effects on SBP and DBP among individuals with prediabetes and T2DM were insignificant. Also noteworthy is that the present study represents the first meta-analysis to investigate the effects of ginseng on BP and HR in people with prediabetes and type 2 diabetes. In preclinical evidence, ginseng supplementation decreased the BP through the activation of endothelial nitric oxide synthase and the release of nitric oxide and sped up the HR [79,80]. A systematic review and meta-analysis study in subjects with hypertension revealed significantly reduced BP levels following ginseng intake. However, the total sample size was insufficient to draw conclusions [81]. Similarly, another review record on healthy and unhealthy individuals showed the same results [34]. However, non-significant levels of SBP and DBP were observed after ginseng supplementation in the general population with a larger sample size than the previous ones in a meta-analysis setting [82]. Likewise, the findings do align with those of our meta-analysis. Although we can consider the HR acceleration as a side effect of ginseng consumption, the limited number of included studies necessitates more direct investigations.

Based on our findings, ginseng supplementation did not change measures of hepatic function in patients with prediabetes and T2DM. Nevertheless, some evidence has suggested that ginseng has favorable impacts on hepatocellular function through its anti-inflammatory, anti-oxidative, and anti-apoptotic properties [83]. In addition, a recent clinical trial study on individuals with hepatic dysfunction reported that ginseng supplementation significantly changes liver function enzymes level [84]. Our study confirmed the earlier meta-analysis study, which found that ginseng did not appear to have hepatoprotective effects in the general population [31]. Finally, it is noteworthy that we could not perform a subgroup analysis for any outcome based on the different forms of ginseng supplementation (extract vs. powder) since most of the included studies intervened extract form of ginseng. A study of 4-week supplementation of fermented red ginseng showed that only glucose values following oral glucose tolerance test were lowered, without any significant changes in FPG following ginseng supplementation [27]. In contrast, in another study with the administration of ginseng extract, FPG concentrations decreased [5]. As the extract form of any supplement has higher bioavalibity than the powder form, we can hypothesize that studies with extraction form have more promising findings.

## 5. Strengths and Limitations

In this paper, we included multiple endpoints to provide a comprehensive overview of the effects of ginseng on cardiometabolic parameters in individuals with prediabetes and T2DM. Both parallel and crossover randomized trials written in the English language were included. Additionally, we conducted dose-response and meta-regression analyses to assess the association between pooled effect size, dosage, and duration of ginseng supplementation. Subgroup analyses were also conducted to further explore each listed outcome’s results. Finally, we graded the overall certainty of evidence across the studies according to the GRADE guidelines. Despite the above strengths, the present study is not without limitations. First, the sample sizes of the included studies were also relatively small, with only one study including more than 100 participants. Second, relatively half of the studies were conducted in Asia, limiting generalizability. Third, some factors, such as duration of diabetes and smoking status, may influence the cardiovascular risk and should also be considered confounders, but were not included in the analysis due to poor reporting of these variables. Fourth, statistical heterogeneity is apparent in our analysis. This may be attributed to the poor methodological quality and/or differences in treatment regimens (doses/durations) or the ginseng type used.

## 6. Conclusions

This meta-analysis suggests that ginseng can improve cardiometabolic outcomes in individuals with prediabetes and type 2 diabetes. These results may provide important information to health agencies in formulating future guidelines for the use of ginseng in managing diabetes and the associated risk factors and preventing the progression of prediabetes. However, large-scale, well-designed RCTs should be performed to further verify these findings in the future.

## Figures and Tables

**Table 1:** PICOS criteria for inclusion of studies.

Parameter	Inclusion Criteria
Population	Individuals older than 18 years and with physician’s diagnosis of impaired glucose tolerance or prediabetes or T2DM
Intervention	Administration of ginseng in different chemical forms including capsule, tablet, and powder
Comparator	Comparison with placebo, usual care, or any pharmacological or non-pharmacological intervention(s)
Outcome	Those which reported mean changes and their standard deviations (SDs) of anthropometric indices (weight, BMI, and WC), glycemic control parameters (FPG, insulin, HbA1c, OGTT, and HOMA-IR), lipid profile components (TG, TC, LDL-C, and HDL-C), blood pressure (SBP and DBP), HR, inflammatory biomarkers and adipokines (CRP, IL-6, TNF-α, adiponectin, and leptin), and liver function tests (ALT, AST, and GGT) throughout the trial for both intervention and control groups or those presenting the required information to calculate these effect sizes
Study design	Being an RCT in either parallel or cross-over design

Abbreviations: T2DM—type 2 diabetes mellitus; SDs—standard deviations; BMI—body mass index; WC—waist circumference; FPG—fasting plasma glucose; HbA1c—hemoglobin A1c; OGTT—oral glucose tolerance test; HOMA-IR—homeostatic model assessment of insulin resistance; TG—triglyceride; TC—total cholesterol; LDL-C—low-density lipoprotein cholesterol; HDL-C—high-density lipoprotein cholesterol; SBP—systolic blood pressure; DBP—diastolic blood pressure; HR—heart rate; CRP—C-reactive protein; IL-6—interlukin-6; TNF-α—tumor necrosis factor-α; ALT—alanine aminotransferase; AST—aspartate aminotransferase; GGT—gamma-glutamyl transferase; RCT—randomized controlled trial.

**Table 2:** Characteristics of the included studies.

Studies	Country	Study Design	Participant	Sex	Sample Size		Trial Duration (Week)	Means Age		Means BMI		Intervention		
					IG	CG		IG	CG	IG	CG	Ginseng Dose	Ginseng Type	Control Group
Sotaniemi et al., 1995 (A) [58]	Finland	paralell, R, PC, DB	T2DM	M/F (8, 10)	12	6	8	59 ± 7	60 ± 6	NR	NR	0.1	Ginseng extract	Placebo
	Finland	paralell, R, PC, DB	T2DM	M/F (8, 10)	12	6	8	57 ± 9	60 ± 6	NR	NR	0.2	Ginseng extract	Placebo
Sotaniemi et al., 1995 (B) [58]	Canada	cross-over, R, PC, DB	T2DM	M/F (11, 8)	9	10	12	64 ± 6	64 ± 6.32	28.9 ± 4.2	28.9 ± 4.42	6	Panax ginseng	Placebo
	Hong Kong	cross-over, R, PC, DB	T2DM	M/F	10	10	4	51.5 ± 6	51.5 ± 6	28.5 ± 4.11	28.5 ± 4.11	2.214	Panax ginseng	Placebo
Vuksan et al., 2008 [12]	USA	paralell, R, PC, DB	T2DM	M/F (7, 1)	5	3	4	46 ± 6.7	46 ± 5.19	31 ± 2.23	36 ± 3.46	8	Panax ginseng	Placebo
	USA	paralell, R, PC, DB	T2DM	M (7)	5	2	4	46 ± 6.7	46 ± 4.24	35 ± 6.70	36 ± 2.82	0.35	Panax ginseng	Placebo
Ma et al., 2008 [59]	South Korea	paralell, R, PC, DB	T2DM	M/F (13, 11)	18	6	8	52.7 ± 11	54.8 ± 10	26.3 ± 4.8	25.3 ± 1.9	1.5	Panax ginseng	Placebo
	South Korea	paralell, R, PC, DB	T2DM	M/F (15, 9)	18	6	8	52.7 ± 10	54.8 ± 10	24 ± 2.6	25.3 ± 1.9	2	Panax ginseng	Placebo
Reedes et al., 2011 (A) [57]	South Korea	paralell, R, PC, DB	T2DM	M/F (16, 8)	18	6	8	51.1 ± 8.6	54.8 ± 10	25.4 ± 2.7	25.3 ± 1.9	3	Panax ginseng	Placebo
	Iran	paralell, R, PC, DB	T2DM	M/F (14, 16)	15	15	8	48.1 ± 5.7	46 ± 4.5	29.9 ± 3	30.6 ± 2.8	0.3	Panax ginseng	Placebo
Reedes et al., 2011 (B) [57]	Croatia	paralell, R, PC, DB	T2DM & HTN	M/F (22, 42)	30	34	12	62.1 ± 8.8	63.9 ± 10.93	33.4 ± 5.6	29.9 ± 4.95	3	Panax quinquefolius	Placebo
	South Korea	paralell, R, PC, DB	Impaired fasting glucose	M/F (10, 10)	11	9	8	50.45 ± 12.36	44.56 ± 10.48	NR	NR	0.96	Panax ginseng	Placebo
Yoon et al., 2012 (A) [30]	South Korea	paralell, R, PC, DB	T2DM	M/F (28, 13)	21	20	12	58.81 ± 1.72	56.1 ± 2.18	23.52 ± 0.48	23.8 ± 0.61	5	Panax ginseng	Placebo
	South Korea	paralell, R, PC, DB	Impaired fasting glucose/T2DM	M/F (28, 14)	21	21	4	53.2 ± 1.8	53.5 ± 1.9	24.9 ± 0.7	24.9 ± 0.8	2.7	Panax ginseng	Placebo
Yoon et al., 2012 (B) [30]	Croatia	paralell, R, PC, DB	T2DM	M/F (28, 46)	35	39	12	61.9 ± 8.59	63.7 ± 10.28	33 ± 5.53	30.1 ± 4.68	3	Panax quinquefolius	Placebo
	Iran	paralell, R, PC, DB	T2DM	M/F (12, 28)	20	20	8	47.9 ± 4.7	47.3 ± 6.4	29.29 ± 3.61	27.19 ± 4.71	0.3	Panax ginseng	Placebo
Yoon et al., 2012 (C) [30]	Iran	paralell, R, PC, DB	T2DM	M/F (11, 34)	23	22	6	47.9 ± 4.7	47.3 ± 6.4	29.29 ± 3.61	27.19 ± 4.71	0.3	Ginseng extract	Placebo
	South Korea	paralell, R, PC, DB	T2DM	M/F (42, 30)	34	38	12	52.76 ± 10.24	51.89 ± 9.46	25.52 ± 2.87	25.61 ± 3.05	1	Panax ginseng	Placebo
Hosseini et al., 2013 [54]	Canada	cross-over, R, PC, DB	T2DM	M/F (13, 11)	12	12	8	64 ± 7	64 ± 7	27.8 ± 4.6	27.8 ± 4.6	3	Panax quinquefolius	Placebo
	Australia	paralell, R, PC, DB	Prediabetes	M/F (153, 248)	202	199	24	53.4 ± 10	53.65 ± 10.4	34.7 ± 6.5	34.6 ± 6.0	0.32	Panax ginseng	Placebo
Mucalo et al., 2013 [28]	South Korea	paralell, R, PC, DB	T2DM	M/F (35, 24)	28	31	24	61.2 ± 8.45	60.9 ± 7.23	24.5 ± 2.90	24.8 ± 2.82	1	Panax ginseng	Placebo
	South Korea	paralell, R, PC, DB	T2DM nephropathy	M/F (36, 25)	30	31	24	59.3 ± 8.79	59.7 ± 7.22	24.7 ± 2.93	24.8 ± 2.82	3	Panax ginseng	Placebo
Park et al., 2014 [56]	Canada and Croatia	paralell, R, PC, DB	T2DM & HTN	M/F (49, 31)	43	37	12	59.44 ± 7.4	60.58 ± 6.9	28.62 ± 3.4	29.66 ± 4.3	6.75	Panax ginseng and Panax quinquefolius	Placebo
	Canada and Croatia	paralell, R, PC, DB	T2DM & HTN	M/F (49, 31)	43	37	12	59.44 ± 7.4	60.58 ± 6.9	28.62 ± 3.4	29.66 ± 4.3	2.25	Panax ginseng and Panax quinquefolius	Placebo

Abbreviations: BMI— body mass index; IG—intervention group; CG—control group; R—randomized; PC—placebo controlled; DB—double blind; T2DM—type 2 diabetes mellitus; M—male; F—female; NR—not reported; HTN—hypertention.

**Table 3:** Subgroup analyses of ginseng supplementation on cardiovascular risk factors in patients with prediabetes and T2DM.

	Number of Studies	WMD (95% CI)	*p*-Value	Heterogeneity	
				*p* Heterogeneity	I^2^
Subgroup analyses of ginseng supplementation on BW					
Overall effect	5	−0.54 (−2.54, 1.46)	0.598	0.998	0.0%
Subgroup analyses of ginseng supplementation on BMI					
Overall effect	9	0.05 (−0.26, 0.38)	0.717	0.963	0.0%
Baseline BMI (kg/m^2^)					
Normal (18.5–24.9)	3	0.118 (−0.37, 0.60)	0.639	0.605	0.0%
Overweight (25–29.9)	3	0.17 (−0.34, 0.69)	0.501	0.961	0.0%
Obese (>30)	3	−0.31 (−1.05, 0.42)	0.405	0.931	0.0%
Trial duration (week)					
≤8	6	0.10 (−0.31, 0.53)	0.617	0.991	0.0%
>8	3	−0.00 (−0.49, 0.48)	0.983	0.404	0.0%
Supplementation dose (g/day)					
<2	5	0.08 (−0.36, 0.52)	0.713	0.694	0.0%
≥2	4	0.03 (−0.43, 0.49)	0.888	0.976	0.0%
Subgroup analyses of ginseng supplementation on WC					
Overall effect	3	0.05 (−1.16, 1.27)	0.929	0.729	0.0%
Subgroup analyses of ginseng supplementation on FPG					
Overall effect	21	−7.03 (−10.89, −3.17)	<0.001	<0.001	91.0%
Baseline FPG (mg/dL)					
≥126	11	−9.69 (−16.70, −2.69)	0.007	<0.001	73.9%
<126	7	−1.51 (−4.06, 1.04)	0.247	<0.001	77.9%
Trial duration (week)					
≤8	14	−10.71 (−16.23, −5.18)	<0.001	<0.001	83.9%
>8	7	−0.21 (−2.33, 1.90)	0.842	0.040	54.6%
Supplementation dose (g/day)					
<2	11	−8.72 (−15.53, −1.90)	0.012	<0.001	95.1%
≥2	10	−3.32 (−6.81, 0.17)	0.063	0.044	48.1%
Subgroup analyses of ginseng supplementation on OGTT					
Overall effect	10	−6.81 (−16.77, 3.14)	0.180	0.002	66.3%
Trial duration (week)					
≤8	6	−7.17 (−22.60, 8.25)	0.362	0.009	67.3%
>8	4	−3.63 (−10.00, 2.74)	0.264	0.865	0.0%
Supplementation dose (g/day)					
<2	3	−5.75 (−31.60, 20.10)	0.663	0.001	85.6%
≥2	7	−6.20 (−12.55, 0.14)	0.055	0.402	3.0%
Subgroup analyses of ginseng supplementation on HbA1c					
Overall effect	15	−0.04 (−0.16, 0.07)	0.449	<0.001	82.9%
Baseline HbA1c (%)					
<6.5	6	0.04 (−0.01, 0.09)	0.117	0.278	20.6%
≥6.5	9	−0.08 (−0.28, 0.11)	0.417	<0.001	78.5%
Trial duration (week)					
≤8	8	0.03 (−0.30, 0.37)	0.838	<0.001	80.8%
>8	7	−0.04 (−0.14, 0.05)	0.397	<0.001	75.2%
Supplementation dose (g/day)					
<2	7	−0.07 (−0.27, 0.12)	0.476	<0.001	88.4%
≥2	8	−0.00 (−0.19, 0.18)	0.962	0.001	71.2%
Subgroup analyses of ginseng supplementation on fasting insulin					
Overall effect	16	−0.13 (−1.18, 0.90)	0.796	<0.001	89.8%
Trial duration (week)					
≤8	10	0.49 (−1.12, 2.11)	0.547	0.023	53.4%
>8	6	−0.74 (−1.72, 0.24)	0.140	0.003	71.6%
Supplementation dose (g/day)					
<2	6	−0.38 (−1.83, 1.06)	0.601	0.154	<0.001
≥2	10	0.02 (−1.31, 1.36)	0.974	<0.001	93.4%
Subgroup analyses of ginseng supplementation on HOMA-IR					
Overall effect	10	−0.44 (−0.84, −0.04)	0.030	0.001	67.6%
Trial duration (week)					
≤8	6	−1.15 (−2.17, −0.13)	0.027	0.037	57.7%
>8	4	−0.05 (−0.09, −0.00)	0.022	0.899	0.0%
Supplementation dose (g/day)					
<2	5	−0.41 (−1.05, 0.21)	0.196	0.084	51.3%
≥2	5	−0.59 (−1.34, 0.16)	0.126	0.001	77.6%
Subgroup analyses of ginseng supplementation on TG					
Overall effect	12	6.05 (−4.37, 16.48)	0.255	<0.001	73.5%
Baseline TG (mg/dL)					
<150	6	17.23 (12.25, 22.21)	<0.001	0.479	0.0%
≥150	6	−2.68 (−22.50, 17.14)	0.791	0.010	67.0%
Trial duration (week)					
≤8	7	9.69 (−3.36, 22.75)	0.146	0.002	71.9%
>8	5	−4.45 (−29.17, 20.25)	0.724	0.001	78.9%
Supplementation dose (g/day)					
<2	5	6.61 (−7.46, 20.68)	0.357	0.157	39.6%
≥2	7	4.76 (−9.14, 18.67)	0.502	0.001	72.1%d
Subgroup analyses of ginseng supplementation on TC					
Overall effect	13	−5.77 (−11.53, −0.01)	0.049	<0.001	80.8%
Baseline TC (mg/dL)					
≥200	7	−3.84 (−16.26, 8.57)	0.544	<0.001	89.1%
<200	6	−7.04 (−10.78, −3.31)	<0.001	0.323	14.2%
Trial duration (week)					
≤8	8	−5.64 (−15.98, 4.69)	0.285	0.013	60.5%
>8	5	−4.77 (−11.94, 2.39)	0.192	<0.001	82.5%
Supplementation dose (g/day)					
<2	5	2.42 (−1.54, 6.40)	0.231	0.523	0.0%
≥2	8	−11.10 (−16.53, −5.68)	<0.001	0.004	66.5%
Subgroup analyses of ginseng supplementation on LDL-C					
Overall effect	13	−4.16 (−8.98, 0.65)	0.090	<0.001	78.2%
Baseline LDL (mg/dL)					
≥130	2	0.09 (−4.03, 4.22)	0.965	0.666	0.0%
<130	11	−5.04 (−10.22, 0.14)	0.057	<0.001	76.2%
Trial duration (week)					
≤8	8	−0.12 (−8.54, 8.29)	0.977	0.025	56.3%
>8	5	−6.59 (−13.37, 0.19)	0.057	<0.001	86.6%
Supplementation dose (g/day)					
<2	5	−0.40 (−5.55, 4.74)	0.877	0.281	21.0%
≥2	8	−6.81 (−12.73, −0.88)	0.024	<0.001	79.5%
Subgroup analyses of ginseng supplementation on HDL-C					
Overall effect	13	−1.28 (−5.58, 3.01)	0.557	<0.001	96.9%
Baseline HDL (mg/dL)					
<50	7	0.95 (−0.74, 2.64)	0.273	0.949	0.0%
≥50	6	−3.05 (−9.53, 3.42)	0.355	<0.001	98.4%
Trial duration (week)					
≤8	8	−0.14 (−1.41, 1.12)	0.822	0.998	0.0%
>8	5	−2.53 (−10.04, 4.98)	0.509	<0.001	98.6%
Supplementation dose (g/day)					
<2	5	−0.20 (−1.53, 1.12)	0.762	0.876	0.0%
≥2	8	−1.60 (−7.61, 4.40)	0.600	<0.001	97.7%
Subgroup analyses of ginseng supplementation on SBP					
Overall effect	10	−2.78 (−6.97, 1.40)	0.193	<0.001	87.4%
Baseline SBP (mmHg)					
≥130	5	−4.51 (−14.18, 5.15)	0.360	<0.001	92.8%
<130	5	−0.96 (−2.06, 0.13)	0.086	0.704	0.0%
Trial duration (week)					
≤8		−3.22 (−10.68, 4.23)	0.397	0.079	55.8%
>8		−2.59 (−7.79, 2.60)	0.328	<0.001	92.0%
Supplementation dose (g/day)					
<2	2	2.75 (−2.15, 7.67)	0.271	0.845	0.0%
≥2	8	−3.92 (−8.77, 0.91)	0.112	<0.001	89.7%
Subgroup analyses of ginseng supplementation on DBP					
Overall effect	10	−0.24 (−1.88, 1.39)	0.770	0.003	63.4%
Baseline SBP (mmHg)					
≥80	8	0.56 (−1.43, 2.56)	0.582	0.004	66.8%
<80	2	−2.63 (−6.54, 1.27)	0.186	0.081	67.2%
Trial duration (week)					
≤8	4	0.04 (−4.88, 4.96)	0.987	0.024	68.1%
>8	6	−0.20 (−1.96, 1.56)	0.821	0.010	67.0%
Supplementation dose (g/day)					
<2	2	3.00 (−0.13, 6.14)	0.061	0.998	0.0%
≥2	8	−0.93 (−2.50, 0.64)	0.247	0.024	56.6%
Subgroup analyses of ginseng supplementation on HR					
Overall effect	3	2.65 (2.20, 3.09)	<0.001	0.363	1.2%
Subgroup analyses of ginseng supplementation on CRP					
Overall effect	6	−0.10 (−0.61, 0.41)	0.696	0.046	55.8%
Subgroup analyses of ginseng supplementation on IL−6					
Overall effect	6	−1.22 (−1.68, −0.75)	<0.001	0.230	27.3%
Subgroup analyses of ginseng supplementation on TNF-α					
Overall effect	6	2.15 (0.66, 3.63)	0.005	0.920	0.0%
Subgroup analyses of ginseng supplementation on adiponectin					
Overall effect	3	−0.27 (−1.41, 0.86)	0.639	0.906	0.0%
Subgroup analyses of ginseng supplementation on leptin					
Overall effect	3	−0.67 (−2.01, 0.65)	0.320	0.847	0.0%
Subgroup analyses of ginseng supplementation on ALT					
Overall effect	7	0.62 (−1.90, 3.15)	0.630	0.085	46.0%
Subgroup analyses of ginseng supplementation on AST					
Overall effect	5	−0.28 (−1.77, 1.19)	0.704	0.272	22.3%
Subgroup analyses of ginseng supplementation on GGT					
Overall effect	3	2.03 (−6.22, 10.28)	0.630	0.285	20.3%

Abbreviations: BW—body weight; BMI—body mass index; WC—waist circumference; FPG—fasting plasma glucose; OGTT—oral glucose tolerance test; HbA1c—hemoglobin A1c; HOMA-IR—homeostatic model assessment of insulin resistance; TG—triglyceride; TC—total cholesterol; LDL-C—low-density lipoprotein cholesterol; HDL-C—high-density lipoprotein cholesterol; SBP—systolic blood pressure; DBP—diastolic blood pressure; HR—heart rate; CRP—C-reactive protein; IL-6—interlukin-6; TNF-α—tumor necrosis factor-α; ALT—alanine aminotransferase; AST—aspartate aminotransferase; GGT—gamma-glutamyl transferase.

**Table 4:** Linear and non-linear dose-responses between dose and duration of Panax supplementation and cardiometabolic biomarkers.

Biomarkers	Regression				Dose-Response			
	Dose		Duration		Dose		Duration	
	Coefficient	*p*-Value	Coefficient	*p*-Value	Coefficient	*p*-Value	Coefficient	*p*-Value
FPG	0.0806575	0.374	0.2427767	0.124	−1.755688	0.180	−13.6688	0.161
OGTT	0.075122	0.708	0.1427608	0.482	13.68284	0.356	7.016837	0.720
HbA1c	1.311766	0.588	−6.340935	0.382	0.129185	0.499	−0.5456897	0.468
Insulin	−0.1057443	0.699	0.1184111	0.853	2.118513	0.204	−1.431268	0.570
HOMA-IR	0.4107144	0.420	2.930911	0.132	−0.2646978	0.028	7.189052	0.042
TG	0.0786231	0.623	−0.0360899	0.829	0.3341626	0.750	38.14975	0.143
TC	−0.1032192	0.480	0.4331759	0.081	−2.04802	0.262	5.883329	0.606
LDL-C	−0.2034715	0.160	0.0470388	0.861	−2.107334	0.111	−16.61938	0.048
HDL-C	−0.2342807	0.020	0.021285	0.970	−0.319954	0.009	−3.99029	0.100
SBP	0.0292994	0.832	0.3400391	0.251	−11.49681	0.132	−17.22758	0.233
DBP	−0.2617145	0.317	0.8746015	0.226	−1.137181	0.077	−8.580873	0.157

Abbreviations: FPG—fasting plasma glucose; OGTT—oral glucose tolerance test; HbA1c—hemoglobin A1c; HOMA-IR—homeostatic model assessment of insulin resistance; TG—triglyceride; TC—total cholesterol; LDL-C—low-density lipoprotein cholesterol; HDL-C—high-density lipoprotein cholesterol; SBP—systolic blood pressure; DBP—diastolic blood pressure.

**Table 5:** GRADE profile of ginseng supplementation for cardiovascular biomarkers.

Outcomes	Risk of Bias	Inconsistency	Indirectness	Imprecision	Publication Bias	Number of Intervention/Control	Quality of Evidence
BW	No serious limitation	No serious limitation	No serious limitation	Serious limitation ^2^	No serious limitation	480 (244/236)	⊕⊕⊕◯ ^4^ Moderate
BMI	No serious limitation	No serious limitation	No serious limitation	Serious limitation ^2^	No serious limitation	648 (344/304)	⊕⊕⊕◯ Moderate
WC	No serious limitation	No serious limitation	No serious limitation	Serious limitation ^2^	No serious limitation	72 (54/18)	⊕⊕⊕◯ Moderate
FPG	No serious limitation	Serious limitation ^1^	No serious limitation	No serious limitation	Serious limitation ^3^	1077 (567/510)	⊕⊕◯◯ Low
OGTT	No serious limitation	Serious limitation ^1^	No serious limitation	Serious limitation ^2^	No serious limitation	347 (190/157)	⊕⊕◯◯ Low
HbA1c	No serious limitation	Serious limitation ^1^	No serious limitation	Serious limitation ^2^	No serious limitation	914 (478/436)	⊕⊕◯◯ Low
Insulin	No serious limitation	Serious limitation ^1^	No serious limitation	Serious limitation ^2^	No serious limitation	570 (306/264)	⊕⊕◯◯ Low
HOMA	No serious limitation	Serious limitation ^1^	No serious limitation	No serious limitation	No serious limitation	390 (211/179)	⊕⊕⊕◯ Moderate
TG	No serious limitation	Serious limitation ^1^	No serious limitation	Serious limitation ^2^	No serious limitation	814 (430/ 384)	⊕⊕◯◯ Low
TC	No serious limitation	Serious limitation ^1^	No serious limitation	No serious limitation	No serious limitation	838 (442/ 396)	⊕⊕⊕◯ Moderate
LDL-C	No serious limitation	Serious limitation ^1^	No serious limitation	Serious limitation ^2^	No serious limitation	838 (442/ 396)	⊕⊕◯◯ Low
HDL-C	No serious limitation	Serious limitation ^1^	No serious limitation	Serious limitation ^2^	No serious limitation	838 (442/ 396)	⊕⊕◯◯ Low
SBP	No serious limitation	Serious limitation ^1^	No serious limitation	Serious limitation ^2^	No serious limitation	420 (227/193)	⊕⊕◯◯ Low
DBP	No serious limitation	Serious limitation ^1^	No serious limitation	Serious limitation ^2^	No serious limitation	420 (227/193)	⊕⊕◯◯ Low
HR	No serious limitation	No serious limitation	No serious limitation	No serious limitation	No serious limitation	224 (116/108)	⊕⊕⊕⊕ High
CRP	No serious limitation	Serious limitation ^1^	No serious limitation	Serious limitation ^2^	No serious limitation	232 (132/100)	⊕⊕◯◯ Low
IL6	No serious limitation	No serious limitation	No serious limitation	No serious limitation	Serious limitation ^3^	232 (132/100)	⊕⊕⊕◯ Moderate
TNF-α	No serious limitation	No serious limitation	No serious limitation	No serious limitation	No serious limitation	232 (132/100)	⊕⊕⊕⊕ High
Adiponectin	No serious limitation	No serious limitation	No serious limitation	Serious limitation ^2^	No serious limitation	72 (54/18)	⊕⊕⊕◯ Moderate
Leptin	No serious limitation	No serious limitation	No serious limitation	Serious limitation ^2^	No serious limitation	72 (54/18)	⊕⊕⊕◯ Moderate
ALT	No serious limitation	No serious limitation	No serious limitation	Serious limitation ^2^	No serious limitation	269 (153/116)	⊕⊕⊕◯ Moderate
AST	No serious limitation	No serious limitation	No serious limitation	Serious limitation ^2^	No serious limitation	165 (98/67)	⊕⊕⊕◯ Moderate
GGT	No serious limitation	No serious limitation	No serious limitation	Serious limitation ^2^	No serious limitation	72 (54/18)	⊕⊕⊕◯ Moderate

^1^ There is significant heterogeneity for TG (I^2^ = 73.5%), TC (I^2^ = 80.8%), LDL-C (I^2^ = 78.2%), HDL-C (I^2^ = 96.9%), FPG (I^2^ = 91.0%), fasting insulin (I^2^ = 89.8%), HbA1c (I^2^ = 82.9%), HOMA-IR (I^2^ = 67.6%), OGTT (I^2^ = 66.3%), SBP (I^2^ = 87.4%), DBP (I^2^ = 63.4%), and CRP (I^2^ = 55.8%). ^2^ There is no evidence of significant effects (95% CI includes zero). ^3^ There is significant publication bias for FPG (*p* = 0.035) and IL-6 (*p* = 0.006). Abbreviations: BW—body weight; BMI—body mass index; WC—waist circumference; FPG—fasting plasma glucose; OGTT—oral glucose tolerance test; HbA1c—hemoglobin A1c; HOMA-IR—homeostatic model assessment of insulin resistance; TG—triglyceride; TC—total cholesterol; LDL-C—low-density lipoprotein cholesterol; HDL-C—high-density lipoprotein cholesterol; SBP—systolic blood pressure; DBP—diastolic blood pressure; HR—heart rate; CRP—C-reactive protein; IL-6—interlukin-6; TNF-α—tumor necrosis factor-α; ALT—alanine aminotransferase; AST—aspartate aminotransferase; GGT—gamma-glutamyl transferase. ^4^ ⊕ = no serious limitation; ◯ = serious limitation.

## Data Availability

The datasets generated and analyzed during the current study are available from the corresponding author on reasonable request.

## Supplementary Materials

The following supporting information can be downloaded at: https://www.mdpi.com/article/10.3390/nu14122401/s1, Figure S1: Forrest plots presenting mean difference (MD) and 95% confidence intervals for the impact of ginseng supplementation; Figure S2: Funnel plots demonstrating publication bias in the trials reporting the effect of ginseng supplementation; Figure S3: Dose-response relations between ginseng dosage (mg/day) and mean difference in each outcomes; Figure S4: Dose-response relations between duration of ginseng (week) and mean difference in each outcomes; Figure S5: Random-effects meta-regression plots of the association between dose of ginseng (mg/day) and weighted mean difference of each outcome; Figure S6: Random-effects meta-regression plots of the association between duration of ginseng (week) and weighted mean difference of each outcome; Table S1: Search terms used across the various databases; Table S2: Risk of bias assessment; Table S3: Publication bias assessment.
