# Supplementary material for: The Efficacy of Ginseng (Panax) on Human Prediabetes and Type 2 Diabetes Mellitus: A Systematic Review and Meta-Analysis
Source: Nutrients. 2022 Jun 9;14(12):2401. doi: 10.3390/nu14122401 (PMC9227417; doi:10.3390/nu14122401)
Supplement: Supplementary file 1 [file nutrients-14-02401-s001.zip › nutrients-1749831-supplementary.pdf]

Supplementary Figure S1. Forrest plots presenting mean difference (MD) and 95% confidence intervals for the impact of ginseng supplementation.

1. a

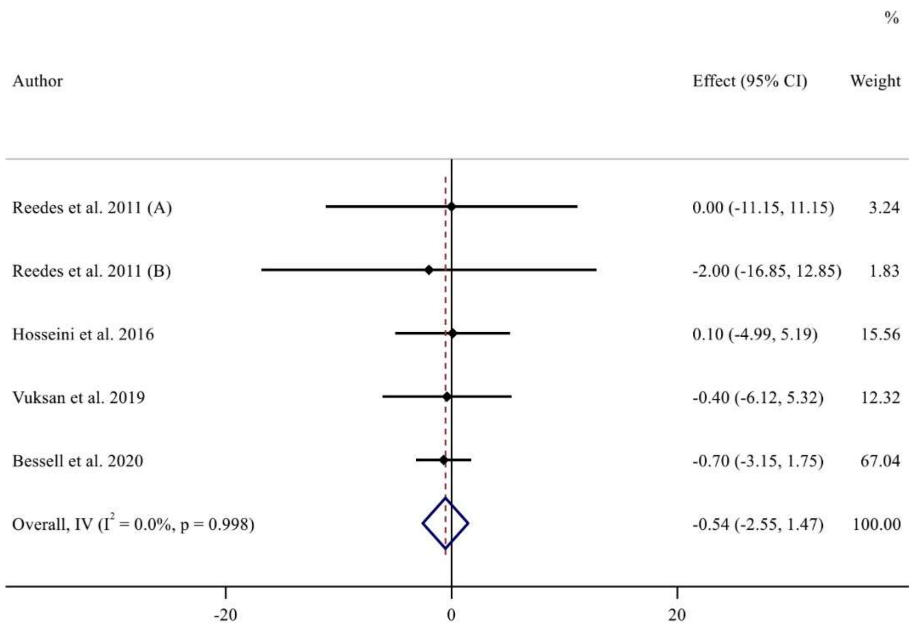

1. b

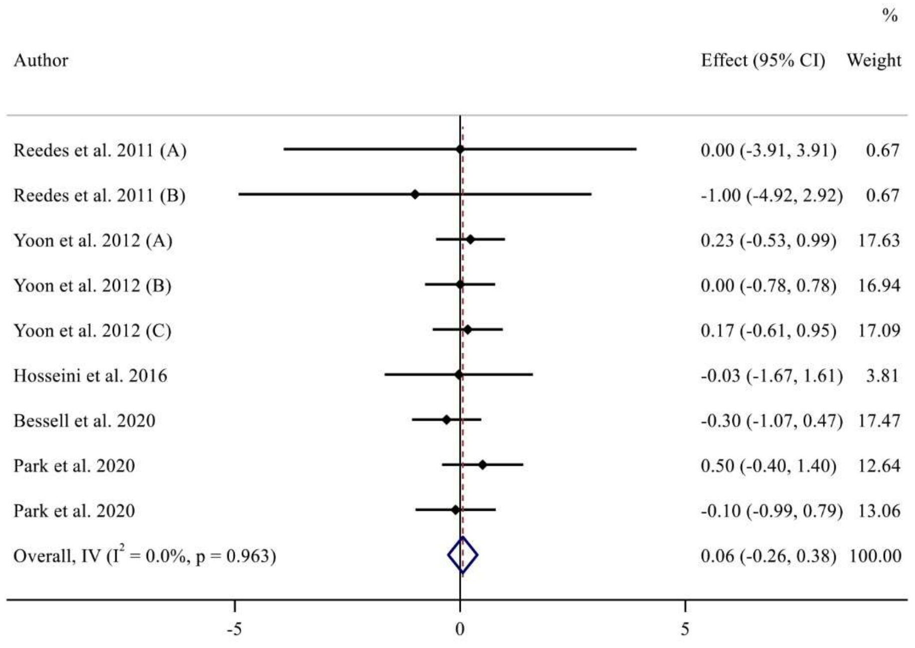

1. c

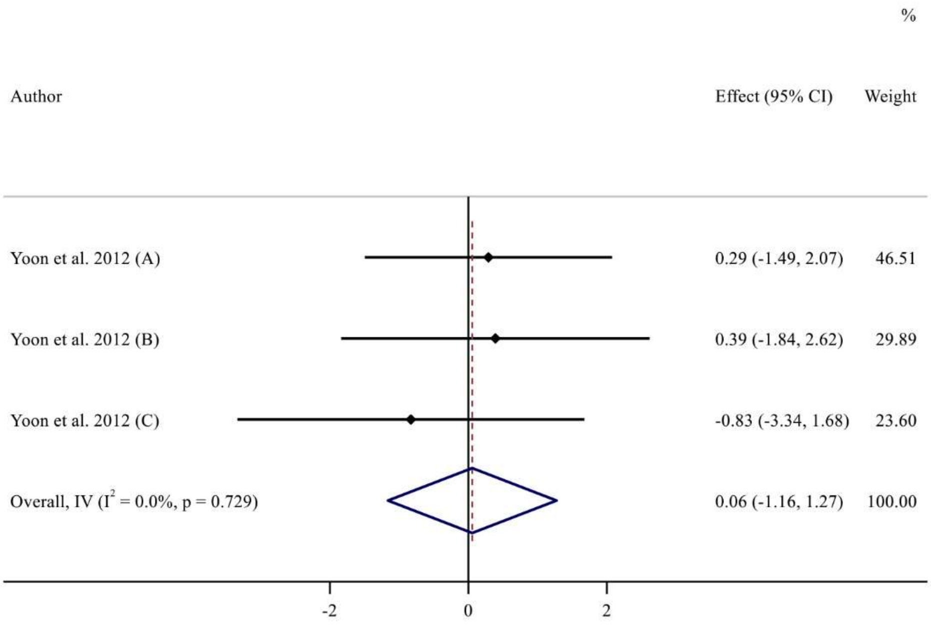
%
Author
Effect (95% CI)
Weight
Yoon et al. 2012 (A)
0.29 (-1.49, 2.07)
46.51
Yoon et al. 2012 (B)
0.39 (-1.84, 2.62)
29.89
Yoon et al. 2012 (C)
-0.83 (-3.34, 1.68)
23.60
Overall, IV (I^2 = 0.0%, p = 0.729)
0.06 (-1.16, 1.27)
100.00
-2
0
2

1. d

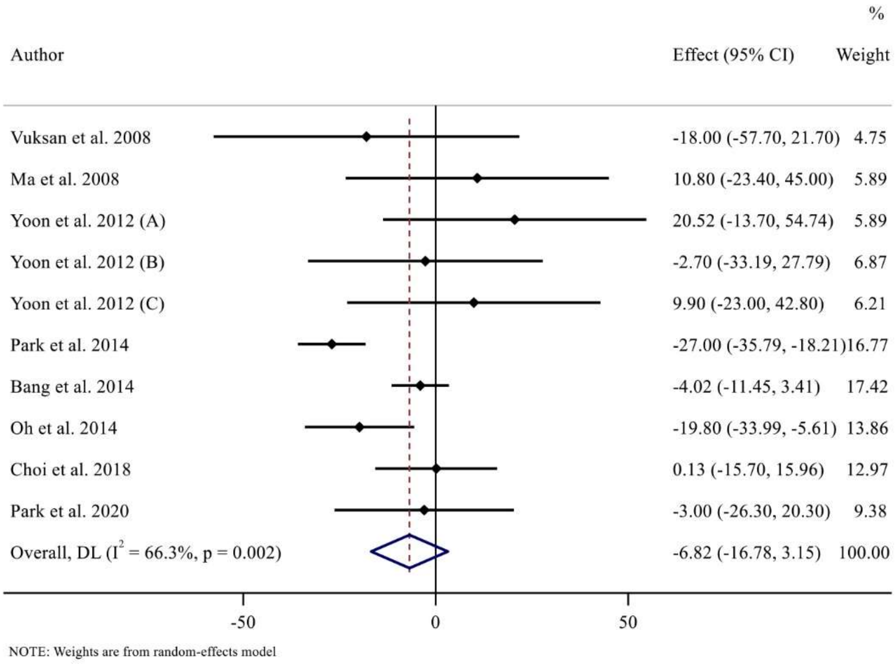
%
Author
Effect (95% CI)
Weight
Vuksan et al. 2008
-18.00 (-57.70, 21.70)
4.75
Ma et al. 2008
10.80 (-23.40, 45.00)
5.89
Yoon et al. 2012 (A)
20.52 (-13.70, 54.74)
5.89
Yoon et al. 2012 (B)
-2.70 (-33.19, 27.79)
6.87
Yoon et al. 2012 (C)
9.90 (-23.00, 42.80)
6.21
Park et al. 2014
-27.00 (-35.79, -18.21)16.77
Bang et al. 2014
-4.02 (-11.45, 3.41)
17.42
Oh et al. 2014
-19.80 (-33.99, -5.61)
13.86
Choi et al. 2018
0.13 (-15.70, 15.96)
12.97
Park et al. 2020
-3.00 (-26.30, 20.30)
9.38
Overall, DL (I^2 = 66.3%, p = 0.002)
-6.82 (-16.78, 3.15)
100.00
-50
0
50
NOTE: Weights are from random-effects model

1. e

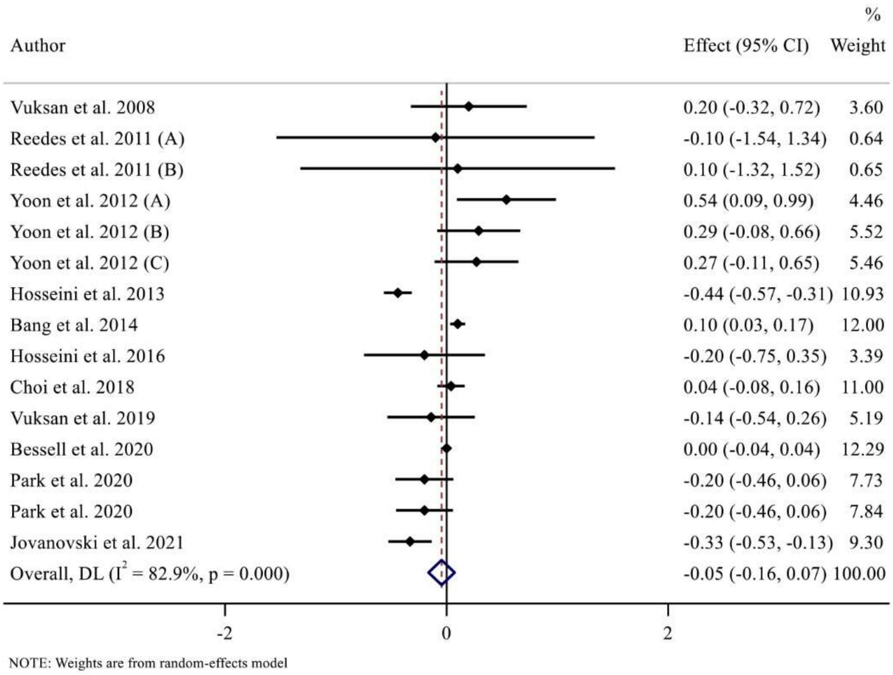

%
Author
Effect (95% CI)
Weight
Vuksan et al. 2008 0.20 (-0.32, 0.72) 3.60
Reedes et al. 2011 (A) -0.10 (-1.54, 1.34) 0.64
Reedes et al. 2011 (B) 0.10 (-1.32, 1.52) 0.65
Yoon et al. 2012 (A) 0.54 (0.09, 0.99) 4.46
Yoon et al. 2012 (B) 0.29 (-0.08, 0.66) 5.52
Yoon et al. 2012 (C) 0.27 (-0.11, 0.65) 5.46
Hosseini et al. 2013 -0.44 (-0.57, -0.31) 10.93
Bang et al. 2014 0.10 (0.03, 0.17) 12.00
Hosseini et al. 2016 -0.20 (-0.75, 0.35) 3.39
Choi et al. 2018 0.04 (-0.08, 0.16) 11.00
Vuksan et al. 2019 -0.14 (-0.54, 0.26) 5.19
Bessell et al. 2020 0.00 (-0.04, 0.04) 12.29
Park et al. 2020 -0.20 (-0.46, 0.06) 7.73
Park et al. 2020 -0.20 (-0.46, 0.06) 7.84
Jovanovski et al. 2021 -0.33 (-0.53, -0.13) 9.30
Overall, DL (I² = 82.9%, p = 0.000) -0.05 (-0.16, 0.07) 100.00
-2
0
2
NOTE: Weights are from random-effects model

1. f

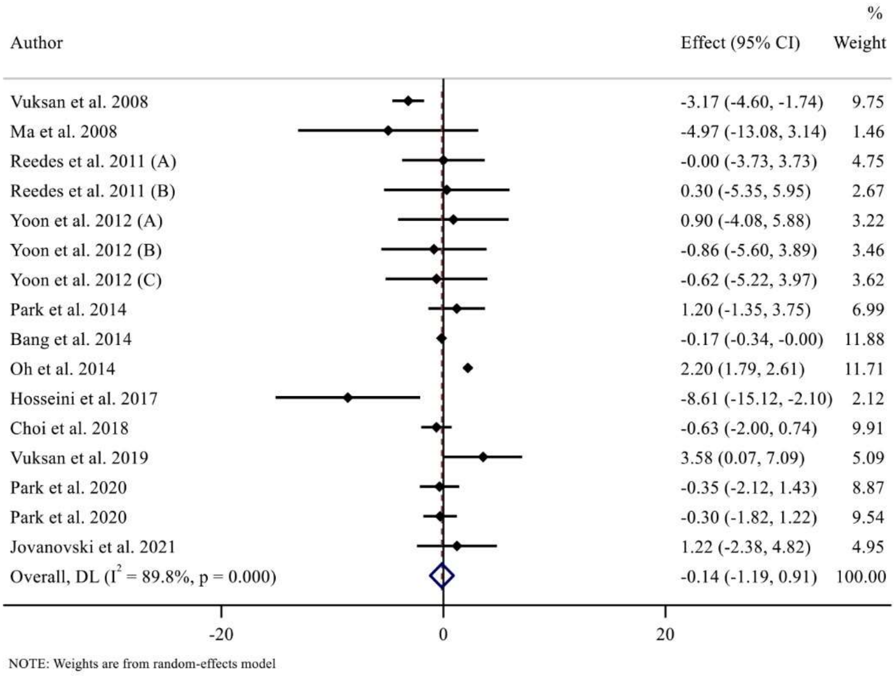

%
Author
Effect (95% CI)
Weight
Vuksan et al. 2008 -3.17 (-4.60, -1.74) 9.75
Ma et al. 2008 -4.97 (-13.08, 3.14) 1.46
Reedes et al. 2011 (A) -0.00 (-3.73, 3.73) 4.75
Reedes et al. 2011 (B) 0.30 (-5.35, 5.95) 2.67
Yoon et al. 2012 (A) 0.90 (-4.08, 5.88) 3.22
Yoon et al. 2012 (B) -0.86 (-5.60, 3.89) 3.46
Yoon et al. 2012 (C) -0.62 (-5.22, 3.97) 3.62
Park et al. 2014 1.20 (-1.35, 3.75) 6.99
Bang et al. 2014 -0.17 (-0.34, -0.00) 11.88
Oh et al. 2014 2.20 (1.79, 2.61) 11.71
Hosseini et al. 2017 -8.61 (-15.12, -2.10) 2.12
Choi et al. 2018 -0.63 (-2.00, 0.74) 9.91
Vuksan et al. 2019 3.58 (0.07, 7.09) 5.09
Park et al. 2020 -0.35 (-2.12, 1.43) 8.87
Park et al. 2020 -0.30 (-1.82, 1.22) 9.54
Jovanovski et al. 2021 1.22 (-2.38, 4.82) 4.95
Overall, DL (I² = 89.8%, p = 0.000) -0.14 (-1.19, 0.91) 100.00
-20
0
20
NOTE: Weights are from random-effects model

1. g

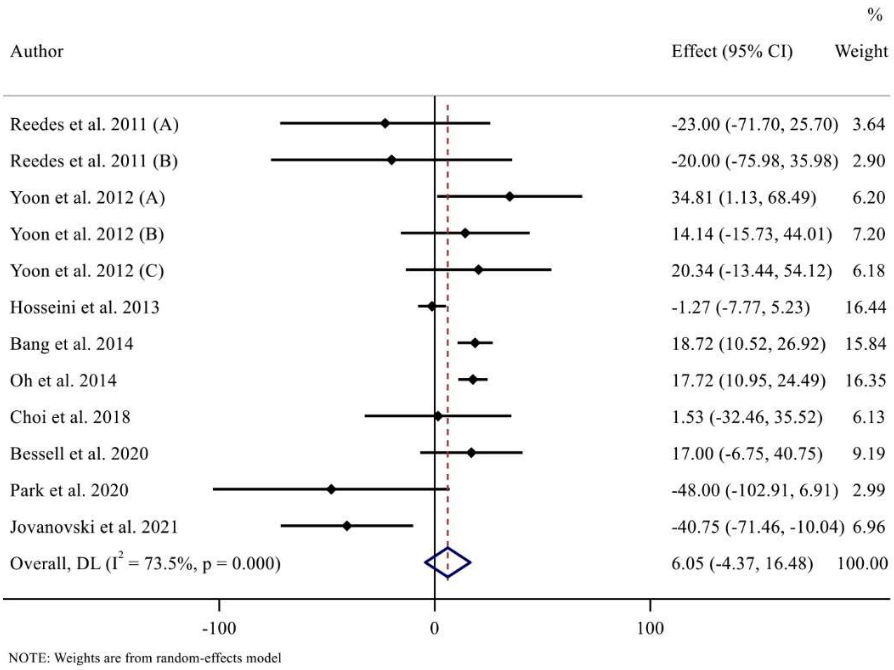
%
Author
Effect (95% CI)
Weight
Reedes et al. 2011 (A) -23.00 (-71.70, 25.70) 3.64
Reedes et al. 2011 (B) -20.00 (-75.98, 35.98) 2.90
Yoon et al. 2012 (A) 34.81 (1.13, 68.49) 6.20
Yoon et al. 2012 (B) 14.14 (-15.73, 44.01) 7.20
Yoon et al. 2012 (C) 20.34 (-13.44, 54.12) 6.18
Hosseini et al. 2013 -1.27 (-7.77, 5.23) 16.44
Bang et al. 2014 18.72 (10.52, 26.92) 15.84
Oh et al. 2014 17.72 (10.95, 24.49) 16.35
Choi et al. 2018 1.53 (-32.46, 35.52) 6.13
Bessell et al. 2020 17.00 (-6.75, 40.75) 9.19
Park et al. 2020 -48.00 (-102.91, 6.91) 2.99
Jovanovski et al. 2021 -40.75 (-71.46, -10.04) 6.96
Overall, DL (I^2 = 73.5%, p = 0.000) 6.05 (-4.37, 16.48) 100.00
-100
0
100
NOTE: Weights are from random-effects model

1. h

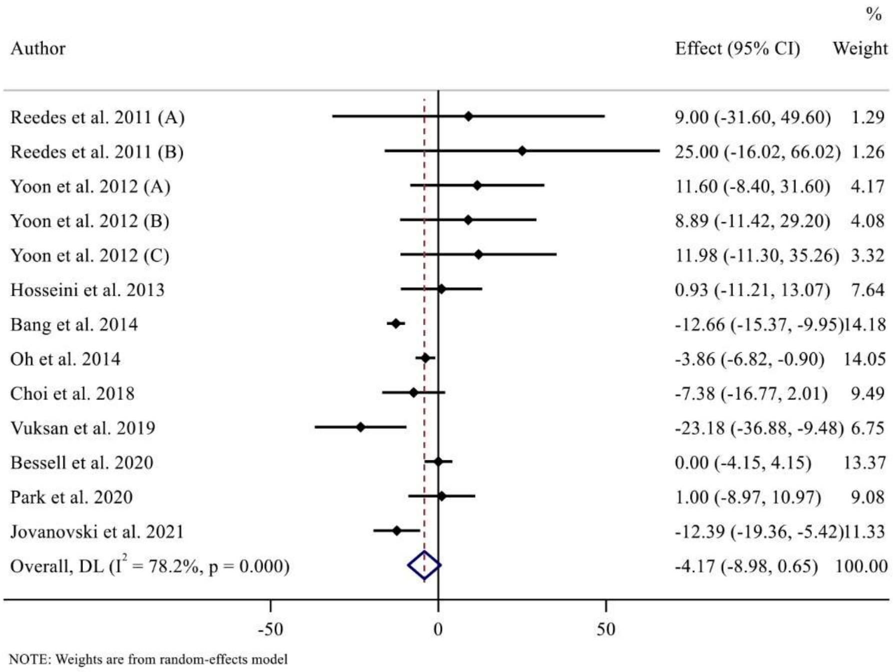
%
Author
Effect (95% CI)
Weight
Reedes et al. 2011 (A) 9.00 (-31.60, 49.60) 1.29
Reedes et al. 2011 (B) 25.00 (-16.02, 66.02) 1.26
Yoon et al. 2012 (A) 11.60 (-8.40, 31.60) 4.17
Yoon et al. 2012 (B) 8.89 (-11.42, 29.20) 4.08
Yoon et al. 2012 (C) 11.98 (-11.30, 35.26) 3.32
Hosseini et al. 2013 0.93 (-11.21, 13.07) 7.64
Bang et al. 2014 -12.66 (-15.37, -9.95)14.18
Oh et al. 2014 -3.86 (-6.82, -0.90) 14.05
Choi et al. 2018 -7.38 (-16.77, 2.01) 9.49
Vuksan et al. 2019 -23.18 (-36.88, -9.48) 6.75
Bessell et al. 2020 0.00 (-4.15, 4.15) 13.37
Park et al. 2020 1.00 (-8.97, 10.97) 9.08
Jovanovski et al. 2021 -12.39 (-19.36, -5.42)11.33
Overall, DL (I^2 = 78.2%, p = 0.000) -4.17 (-8.98, 0.65) 100.00
-50
0
50
NOTE: Weights are from random-effects model

1. i

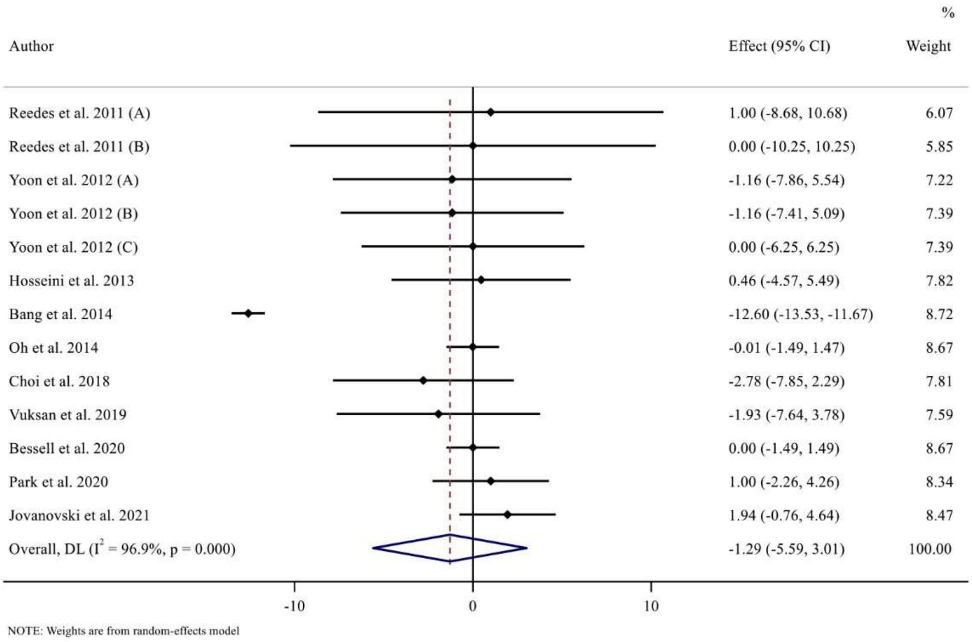

%
Author
Effect (95% CI)
Weight
Reedes et al. 2011 (A) 1.00 (-8.68, 10.68) 6.07
Reedes et al. 2011 (B) 0.00 (-10.25, 10.25) 5.85
Yoon et al. 2012 (A) -1.16 (-7.86, 5.54) 7.22
Yoon et al. 2012 (B) -1.16 (-7.41, 5.09) 7.39
Yoon et al. 2012 (C) 0.00 (-6.25, 6.25) 7.39
Hosseini et al. 2013 0.46 (-4.57, 5.49) 7.82
Bang et al. 2014 -12.60 (-13.53, -11.67) 8.72
Oh et al. 2014 -0.01 (-1.49, 1.47) 8.67
Choi et al. 2018 -2.78 (-7.85, 2.29) 7.81
Vuksan et al. 2019 -1.93 (-7.64, 3.78) 7.59
Bessell et al. 2020 0.00 (-1.49, 1.49) 8.67
Park et al. 2020 1.00 (-2.26, 4.26) 8.34
Jovanovski et al. 2021 1.94 (-0.76, 4.64) 8.47
Overall, DL (I2 = 96.9%, p = 0.000) -1.29 (-5.59, 3.01) 100.00
-10
0
10
NOTE: Weights are from random-effects model

1. j

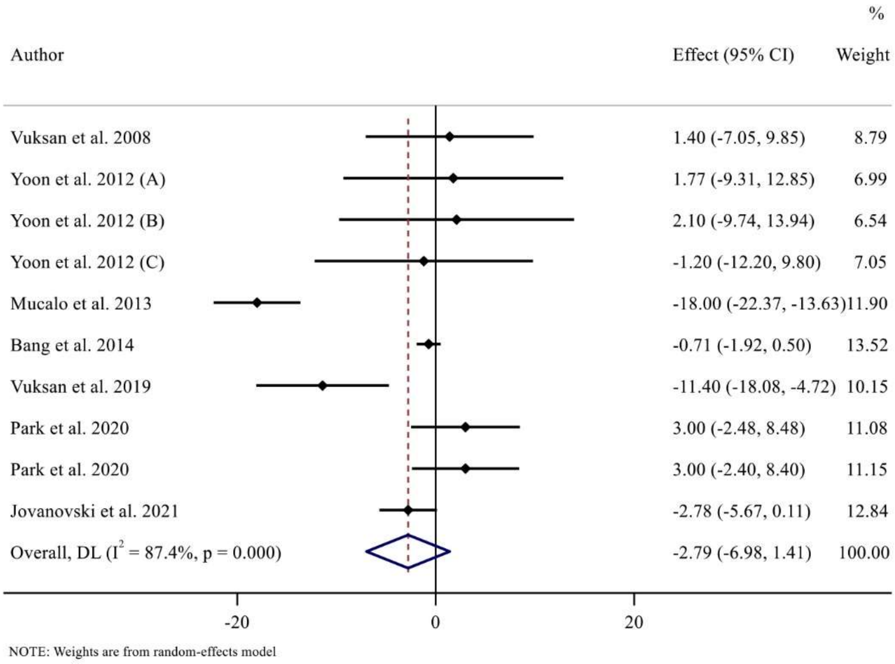

%
Author
Effect (95% CI)
Weight
Vuksan et al. 2008 1.40 (-7.05, 9.85) 8.79
Yoon et al. 2012 (A) 1.77 (-9.31, 12.85) 6.99
Yoon et al. 2012 (B) 2.10 (-9.74, 13.94) 6.54
Yoon et al. 2012 (C) -1.20 (-12.20, 9.80) 7.05
Mucalo et al. 2013 -18.00 (-22.37, -13.63)11.90
Bang et al. 2014 -0.71 (-1.92, 0.50) 13.52
Vuksan et al. 2019 -11.40 (-18.08, -4.72) 10.15
Park et al. 2020 3.00 (-2.48, 8.48) 11.08
Park et al. 2020 3.00 (-2.40, 8.40) 11.15
Jovanovski et al. 2021 -2.78 (-5.67, 0.11) 12.84
Overall, DL (I2 = 87.4%, p = 0.000) -2.79 (-6.98, 1.41) 100.00
-20
0
20
NOTE: Weights are from random-effects model

1. k

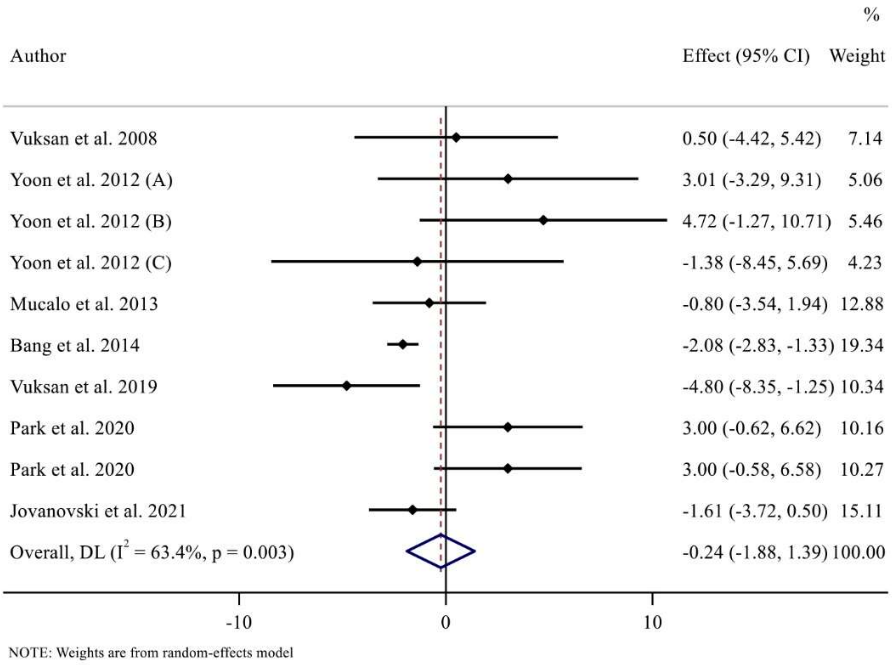
%
Author
Effect (95% CI)
Weight
Vuksan et al. 2008
0.50 (-4.42, 5.42)
7.14
Yoon et al. 2012 (A)
3.01 (-3.29, 9.31)
5.06
Yoon et al. 2012 (B)
4.72 (-1.27, 10.71)
5.46
Yoon et al. 2012 (C)
-1.38 (-8.45, 5.69)
4.23
Mucalo et al. 2013
-0.80 (-3.54, 1.94)
12.88
Bang et al. 2014
-2.08 (-2.83, -1.33)
19.34
Vuksan et al. 2019
-4.80 (-8.35, -1.25)
10.34
Park et al. 2020
3.00 (-0.62, 6.62)
10.16
Park et al. 2020
3.00 (-0.58, 6.58)
10.27
Jovanovski et al. 2021
-1.61 (-3.72, 0.50)
15.11
Overall, DL (I^2 = 63.4%, p = 0.003)
-0.24 (-1.88, 1.39)
100.00
-10
0
10
NOTE: Weights are from random-effects model

1. l

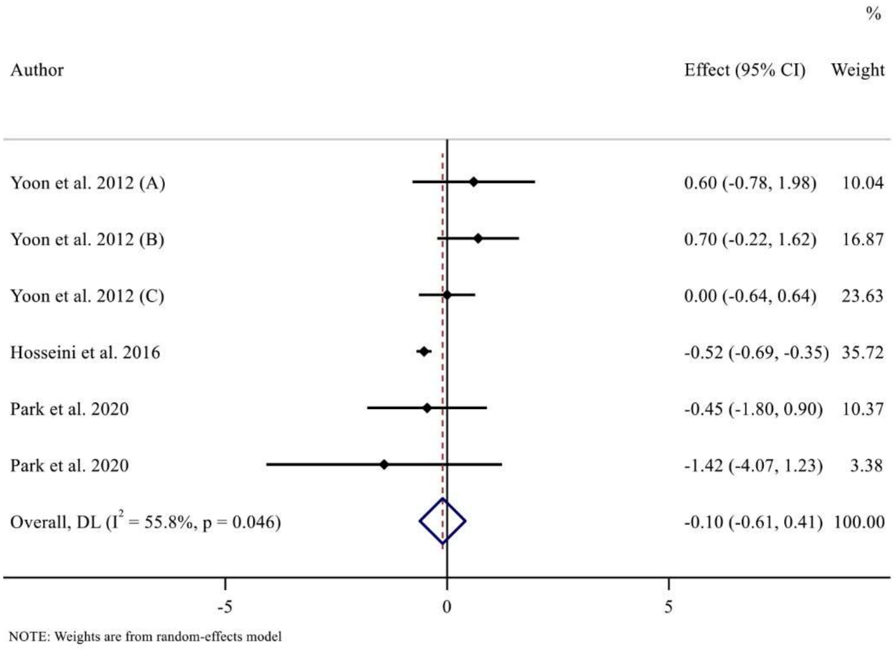
%
Author
Effect (95% CI)
Weight
Yoon et al. 2012 (A)
0.60 (-0.78, 1.98)
10.04
Yoon et al. 2012 (B)
0.70 (-0.22, 1.62)
16.87
Yoon et al. 2012 (C)
0.00 (-0.64, 0.64)
23.63
Hosseini et al. 2016
-0.52 (-0.69, -0.35)
35.72
Park et al. 2020
-0.45 (-1.80, 0.90)
10.37
Park et al. 2020
-1.42 (-4.07, 1.23)
3.38
Overall, DL (I^2 = 55.8%, p = 0.046)
-0.10 (-0.61, 0.41)
100.00
-5
0
5
NOTE: Weights are from random-effects model

1. m

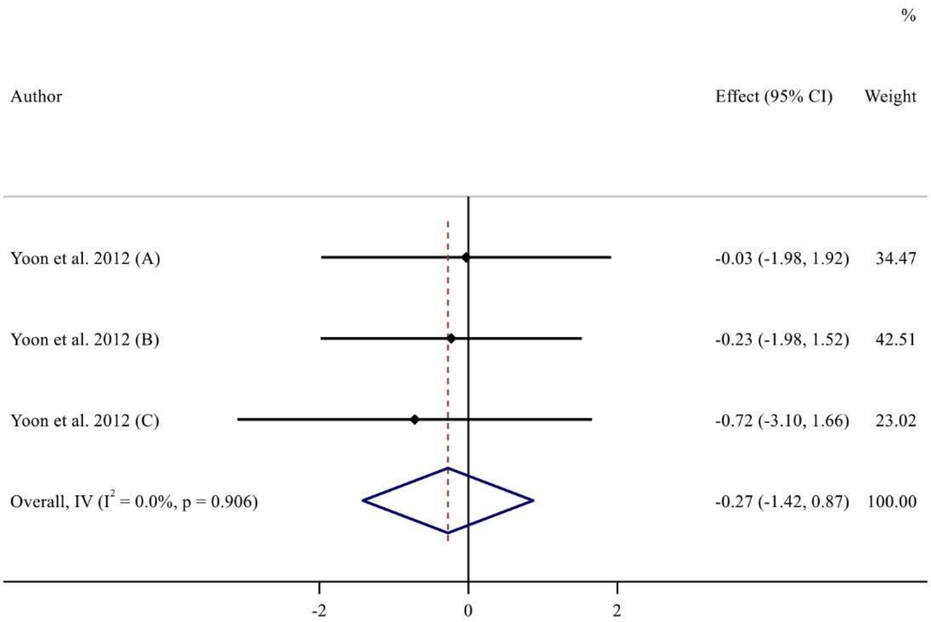

%
Author
Effect (95% CI)
Weight
Yoon et al. 2012 (A)
-0.03 (-1.98, 1.92)
34.47
Yoon et al. 2012 (B)
-0.23 (-1.98, 1.52)
42.51
Yoon et al. 2012 (C)
-0.72 (-3.10, 1.66)
23.02
Overall, IV (I^2 = 0.0%, p = 0.906)
-0.27 (-1.42, 0.87)
100.00
-2
0
2

1. n

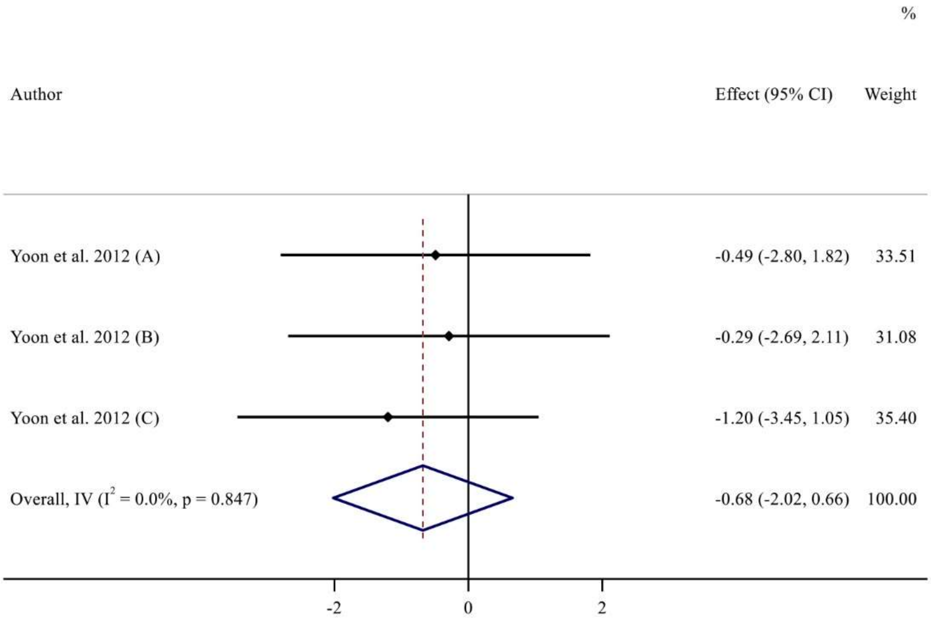

%
Author
Effect (95% CI)
Weight
Yoon et al. 2012 (A)
-0.49 (-2.80, 1.82)
33.51
Yoon et al. 2012 (B)
-0.29 (-2.69, 2.11)
31.08
Yoon et al. 2012 (C)
-1.20 (-3.45, 1.05)
35.40
Overall, IV (I^2 = 0.0%, p = 0.847)
-0.68 (-2.02, 0.66)
100.00
-2
0
2

1. o

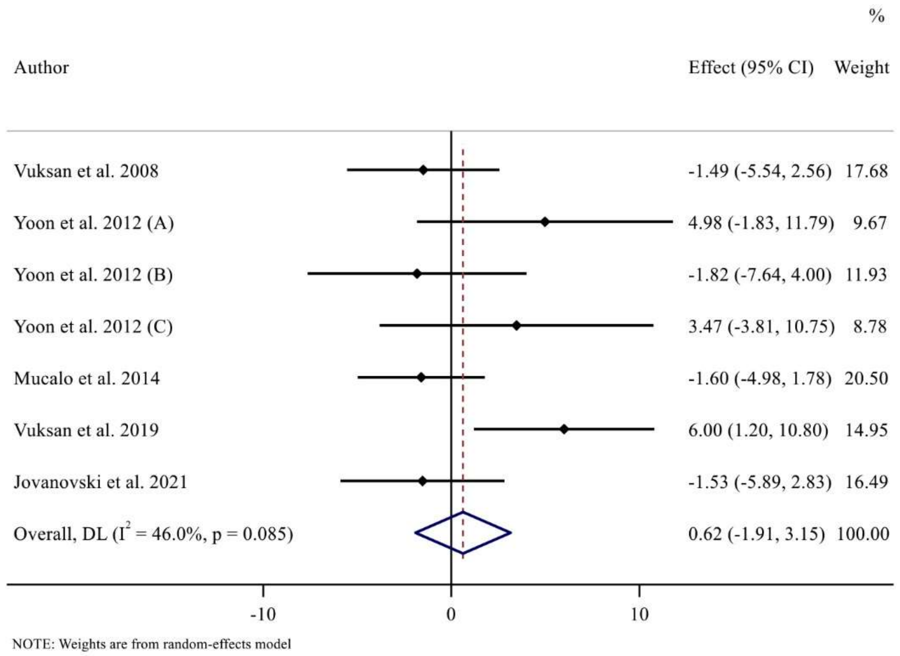
%
Author
Effect (95% CI)
Weight
Vuksan et al. 2008
-1.49 (-5.54, 2.56)
17.68
Yoon et al. 2012 (A)
4.98 (-1.83, 11.79)
9.67
Yoon et al. 2012 (B)
-1.82 (-7.64, 4.00)
11.93
Yoon et al. 2012 (C)
3.47 (-3.81, 10.75)
8.78
Mucalo et al. 2014
-1.60 (-4.98, 1.78)
20.50
Vuksan et al. 2019
6.00 (1.20, 10.80)
14.95
Jovanovski et al. 2021
-1.53 (-5.89, 2.83)
16.49
Overall, DL (I² = 46.0%, p = 0.085)
0.62 (-1.91, 3.15)
100.00
-10
0
10
NOTE: Weights are from random-effects model

1. p

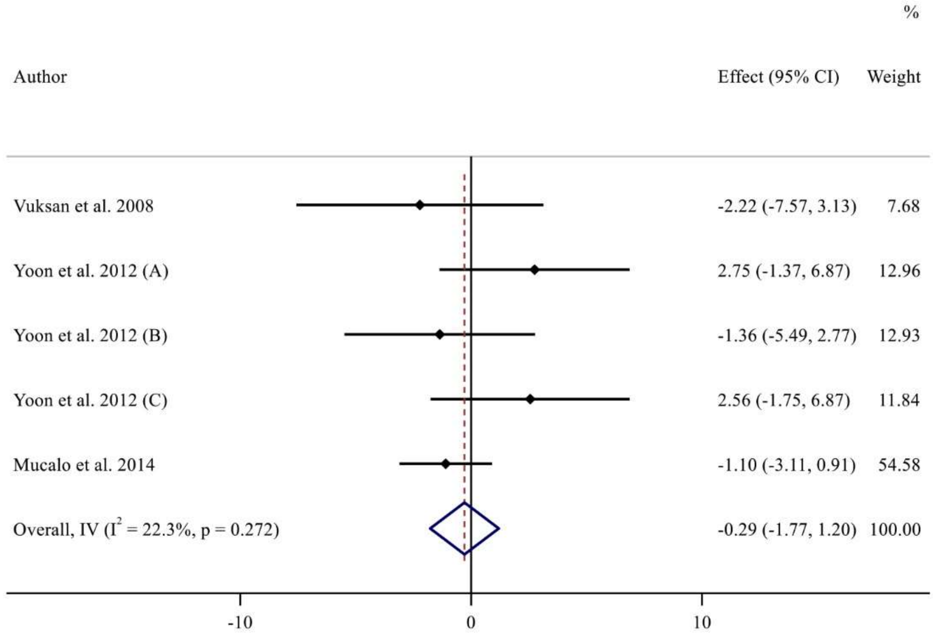
%
Author
Effect (95% CI)
Weight
Vuksan et al. 2008
-2.22 (-7.57, 3.13)
7.68
Yoon et al. 2012 (A)
2.75 (-1.37, 6.87)
12.96
Yoon et al. 2012 (B)
-1.36 (-5.49, 2.77)
12.93
Yoon et al. 2012 (C)
2.56 (-1.75, 6.87)
11.84
Mucalo et al. 2014
-1.10 (-3.11, 0.91)
54.58
Overall, IV (I² = 22.3%, p = 0.272)
-0.29 (-1.77, 1.20)
100.00
-10
0
10

1. q

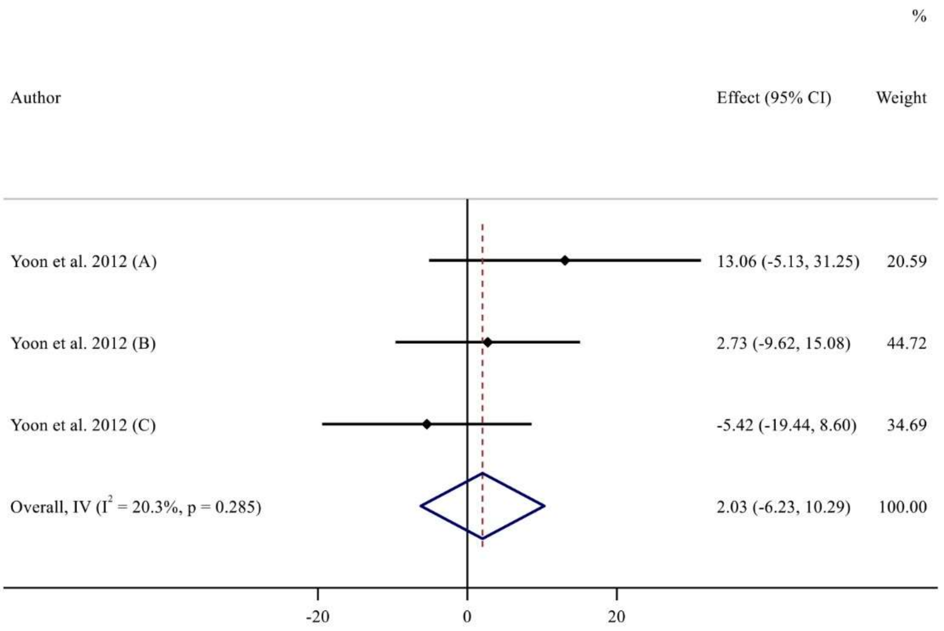

a: body weight (BW); b: body mass index (BMI); c: waist circumference (WC); d: oral glucose tolerance test (OGTT); e: hemoglobin A1c (HbA1c); f: fasting insulin; g: triglyceride (TG); h: low-density lipoprotein cholesterol (LDL-C); i: high-density lipoprotein cholesterol (HDL-C); j: systolic blood pressure (SBP); k: diastolic blood pressure (DBP); l: C-reactive protein (CRP); m: adiponectin; n: leptin; o: alanine aminotransferase (ALT); p: aspartate aminotransferase (AST); q: gamma-glutamyl transferase (GGT).

Supplementary Figure S2. Funnel plots demonstrating publication bias in the trials reporting the effect of ginseng supplementation.

2. a

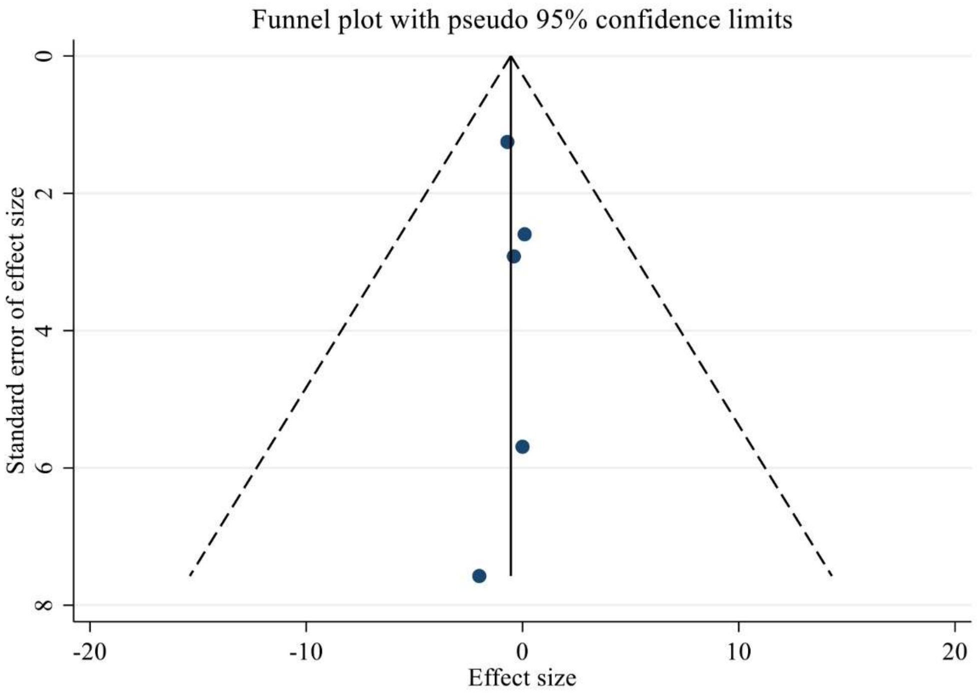

2. b

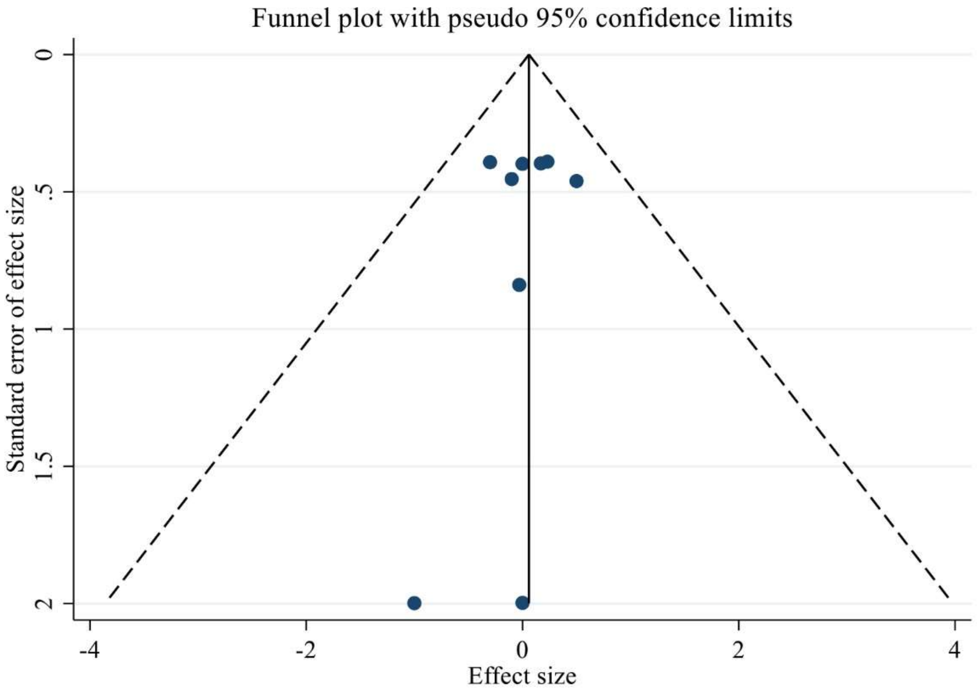

2. c

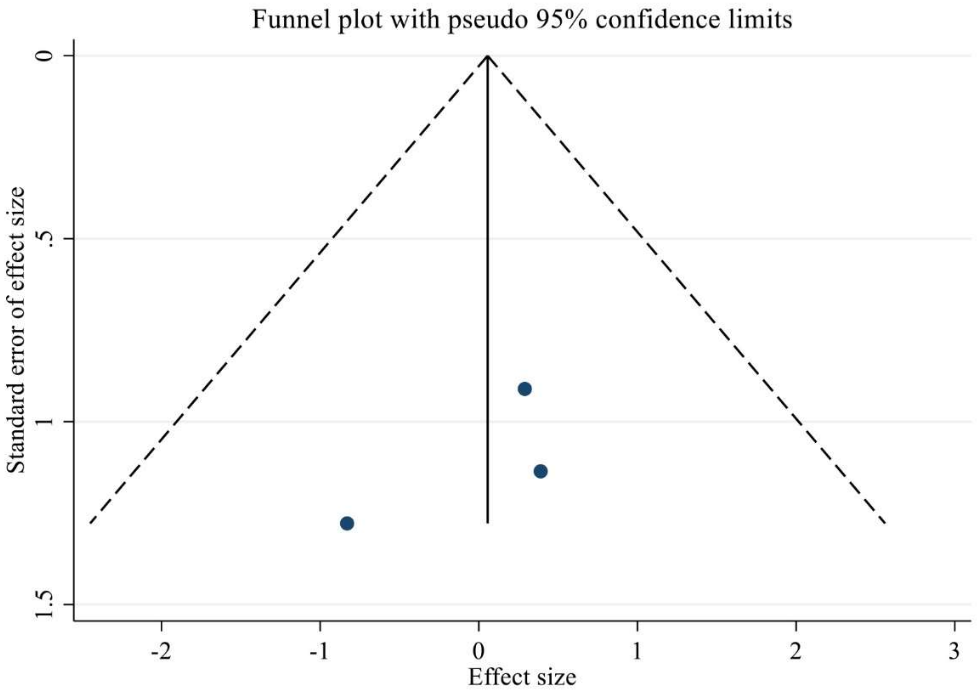
Funnel plot with pseudo 95% confidence limits
Standard error of effect size
0
.5
1
1.5
-2
-1
0
1
2
3
Effect size

2. d

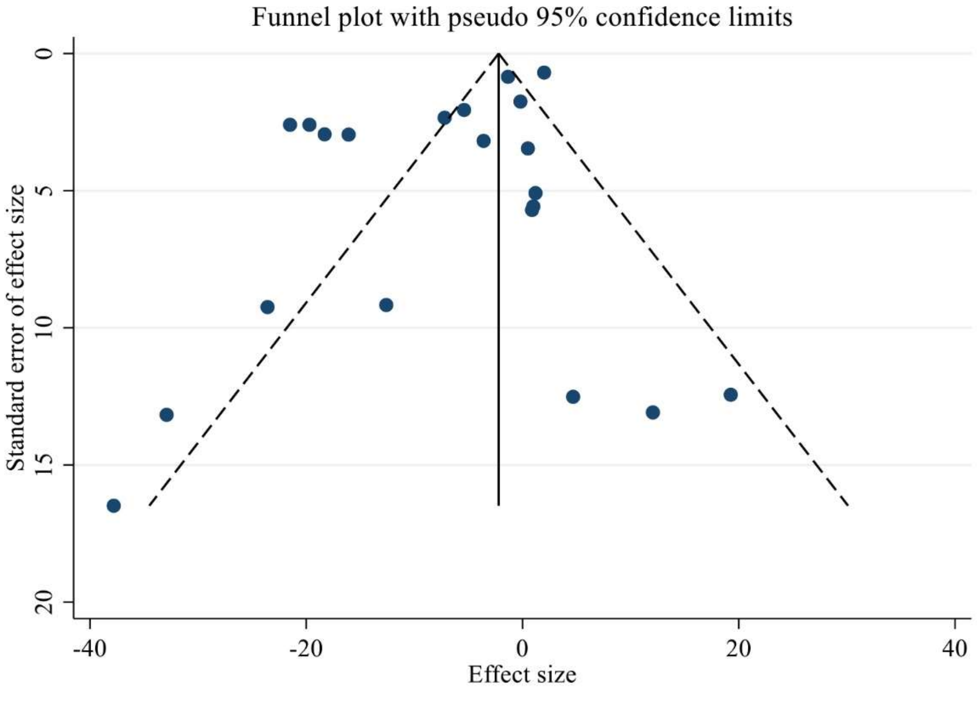
Funnel plot with pseudo 95% confidence limits
Standard error of effect size
0
5
10
15
20
-40
-20
0
20
40
Effect size

2. e

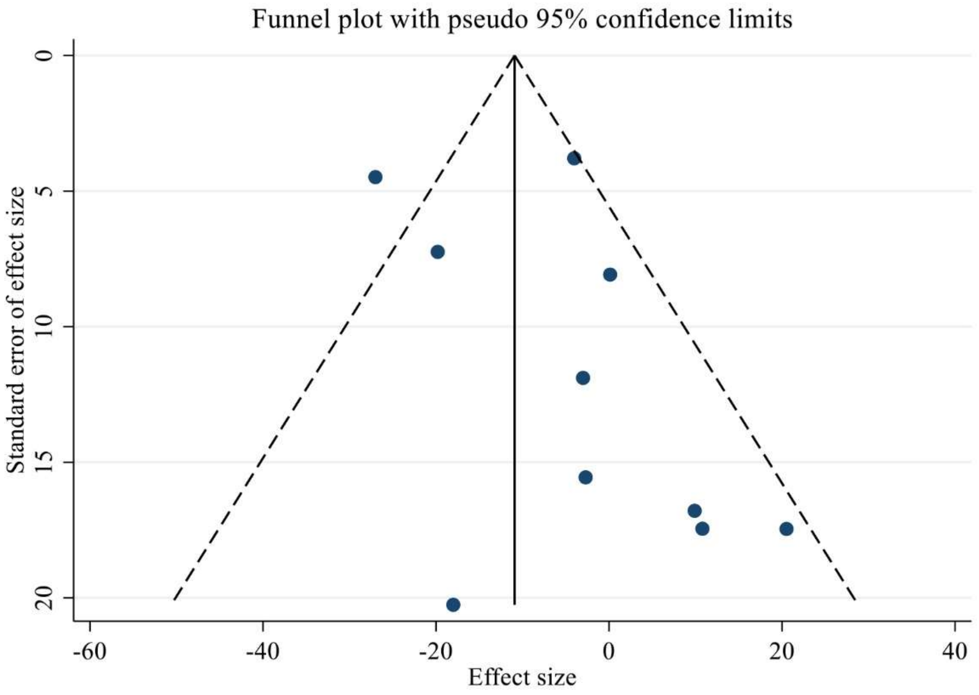
Funnel plot with pseudo 95% confidence limits
Standard error of effect size
0
5
10
15
20
-60
-40
-20
0
20
40
Effect size

2. f

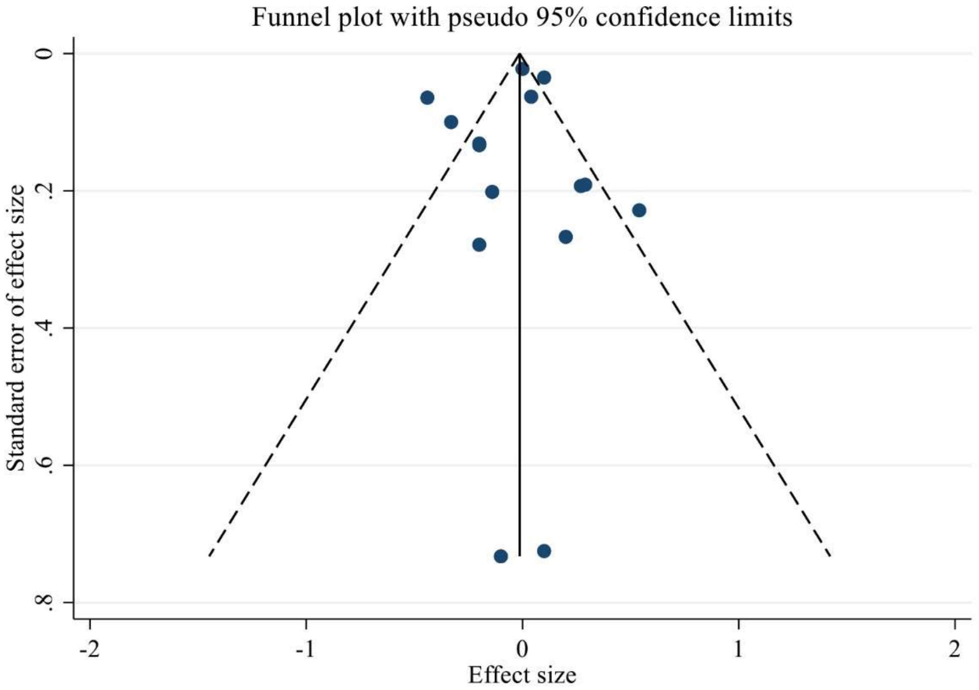
Funnel plot with pseudo 95% confidence limits
Standard error of effect size
0
.2
.4
.6
.8
-2
-1
0
1
2
Effect size

2. g

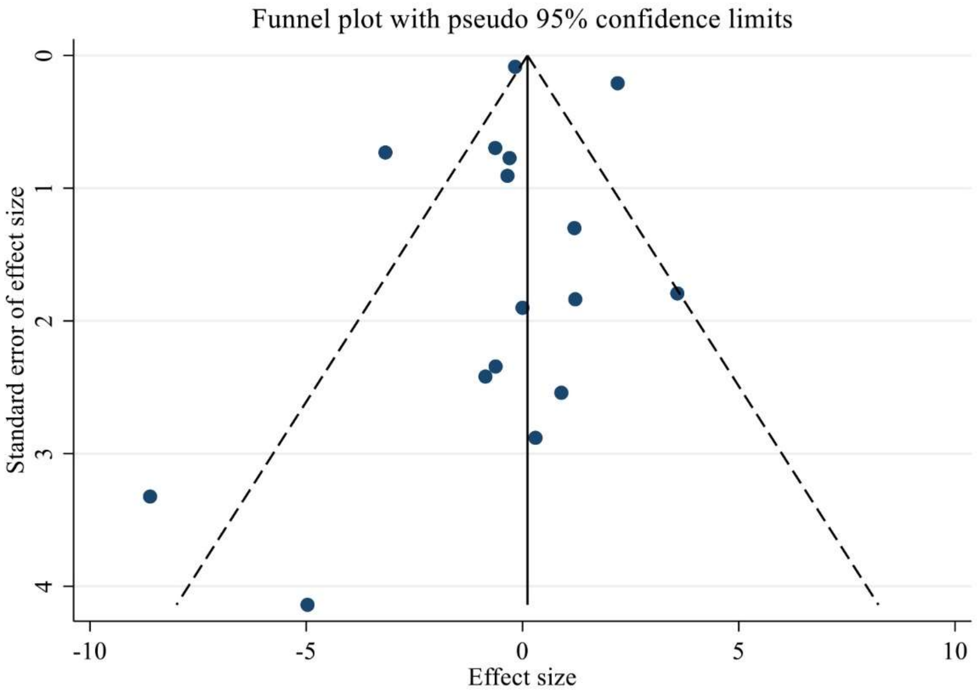
Funnel plot with pseudo 95% confidence limits
Standard error of effect size
0
1
2
3
4
-10
-5
0
5
10
Effect size

2. h

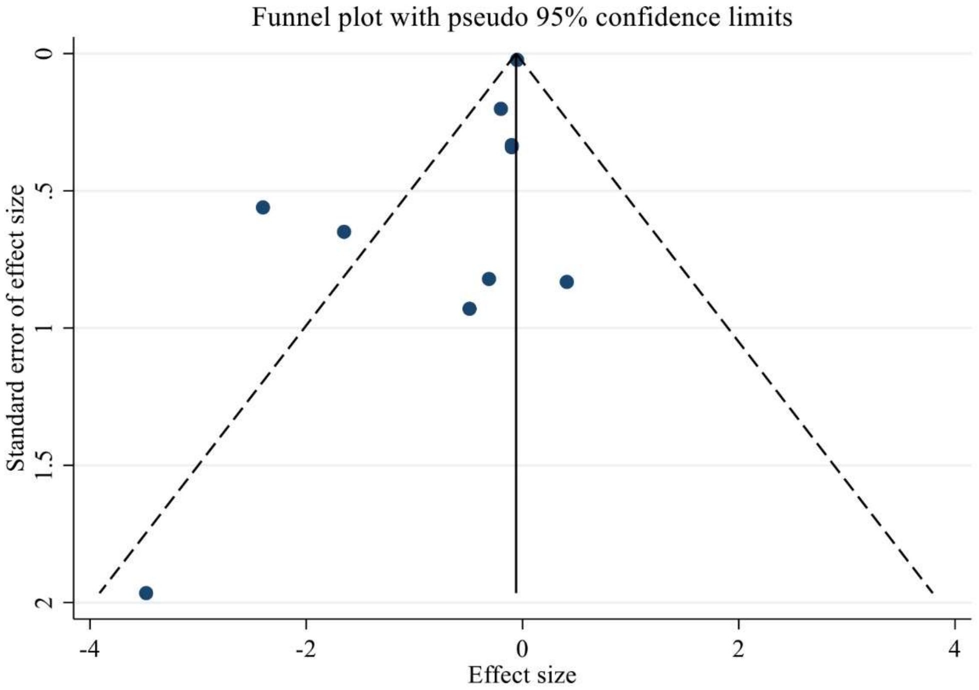
Funnel plot with pseudo 95% confidence limits
Standard error of effect size
0
.5
1
1.5
2
-4
-2
0
2
4
Effect size

2. i

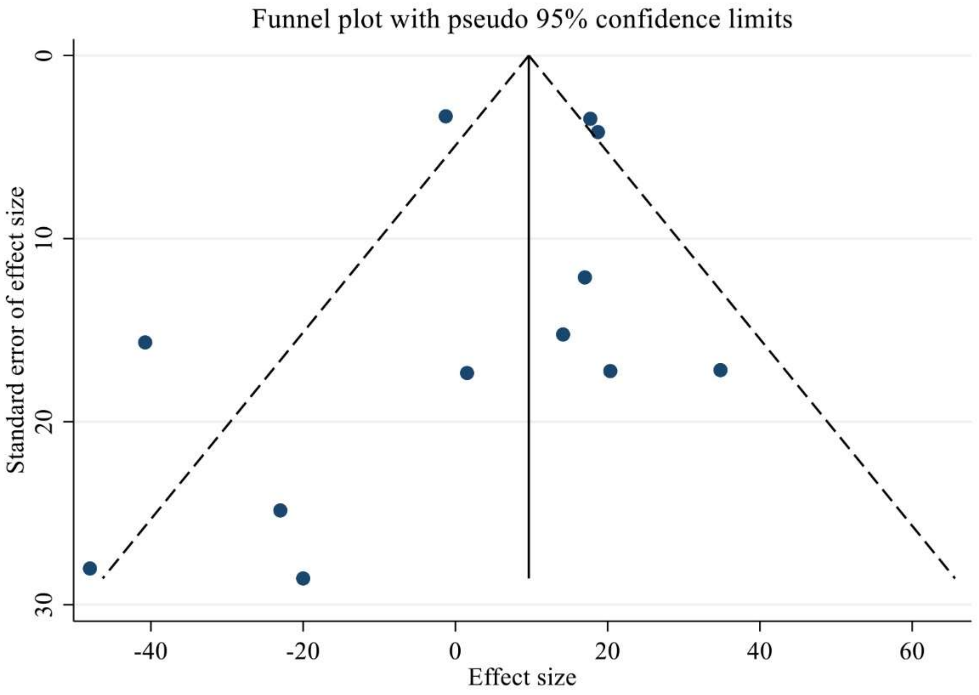
Funnel plot with pseudo 95% confidence limits
Standard error of effect size
0
10
20
30
-40
-20
0
20
40
60
Effect size

2. j

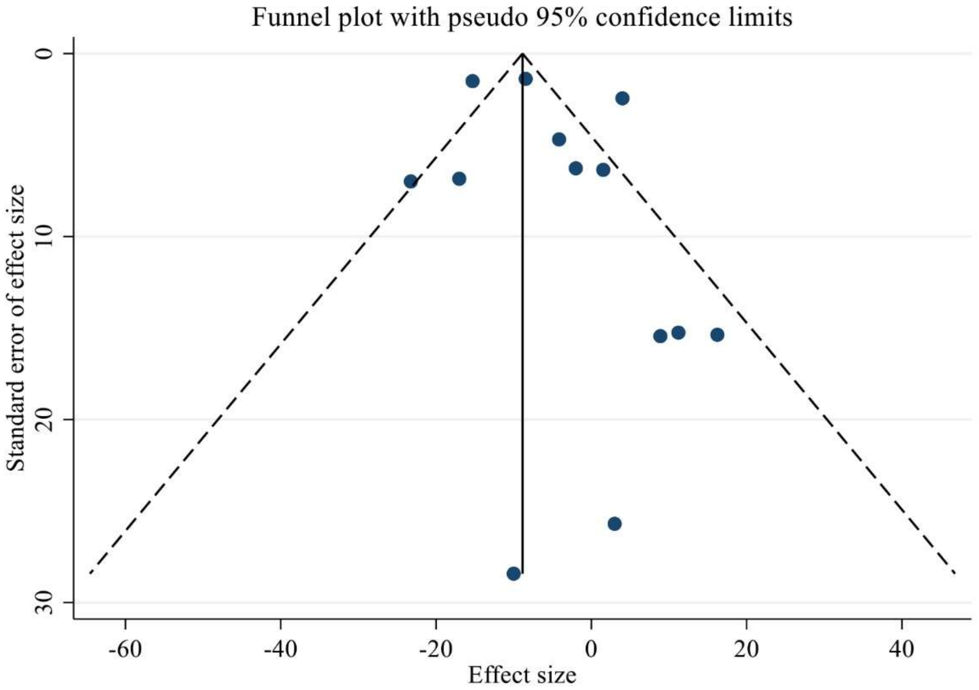
Funnel plot with pseudo 95% confidence limits
Standard error of effect size
0
10
20
30
-60
-40
-20
0
20
40
Effect size

2. k

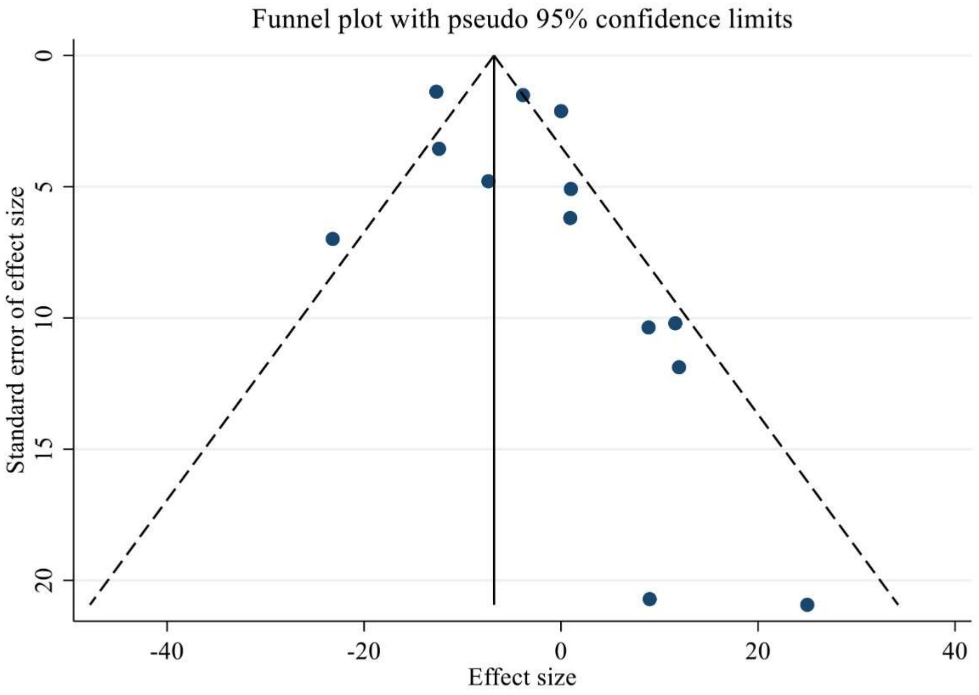

Funnel plot with pseudo 95% confidence limits
Standard error of effect size
0
5
10
15
20
-40
-20
0
20
40
Effect size

2. l

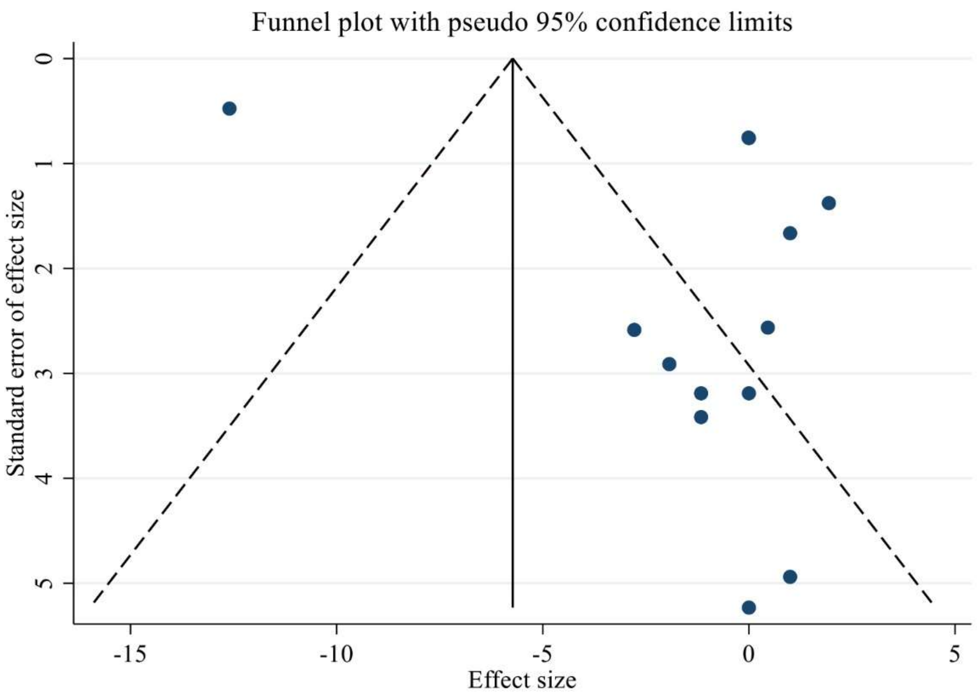

Funnel plot with pseudo 95% confidence limits
Standard error of effect size
0
1
2
3
4
5
-15
-10
-5
0
5
Effect size

2. m

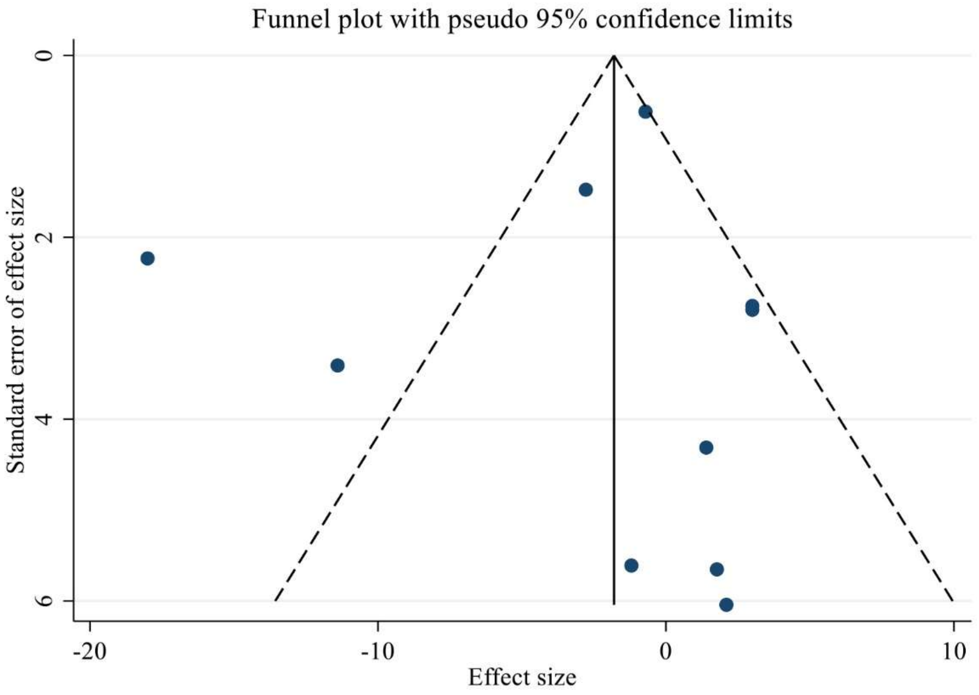
Funnel plot with pseudo 95% confidence limits
Standard error of effect size
0
2
4
6
-20
-10
0
10
Effect size

2. n

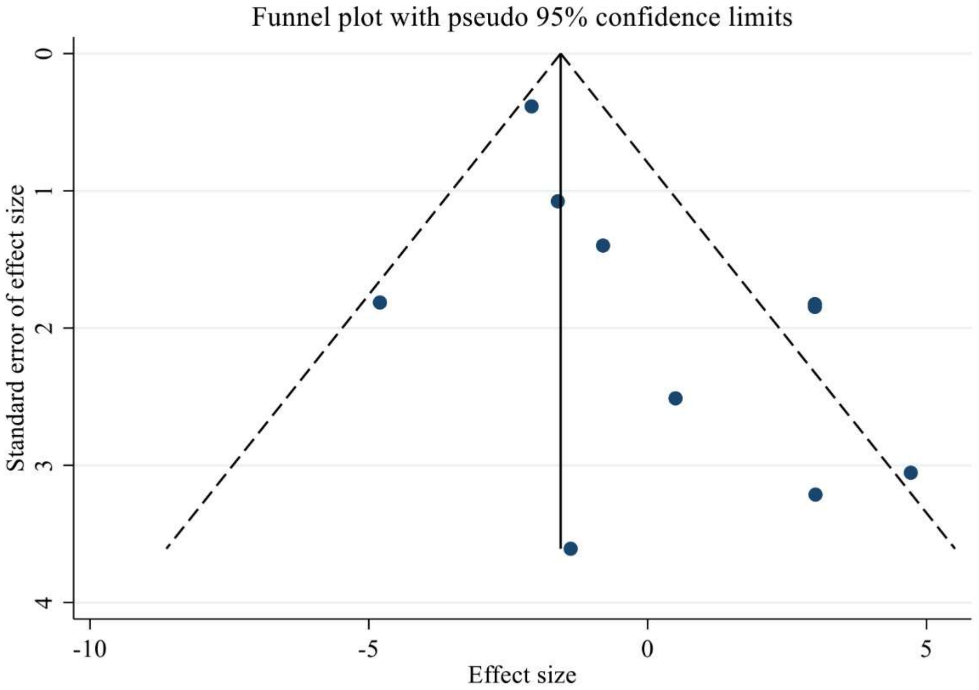
Funnel plot with pseudo 95% confidence limits
Standard error of effect size
0
1
2
3
4
-10
-5
0
5
Effect size

2. o

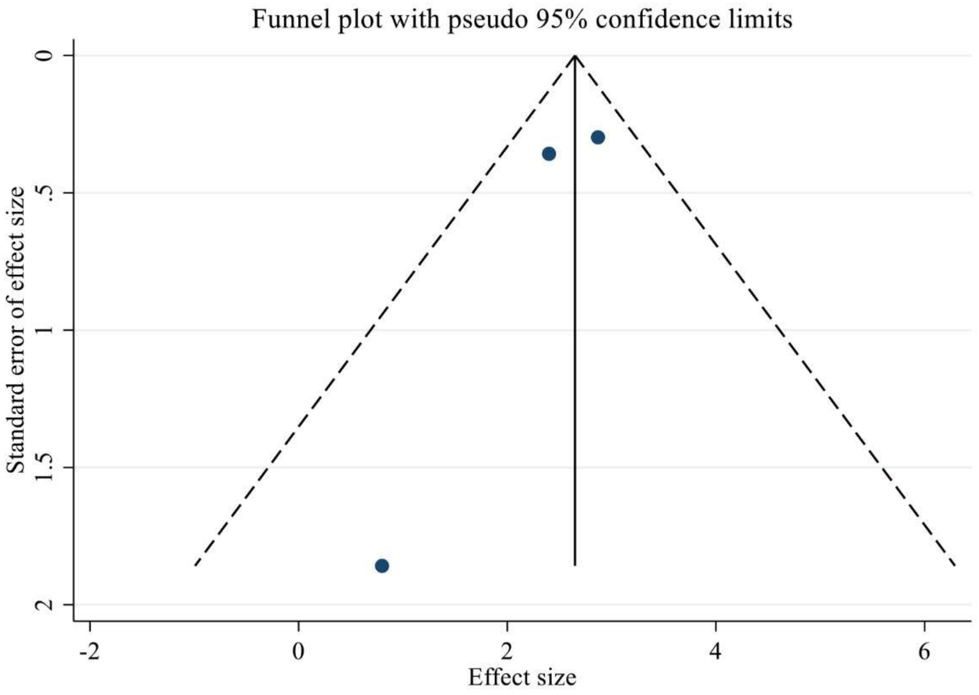
Funnel plot with pseudo 95% confidence limits
Standard error of effect size
0
.5
1
1.5
2
-2
0
2
4
6
Effect size

2. p

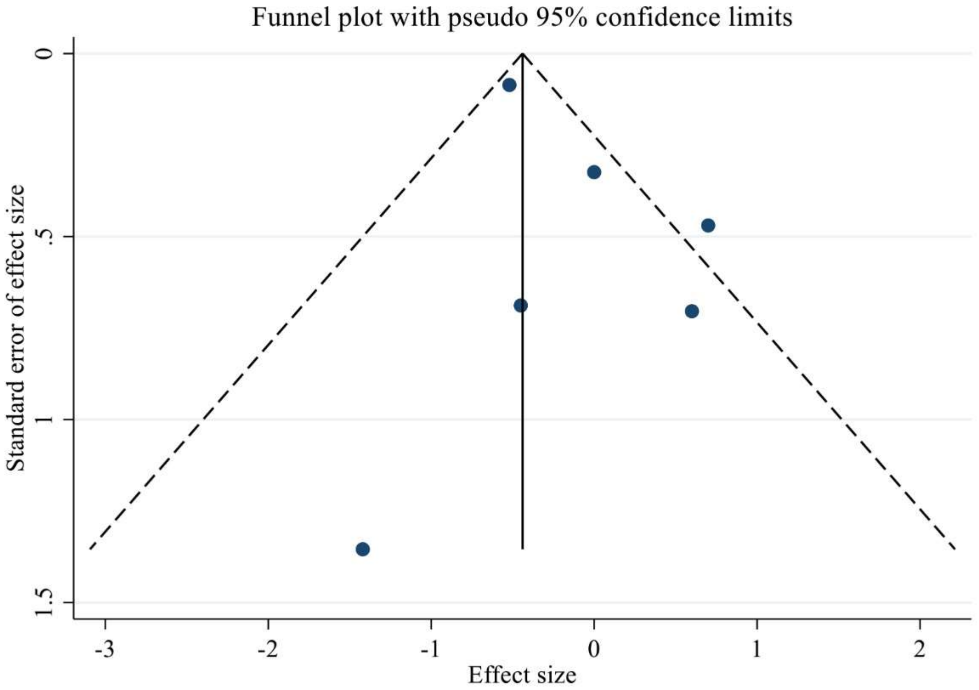
Funnel plot with pseudo 95% confidence limits
Standard error of effect size
0
.5
1
1.5
-3
-2
-1
0
1
2
Effect size

2. q

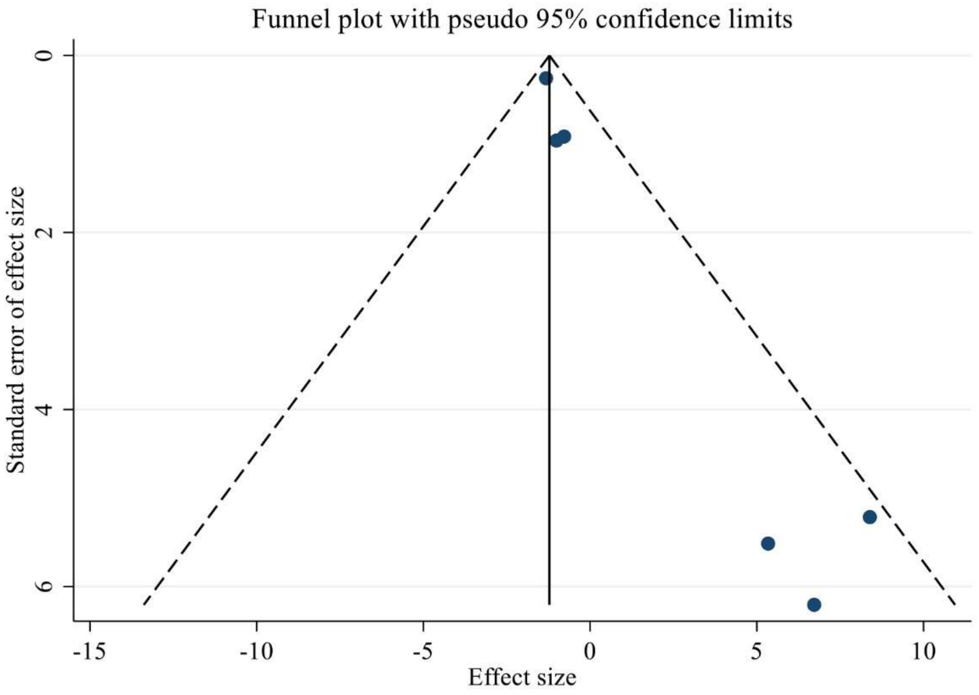

Funnel plot with pseudo 95% confidence limits
Standard error of effect size
0
2
4
6
-15
-10
-5
0
5
10
Effect size

2. r

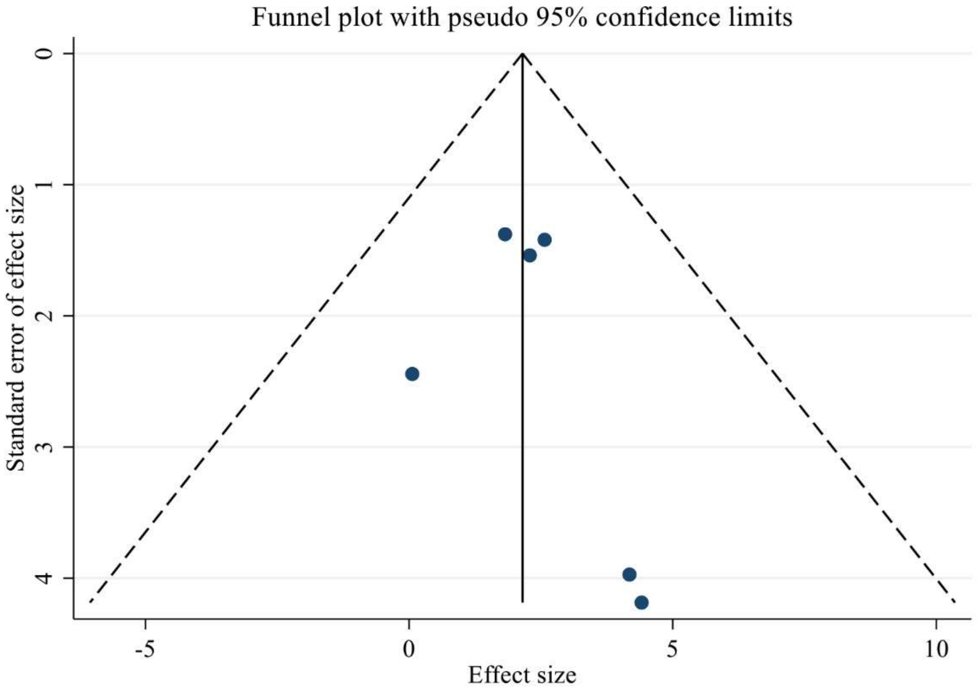

Funnel plot with pseudo 95% confidence limits
Standard error of effect size
0
1
2
3
4
-5
0
5
10
Effect size

2. s

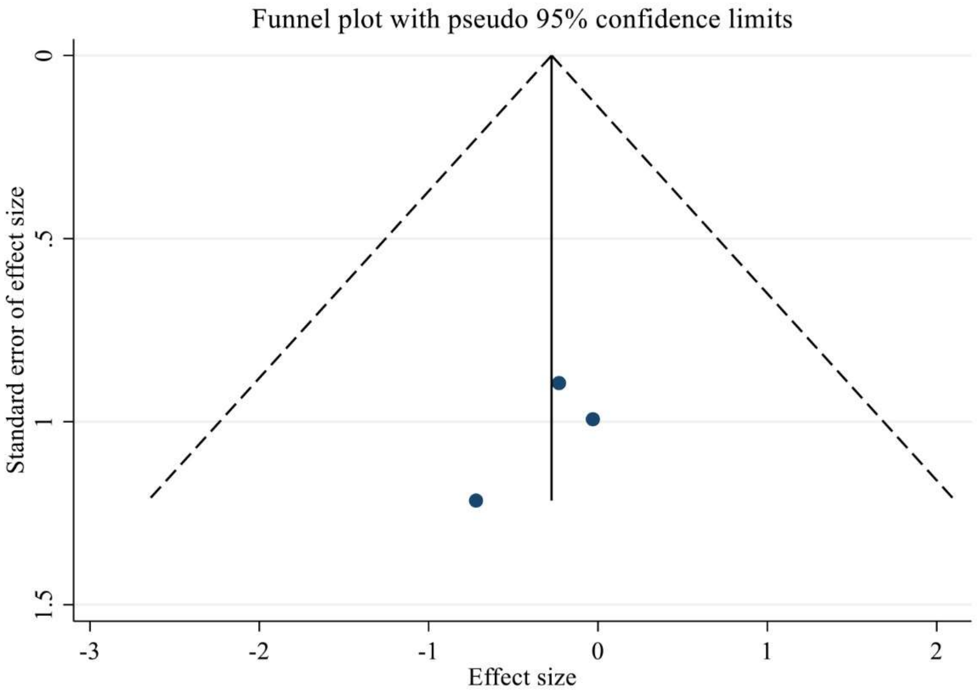
Funnel plot with pseudo 95% confidence limits
Standard error of effect size
0
.5
1
1.5
-3
-2
-1
0
1
2
Effect size

2. t

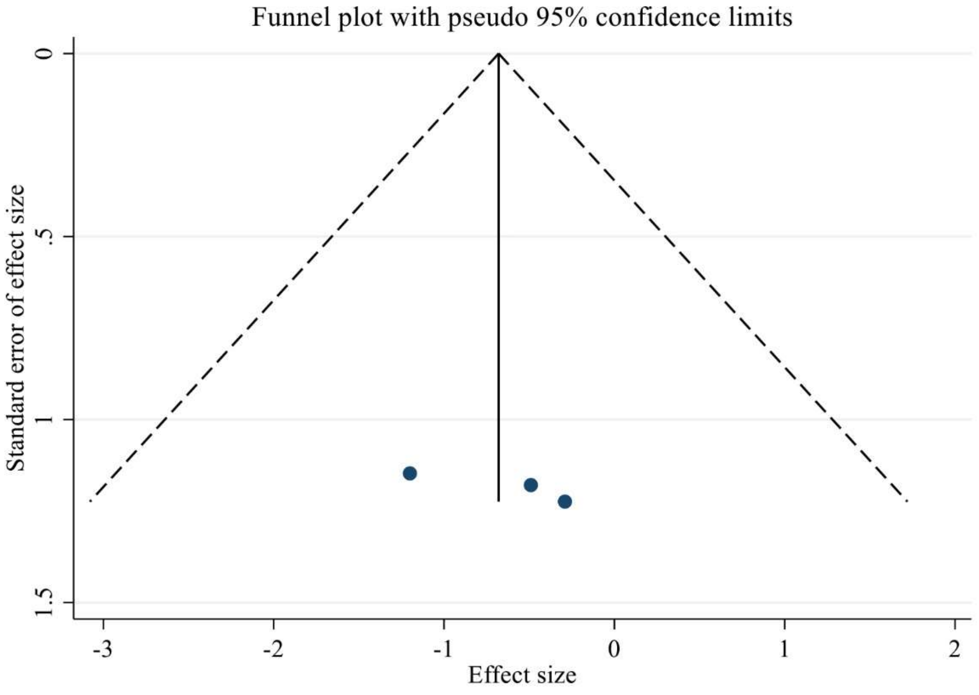
Funnel plot with pseudo 95% confidence limits
Standard error of effect size
0
.5
1
1.5
-3
-2
-1
0
1
2
Effect size

2. u

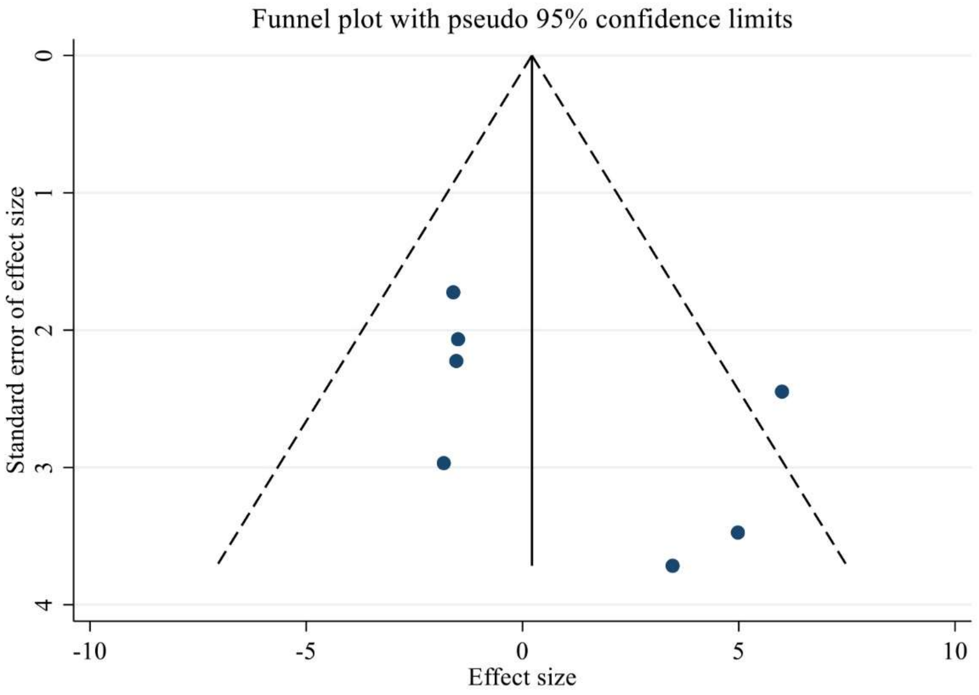
Funnel plot with pseudo 95% confidence limits
Standard error of effect size
0
1
2
3
4
-10
-5
0
5
10
Effect size

2. v

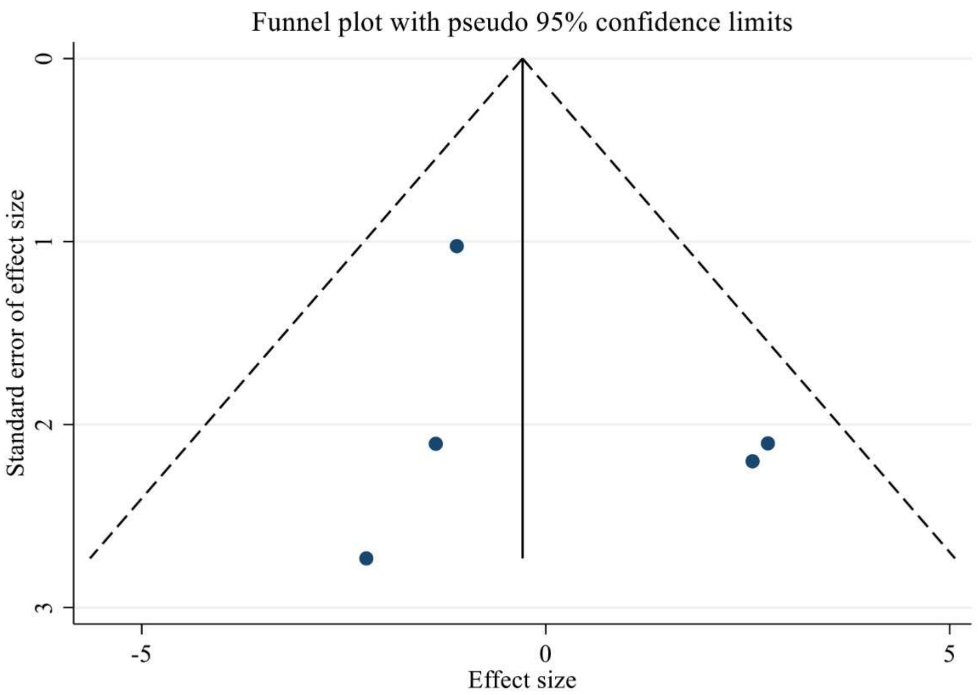
Funnel plot with pseudo 95% confidence limits
Standard error of effect size
0
1
2
3
-5
0
5
Effect size

2. w

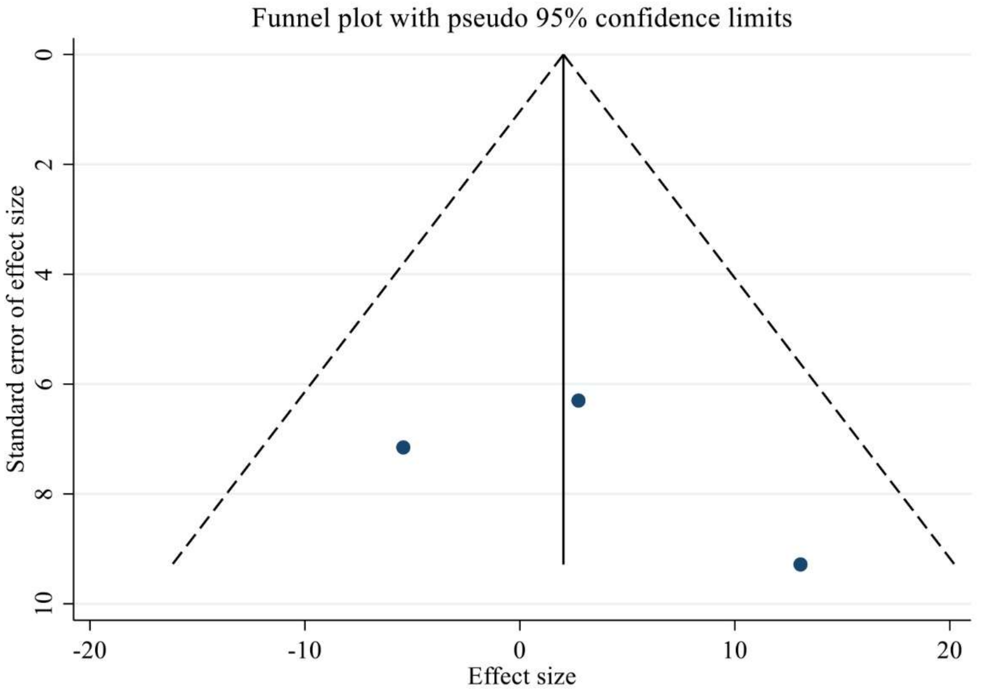

a: body weight (BW); b: body mass index (BMI); c: waist circumference (WC); d: fasting plasma glucose (FPG); e: oral glucose tolerance test (OGTT); f: hemoglobin A1c (HbA1c); g: fasting insulin; h: homeostatic model assessment of insulin resistance (HOMA-IR); i: triglyceride (TG); j: total cholesterol (TC); k: low-density lipoprotein cholesterol (LDL-C); l: high-density lipoprotein cholesterol (HDL-C); m: systolic blood pressure (SBP); n: diastolic blood pressure (DBP); o: heart rate (HR); p: C-reactive protein (CRP); q: interlukin-6 (IL-6); r: tumor necrosis α (TNF-α); s: adiponectin; t: leptin; u: alanine aminotransferase (ALT); v: aspartate aminotransferase (AST); w: gamma-glutamyl transferase (GGT)

Supplementary Figure S3. Dose-response relations between ginseng dosage (mg/day) and mean difference in each outcomes.

3. a

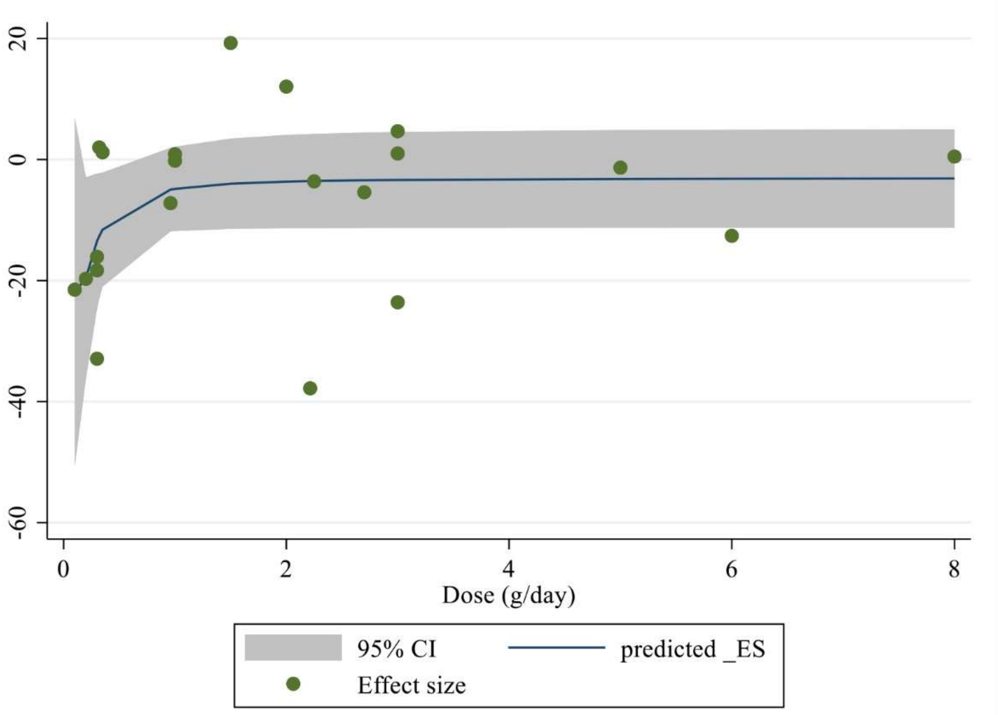

3. b

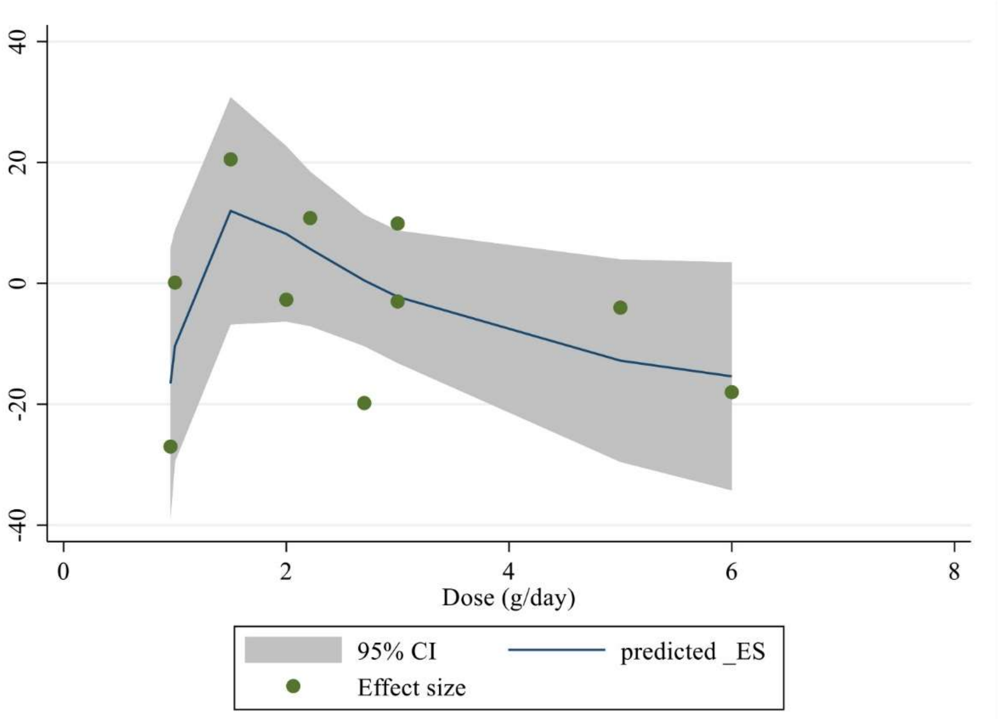

3. c

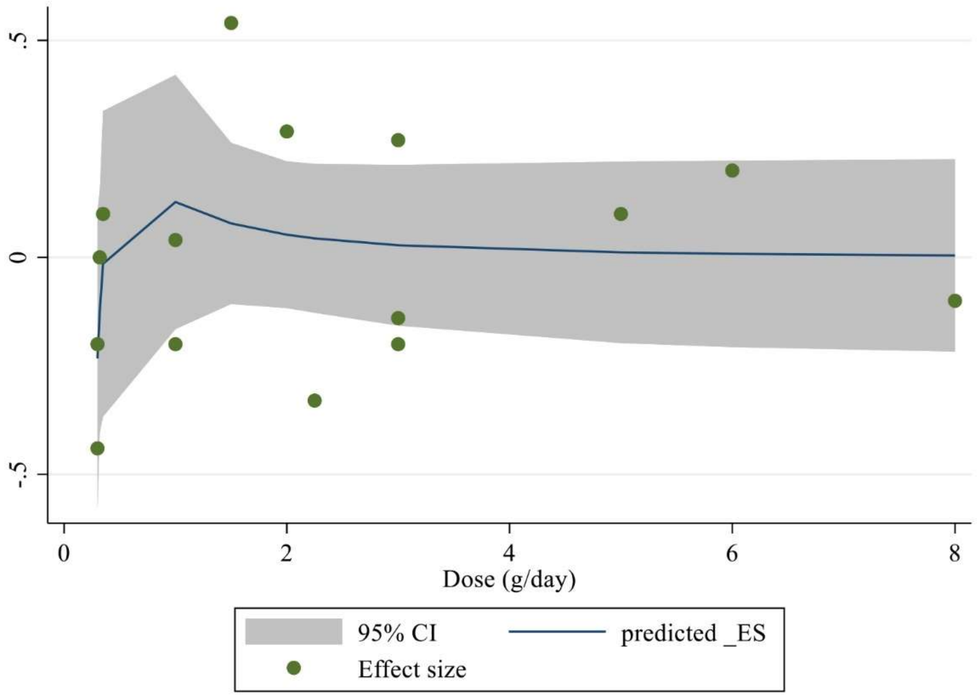

.5
0
-.5
0
2
4
6
8
Dose (g/day)
95% CI
predicted _ES
Effect size

3. d

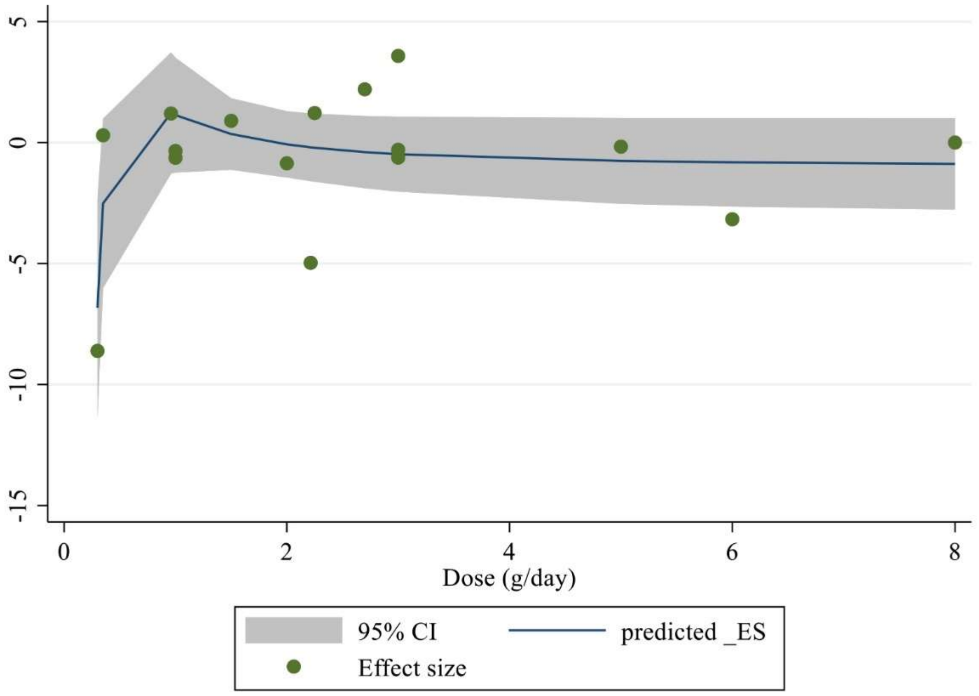

5
0
-5
-10
-15
0
2
4
6
8
Dose (g/day)
95% CI
predicted _ES
Effect size

3. e

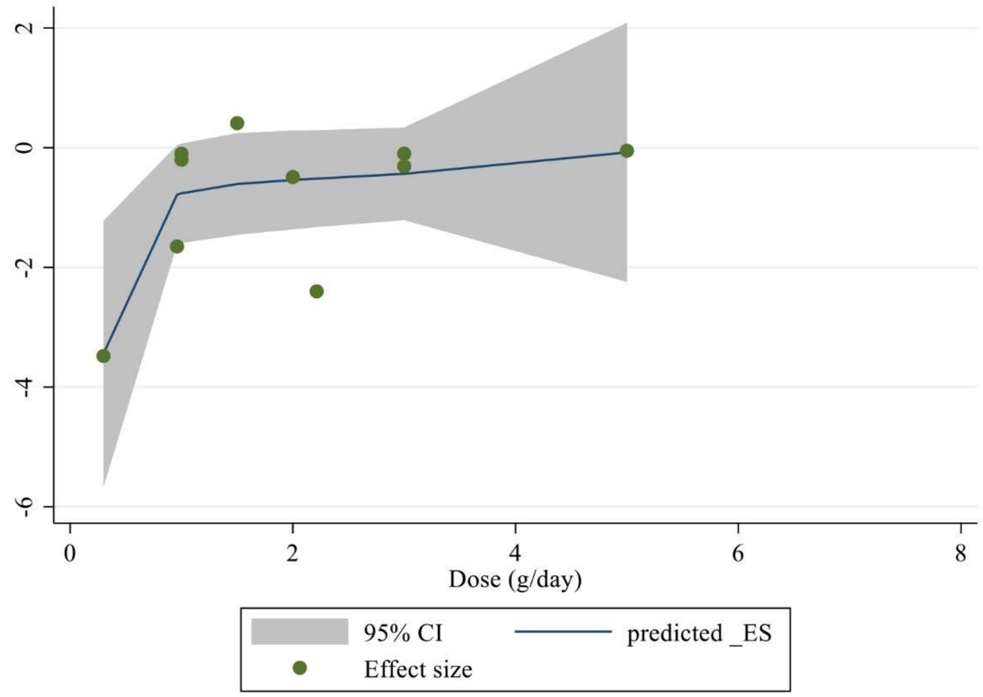

2
0
-2
-4
-6
0
2
4
6
8
Dose (g/day)
95% CI
predicted _ES
Effect size

3. f

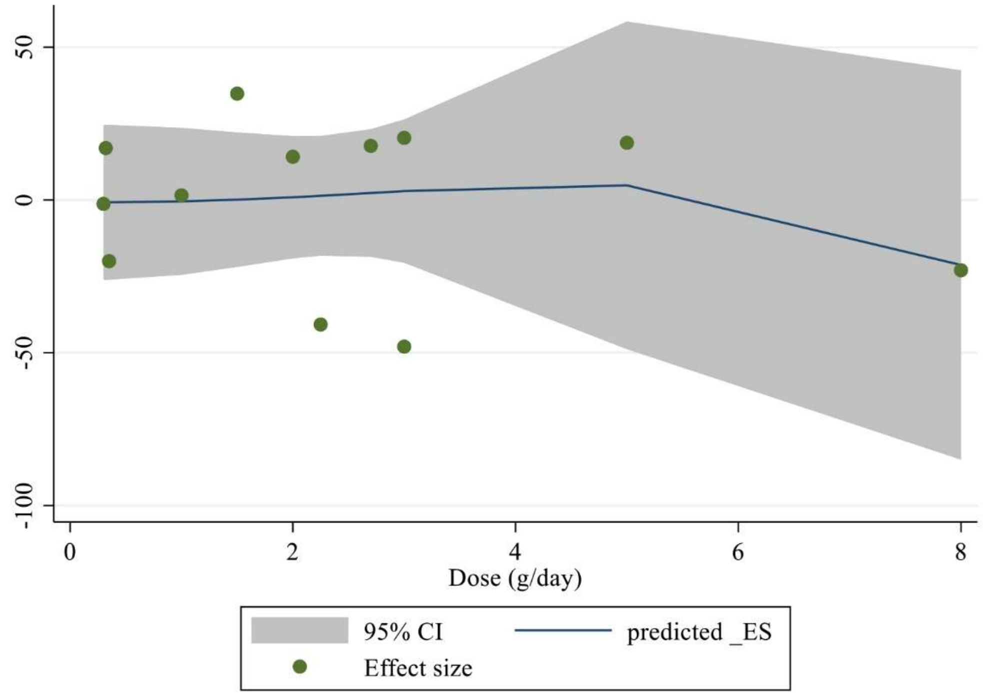

50
0
-50
-100
0
2
4
6
8
Dose (g/day)
95% CI
predicted _ES
Effect size

3. g

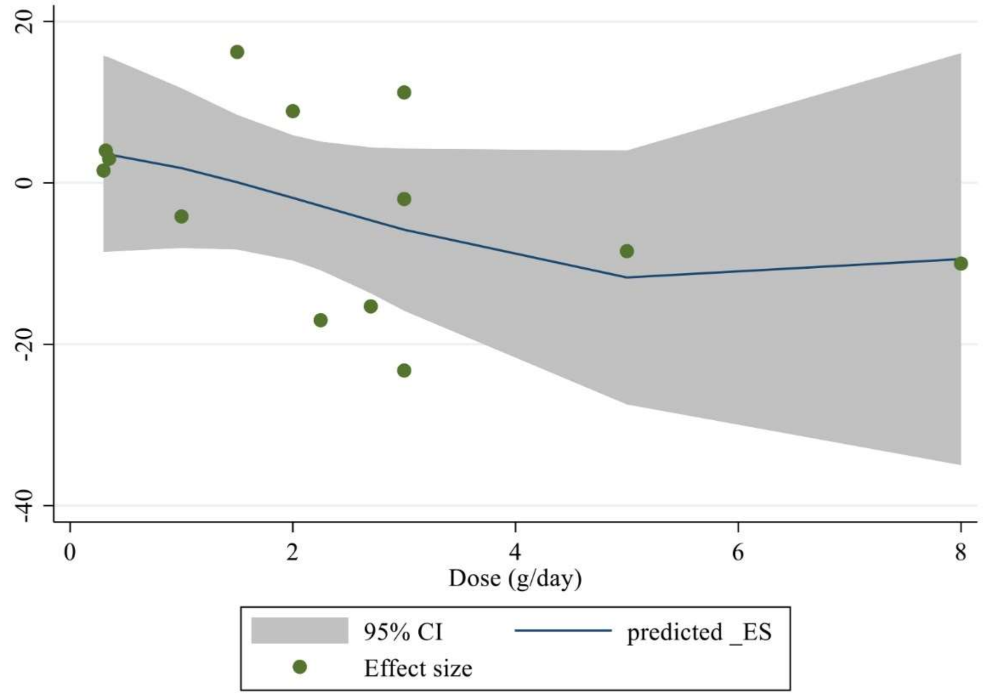

20
0
-20
-40
0
2
4
6
8
Dose (g/day)
95% CI
predicted _ES
Effect size

3. h

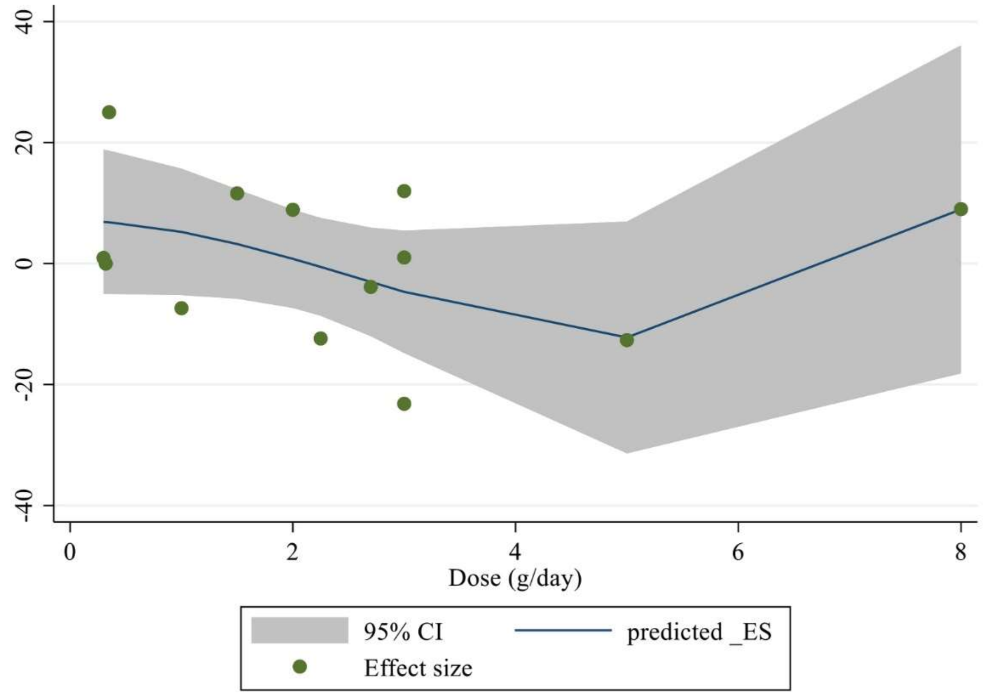

40
20
0
-20
-40
0
2
4
6
8
Dose (g/day)
95% CI
predicted _ES
Effect size

3. i

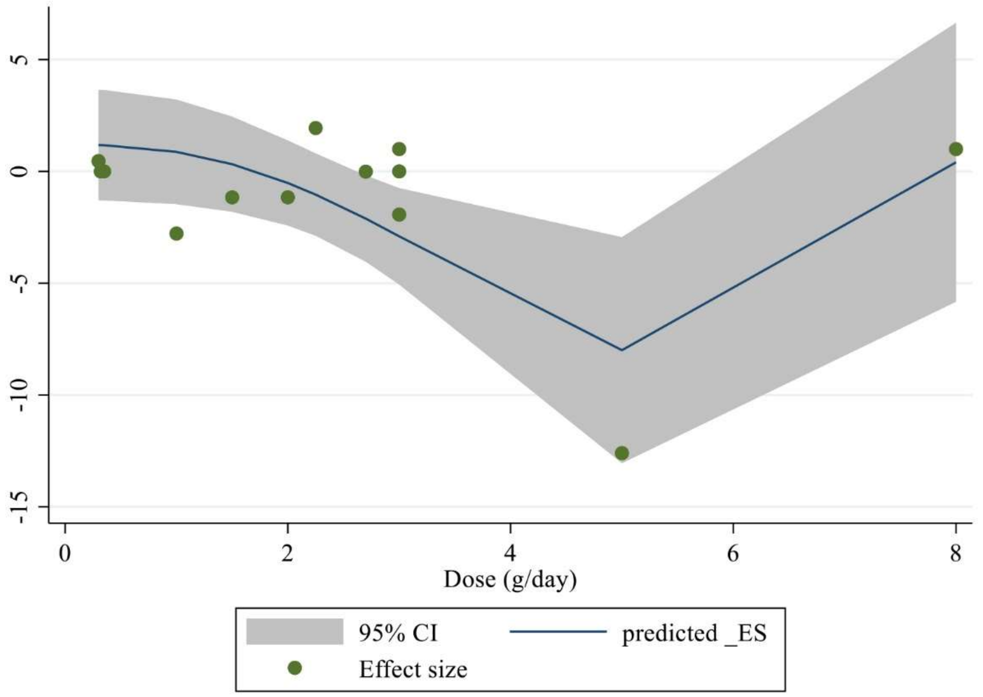
5
0
-5
-10
-15
0
2
4
6
8
Dose (g/day)
95% CI
predicted _ES
Effect size

4. j

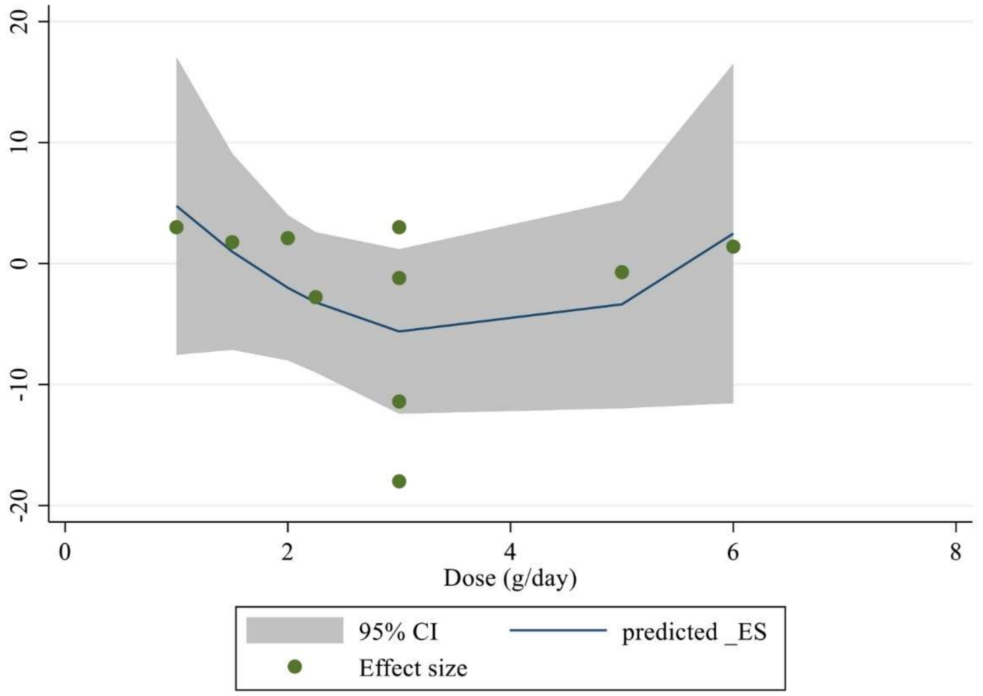
20
10
0
-10
-20
0
2
4
6
8
Dose (g/day)
95% CI
predicted _ES
Effect size

3. k

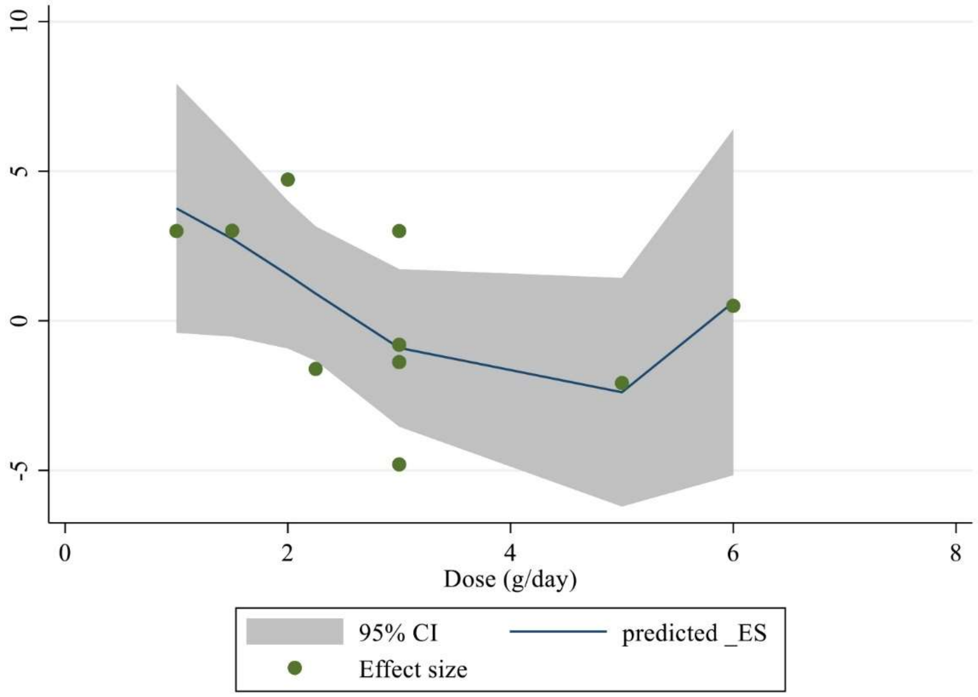

a: fasting plasma glucose (FPG); b: oral glucose tolerance test (OGTT); c: hemoglobin A1c (HbA1c); d: fasting insulin; e: homeostatic model assessment of insulin resistance (HOMA-IR); f: triglyceride (TG); g: total cholesterol (TC); h: low-density lipoprotein cholesterol (LDL-C); i: high-density lipoprotein cholesterol (HDL-C); j: systolic blood pressure (SBP); k: diastolic blood pressure (DBP)

Supplementary Figure S4. Dose-response relations between duration of ginseng (week) and mean difference in each outcomes.

4. a

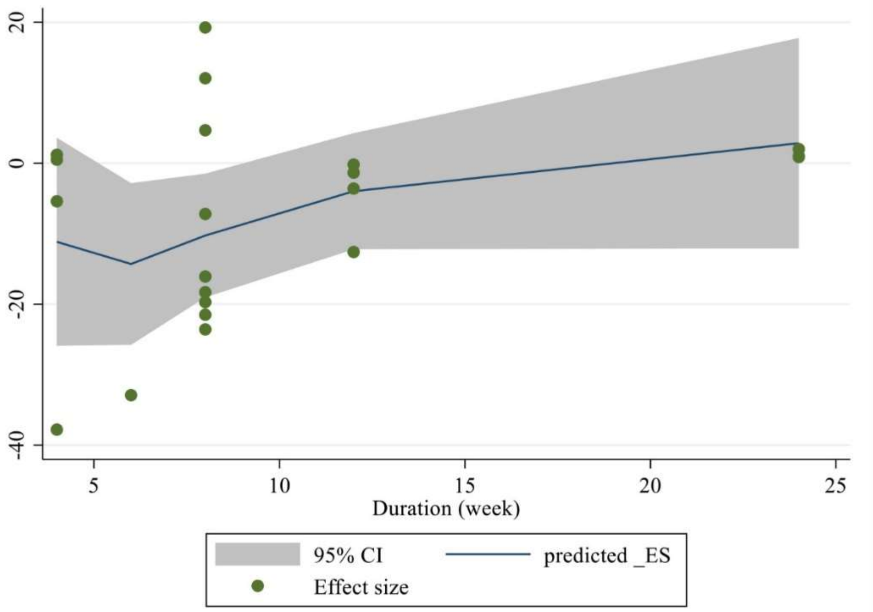

4. b

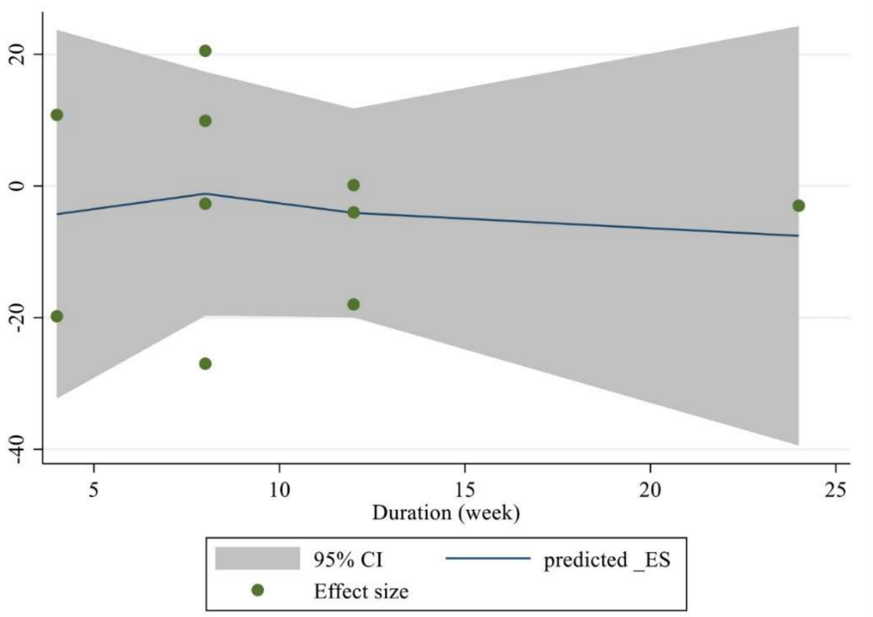

4. c

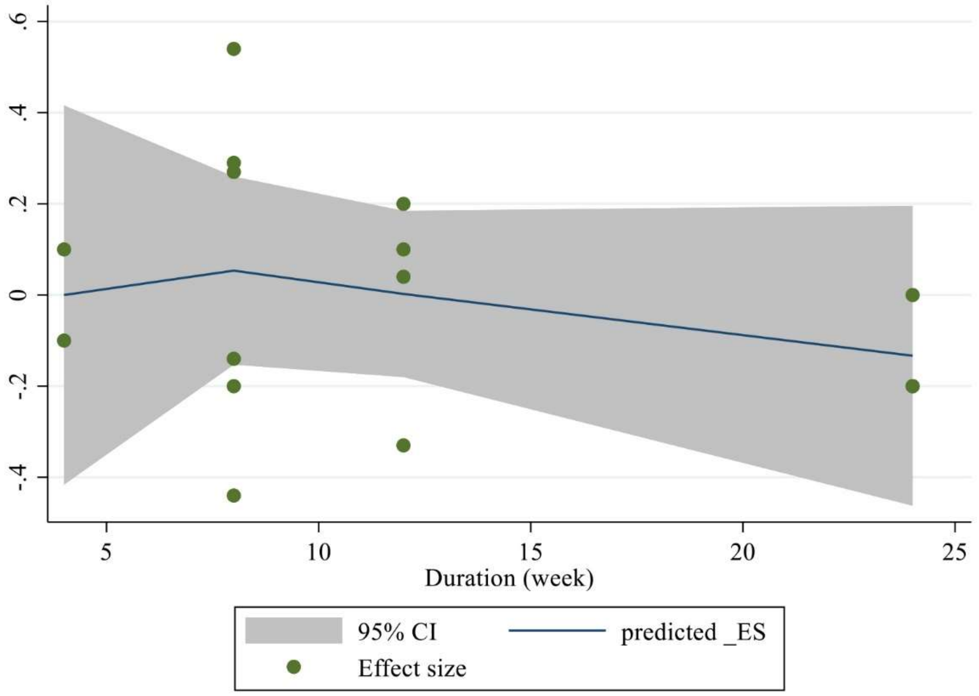

.6
.4
.2
0
-.2
-.4
5
10
15
20
25
Duration (week)
95% CI
predicted _ES
Effect size

4. d

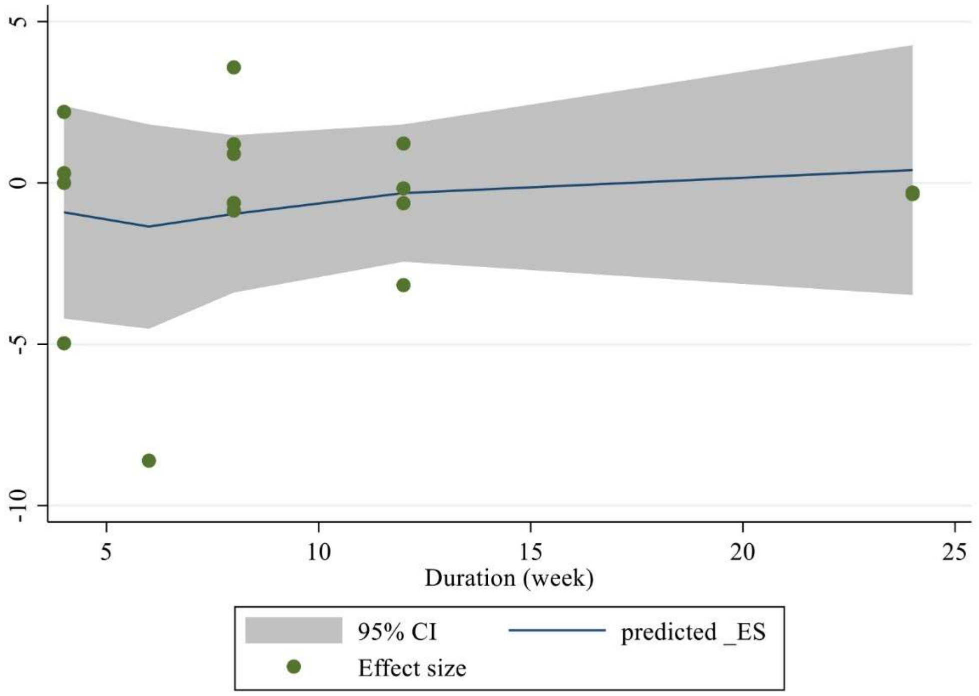

5
0
-5
-10
5
10
15
20
25
Duration (week)
95% CI
predicted _ES
Effect size

4. e

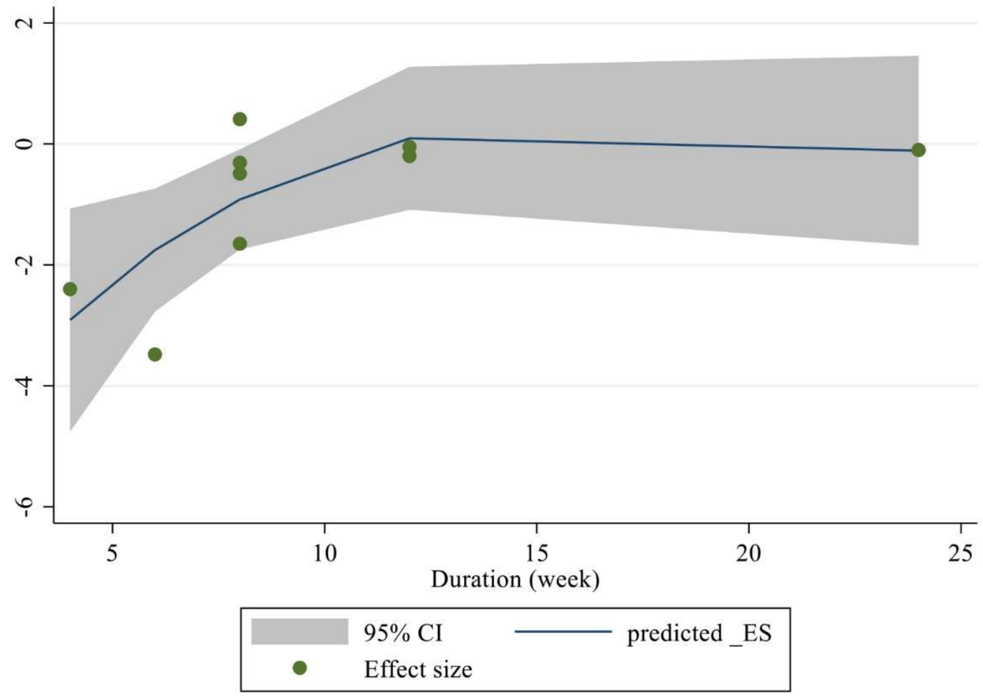

2
0
-2
-4
-6
5
10
15
20
25
Duration (week)
95% CI
predicted _ES
Effect size

4. f

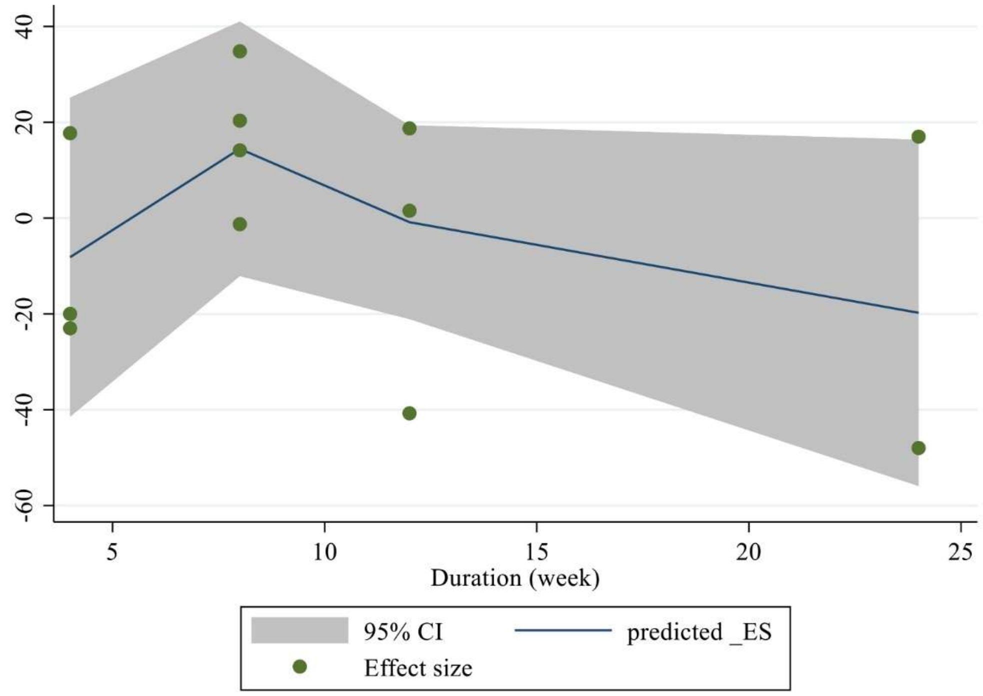

40
20
0
-20
-40
-60
5
10
15
20
25
Duration (week)
95% CI
predicted _ES
Effect size

4. g

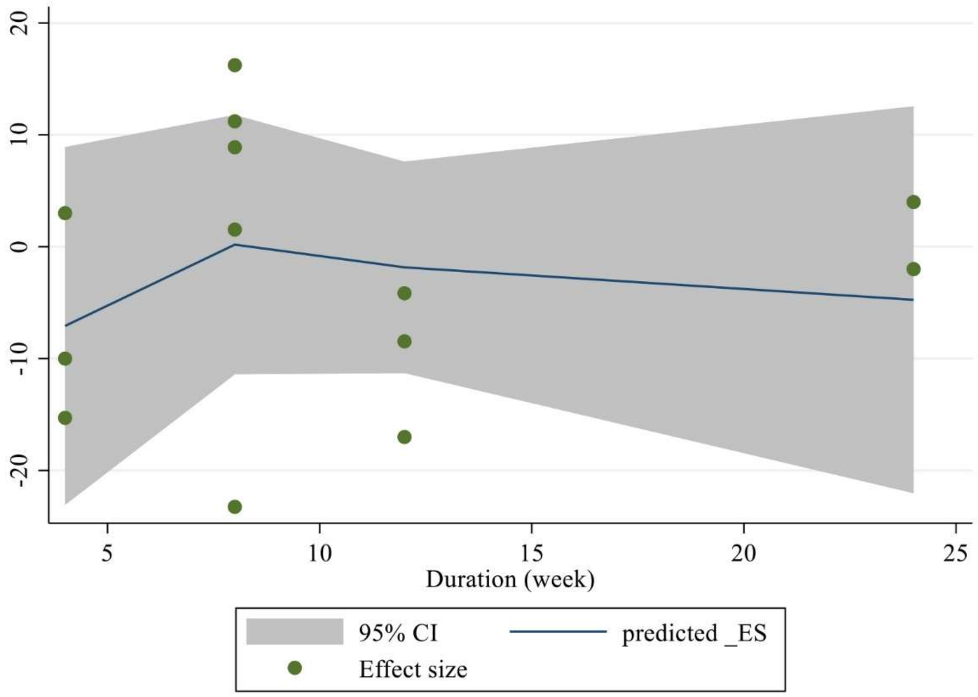
20
10
0
-10
-20
5
10
15
20
25
Duration (week)
95% CI
predicted _ES
Effect size

4. h

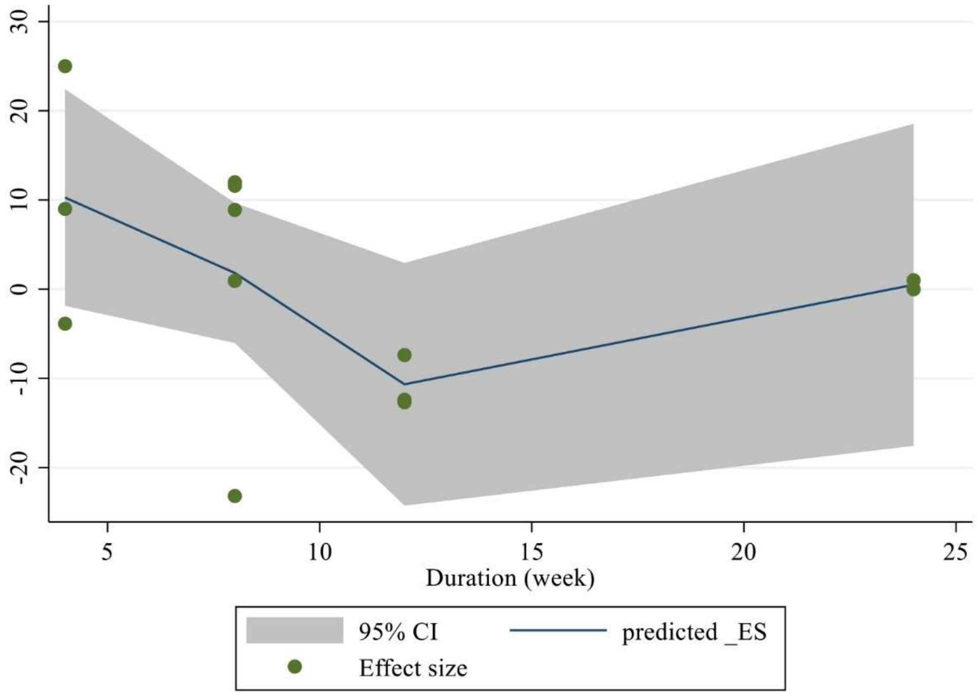
30
20
10
0
-10
-20
5
10
15
20
25
Duration (week)
95% CI
predicted _ES
Effect size

4. i

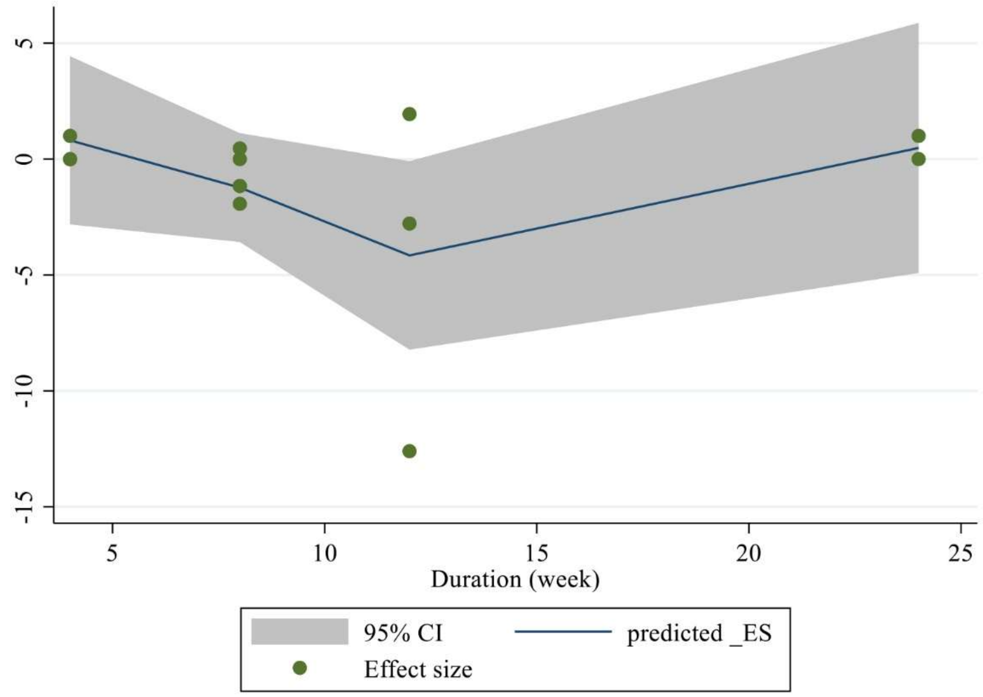
5
0
-5
-10
-15
5
10
15
20
25
Duration (week)
95% CI
predicted _ES
Effect size

4. j

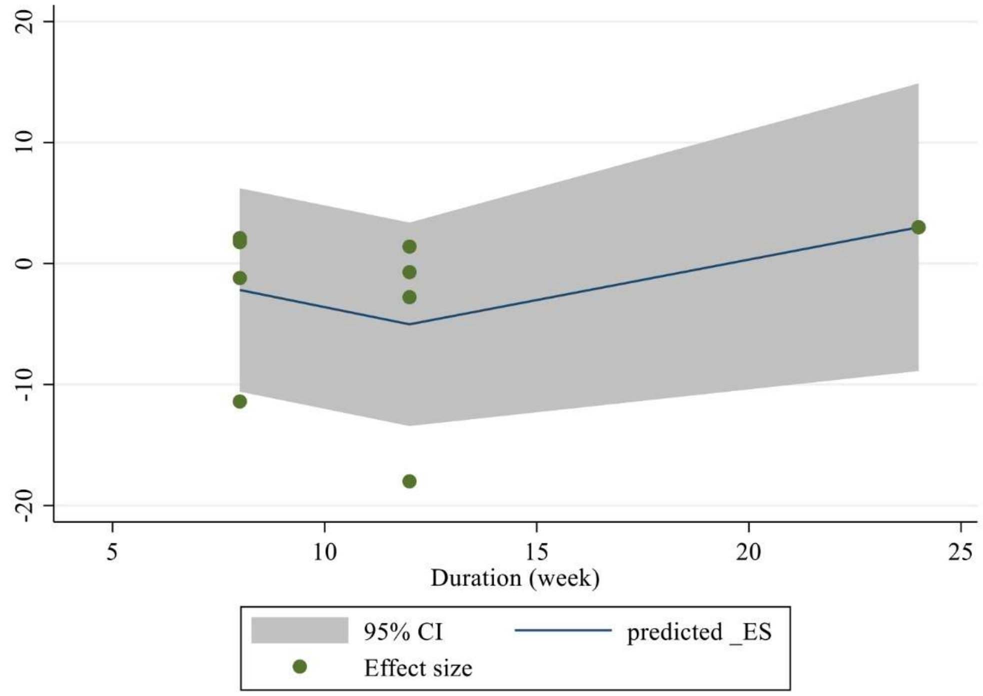
20
10
0
-10
-20
5
10
15
20
25
Duration (week)
95% CI
predicted _ES
Effect size

4. k

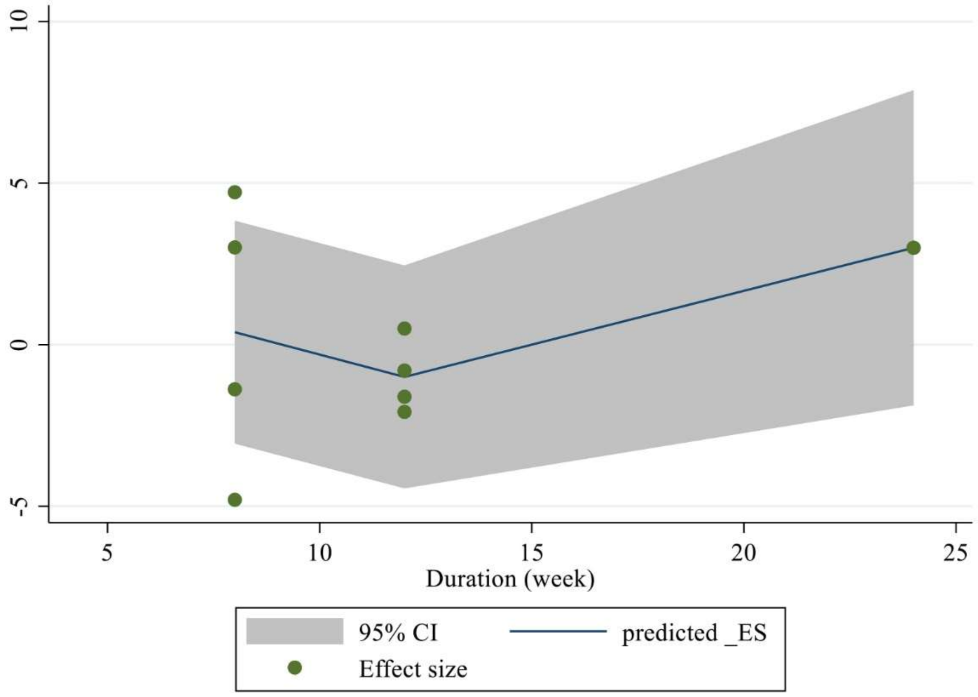

a: fasting plasma glucose (FPG); b: oral glucose tolerance test (OGTT); c: hemoglobin A1c (HbA1c); d: fasting insulin; e: homeostatic model assessment of insulin resistance (HOMA-IR); f: triglyceride (TG); g: total cholesterol (TC); h: low-density lipoprotein cholesterol (LDL-C); i: high-density lipoprotein cholesterol (HDL-C); j: systolic blood pressure (SBP); k: diastolic blood pressure (DBP)

Supplementary Figure S5. Random-effects meta-regression plots of the association between dose of ginseng (mg/day) and weighted mean difference of each outcome.

5. a

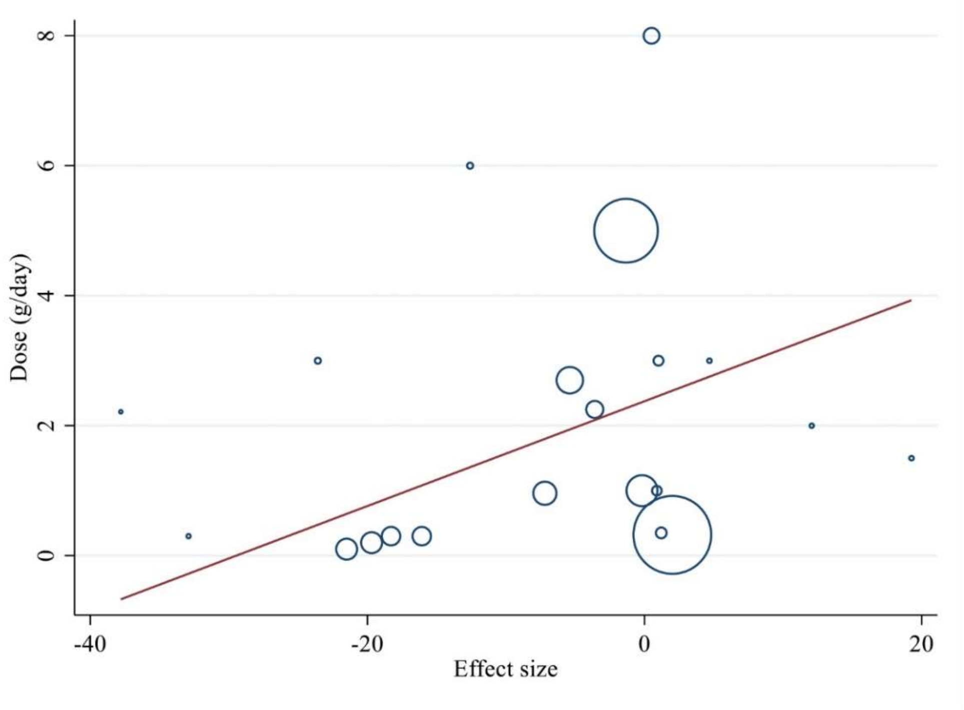

5. b

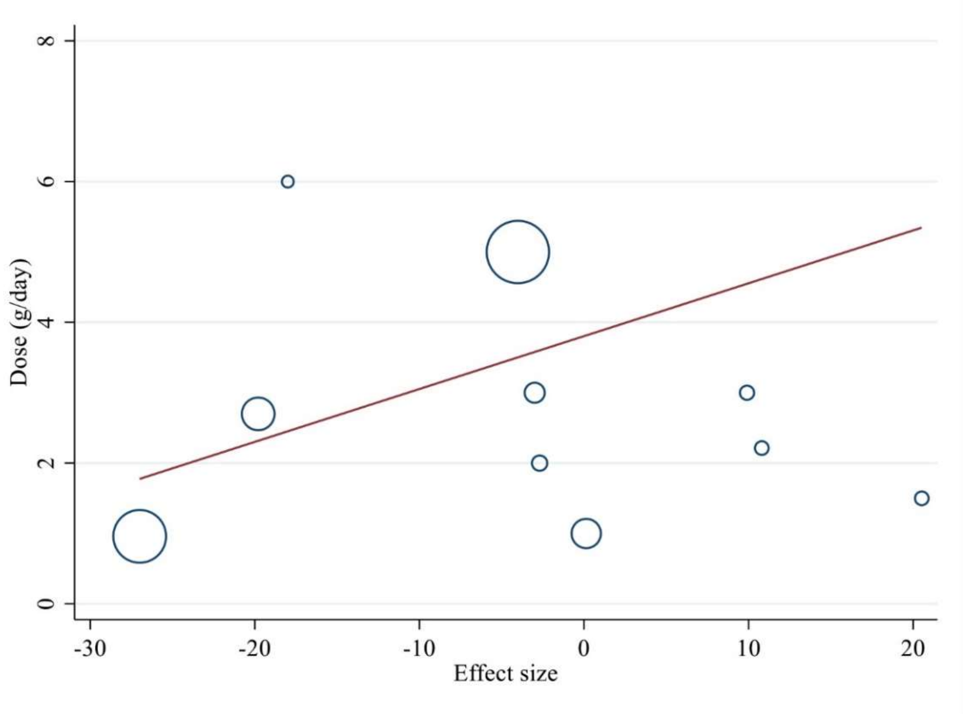

5. c

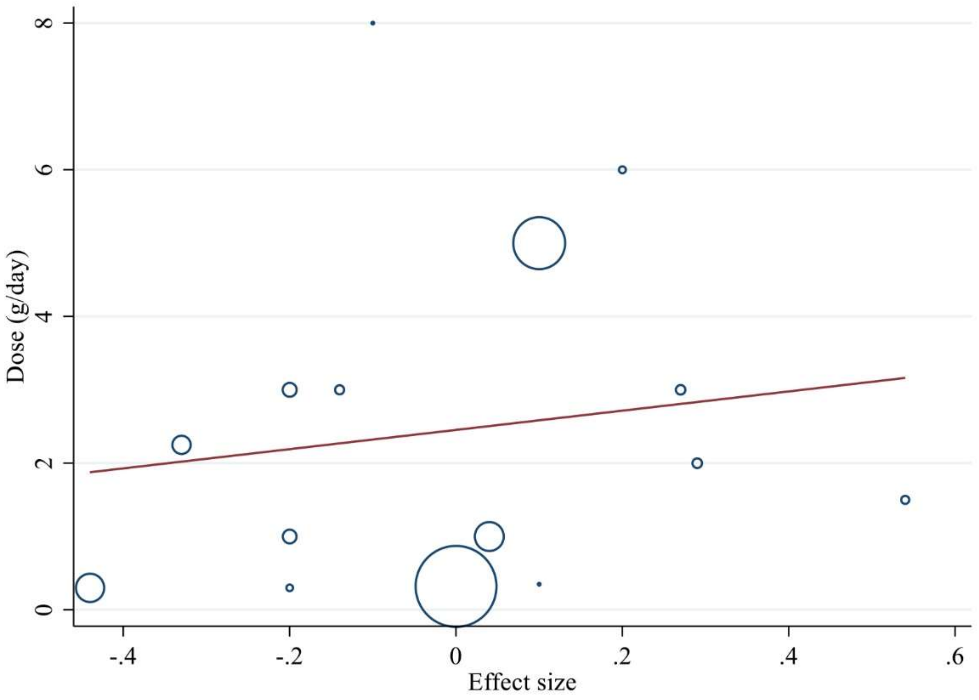
Dose (g/day)
0
2
4
6
8
-.4
-.2
0
.2
.4
.6
Effect size

5. d

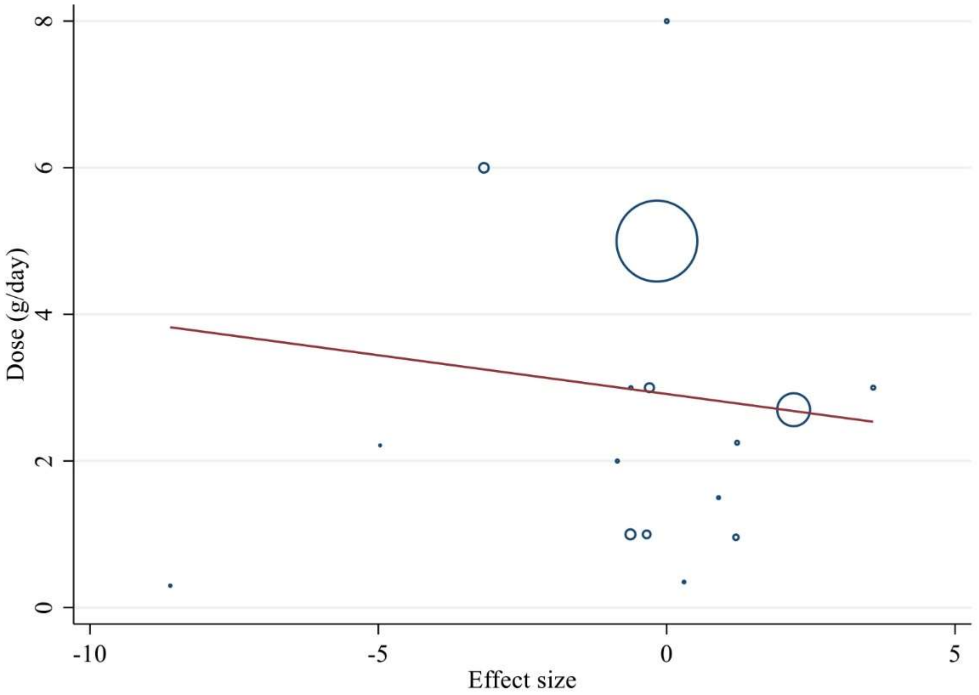
Dose (g/day)
0
2
4
6
8
-10
-5
0
5
Effect size

5. e

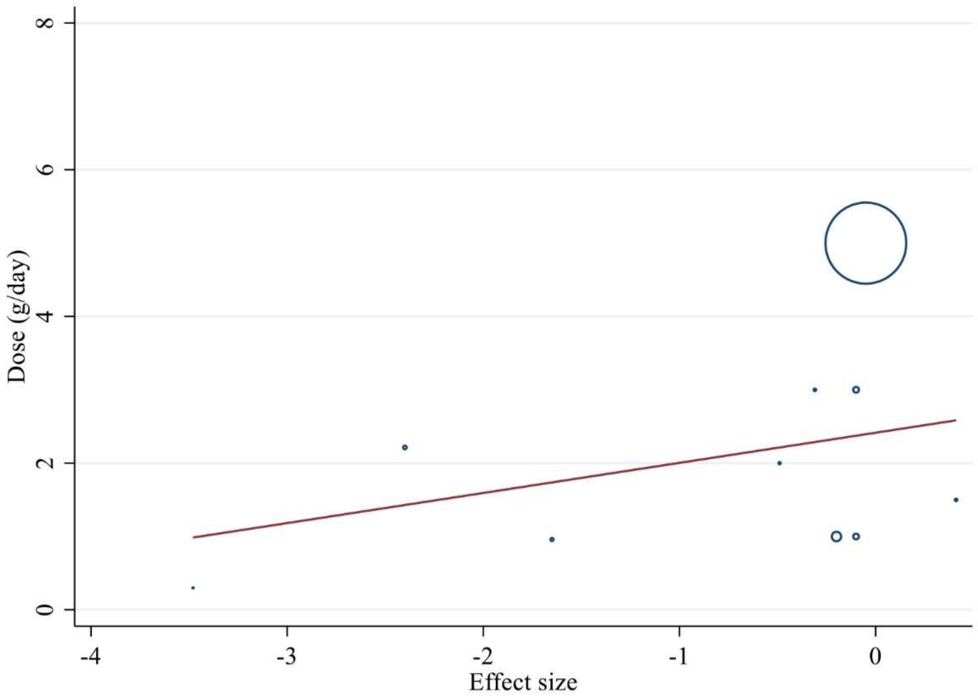

Dose (g/day)
0
2
4
6
8
-4
-3
-2
-1
0
Effect size

5. f

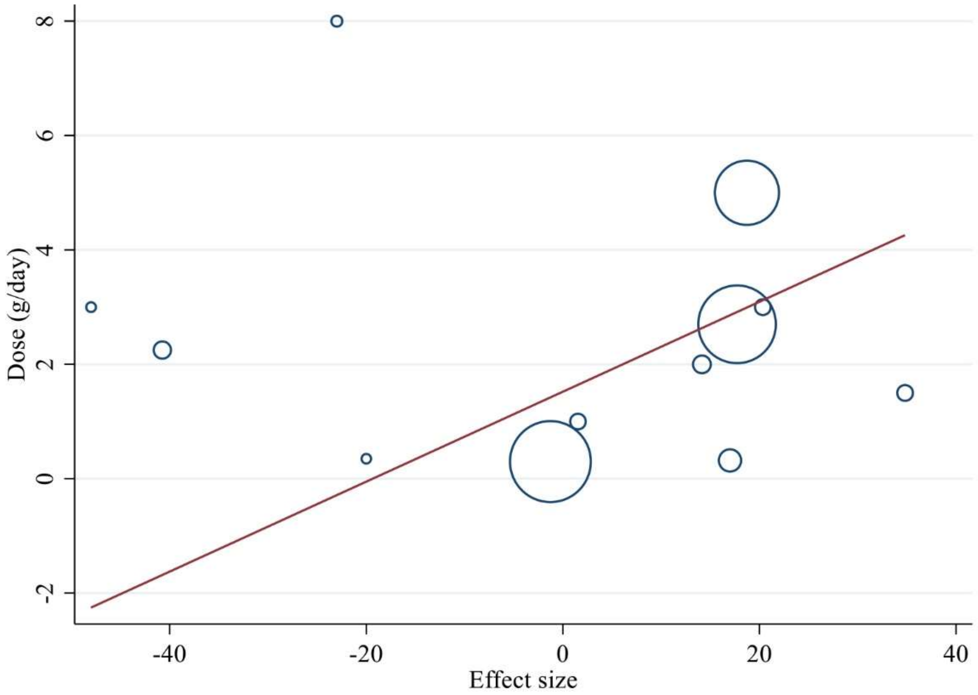

Dose (g/day)
-2
0
2
4
6
8
-40
-20
0
20
40
Effect size

5. g

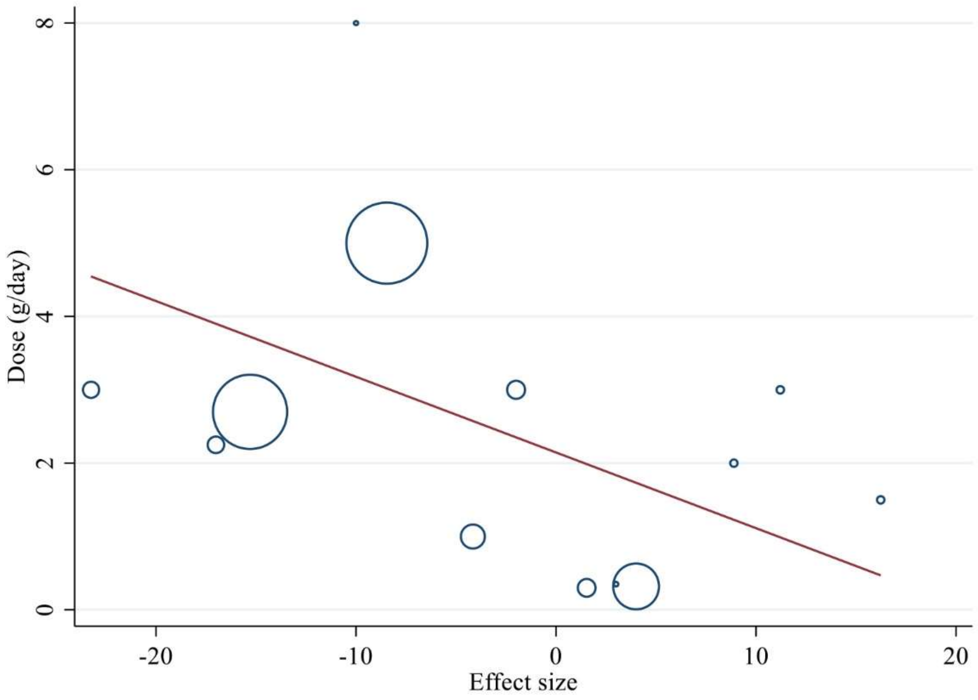

Dose (g/day)
8
6
4
2
0
-20
-10
0
10
20
Effect size

5. h

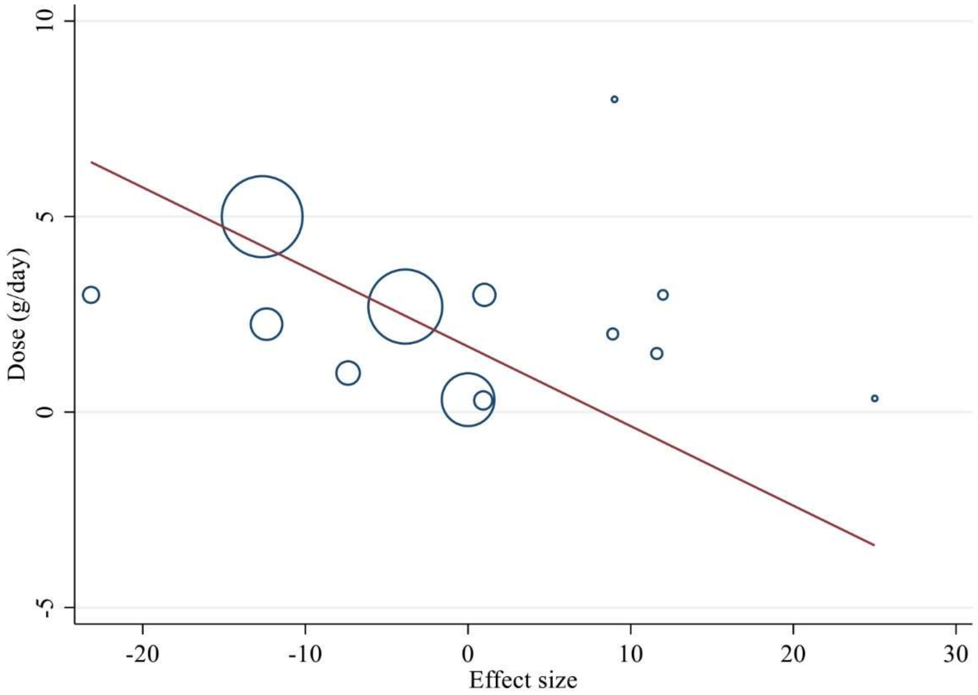

Dose (g/day)
10
5
0
-5
-20
-10
0
10
20
30
Effect size

5. i

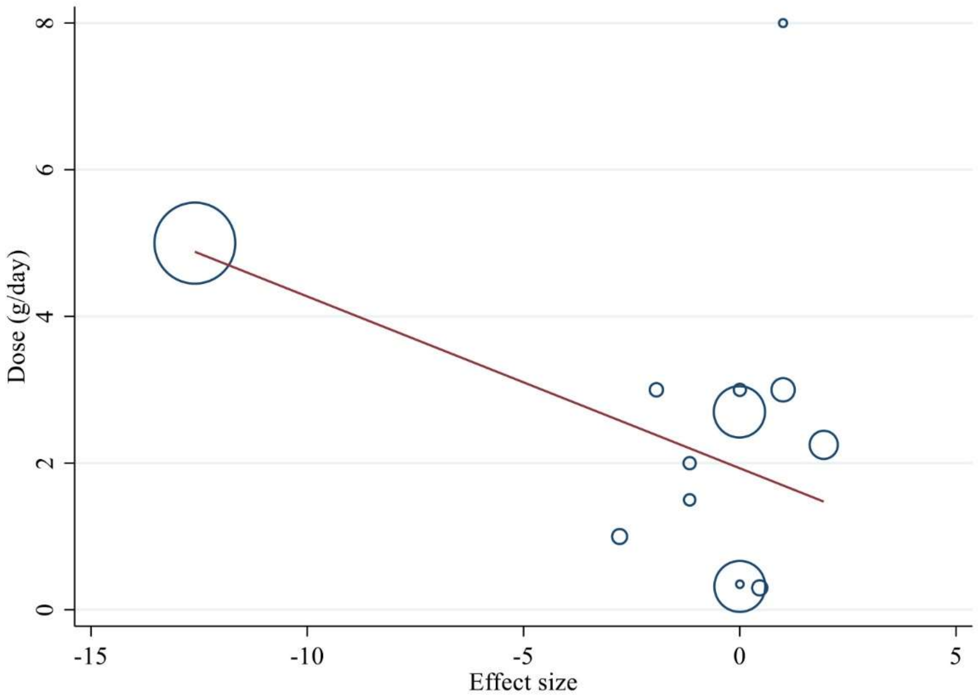

8
6
4
2
0
Dose (g/day)
-15
-10
-5
0
5
Effect size

5. j

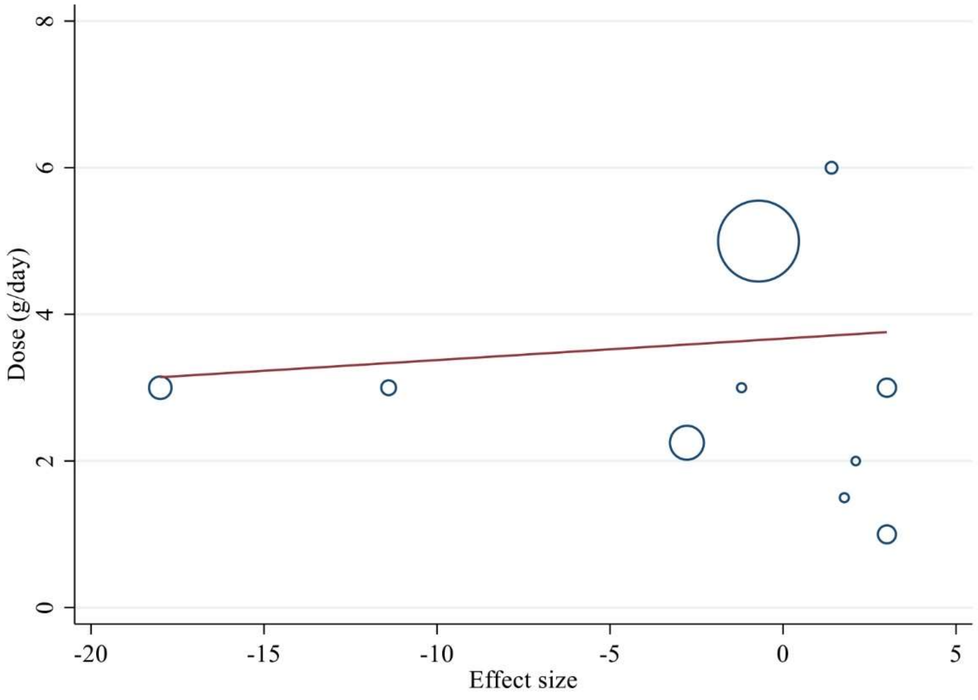

8
6
4
2
0
Dose (g/day)
-20
-15
-10
-5
0
5
Effect size

5. k

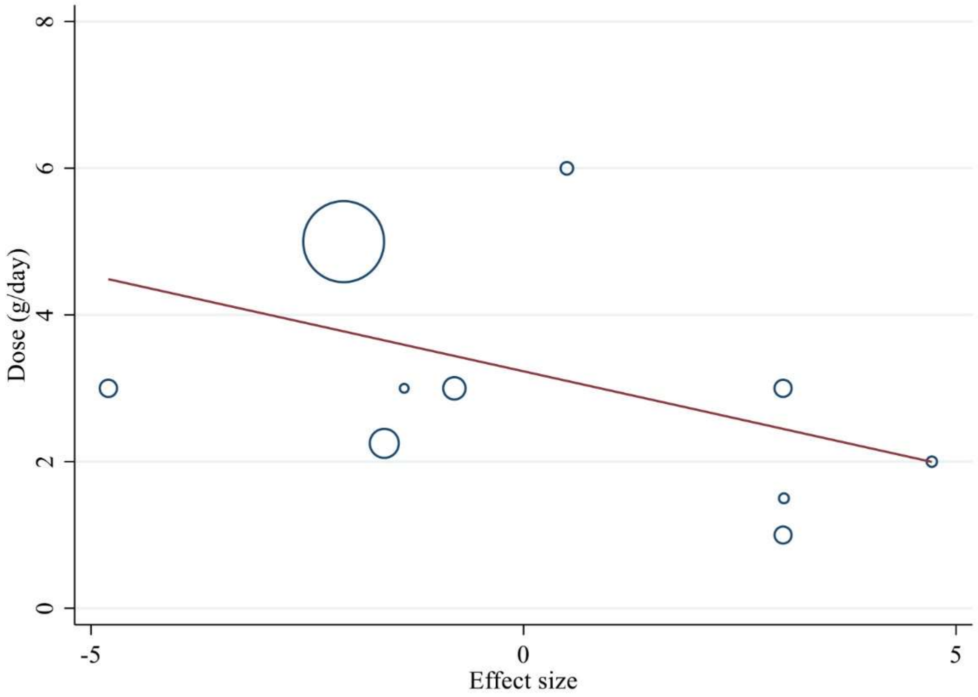

a: fasting plasma glucose (FPG); b: oral glucose tolerance test (OGTT); c: hemoglobin A1c (HbA1c); d: fasting insulin; e: homeostatic model assessment of insulin resistance (HOMA-IR); f: triglyceride (TG); g: total cholesterol (TC); h: low-density lipoprotein cholesterol (LDL-C); i: high-density lipoprotein cholesterol (HDL-C); j: systolic blood pressure (SBP); k: diastolic blood pressure (DBP)

Supplementary Figure S6. Random-effects meta-regression plots of the association between duration of ginseng (week) and weighted mean difference of each outcome.

6. a

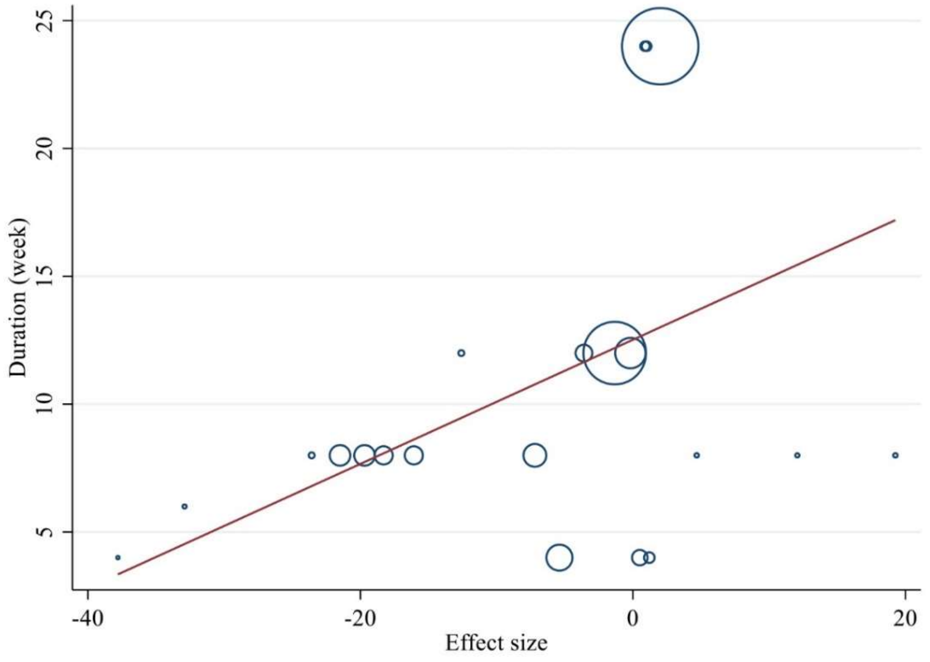

6. b

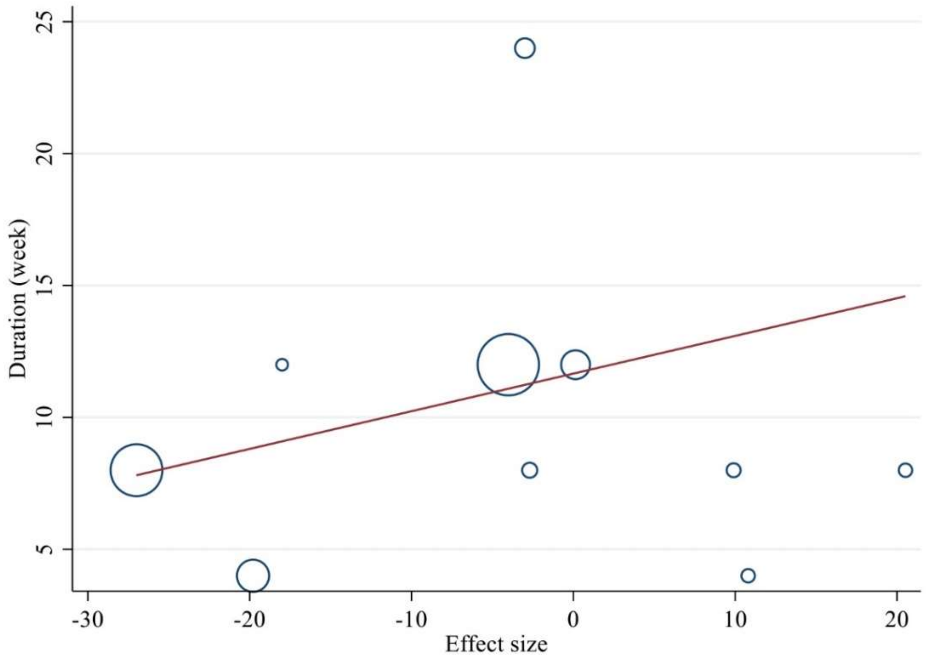

6. c

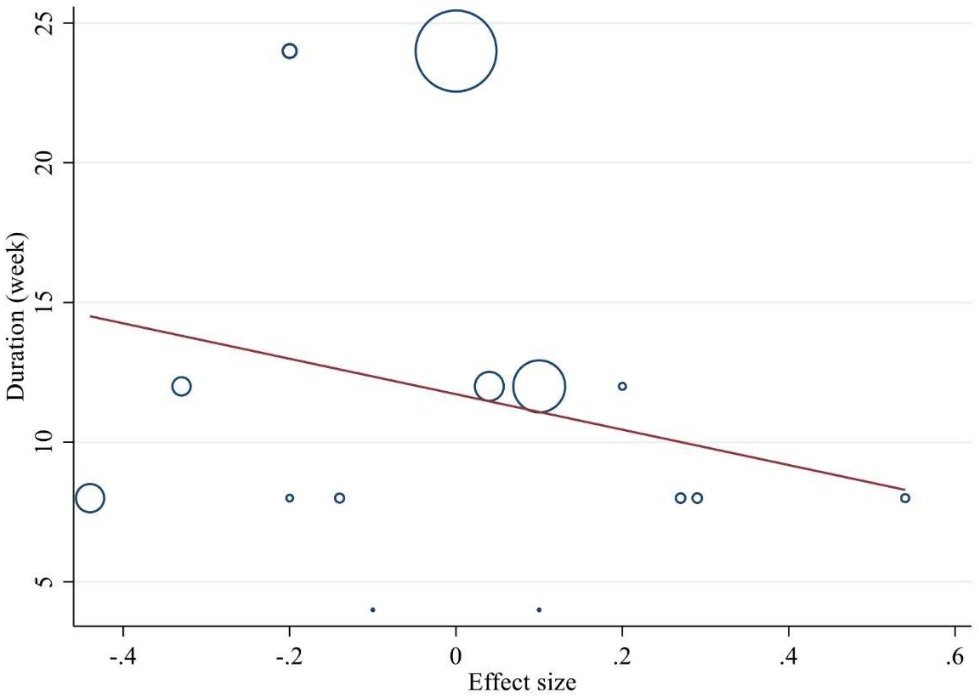

25
20
15
10
5
Duration (week)
-.4
-.2
0
.2
.4
.6
Effect size

6. d

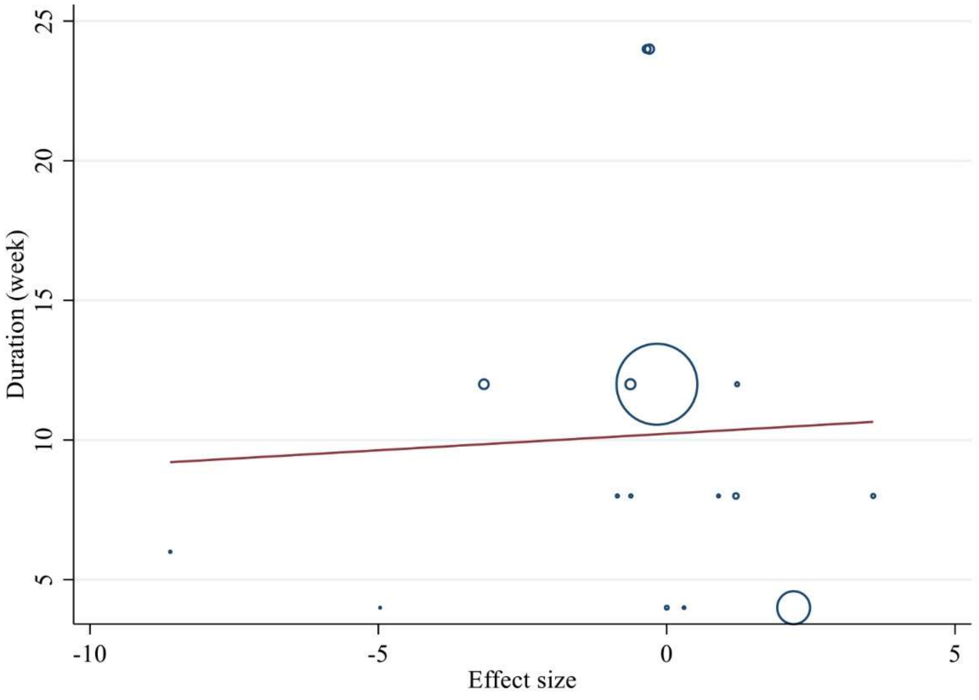

25
20
15
10
5
Duration (week)
-10
-5
0
5
Effect size

6. e

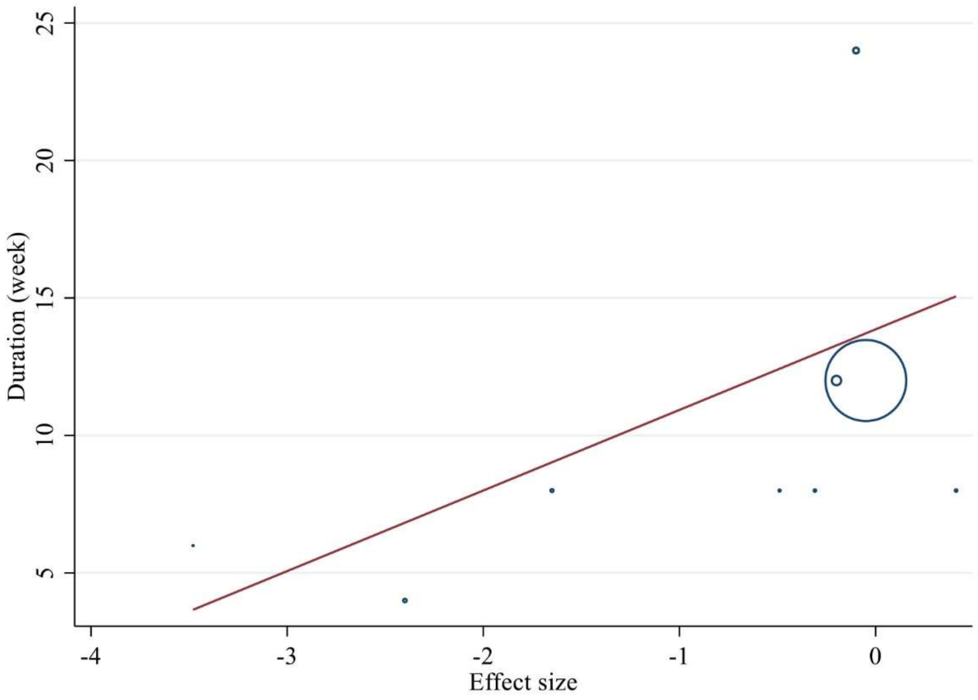

Duration (week)
25
20
15
10
5
-4
-3
-2
-1
0
Effect size

6. f

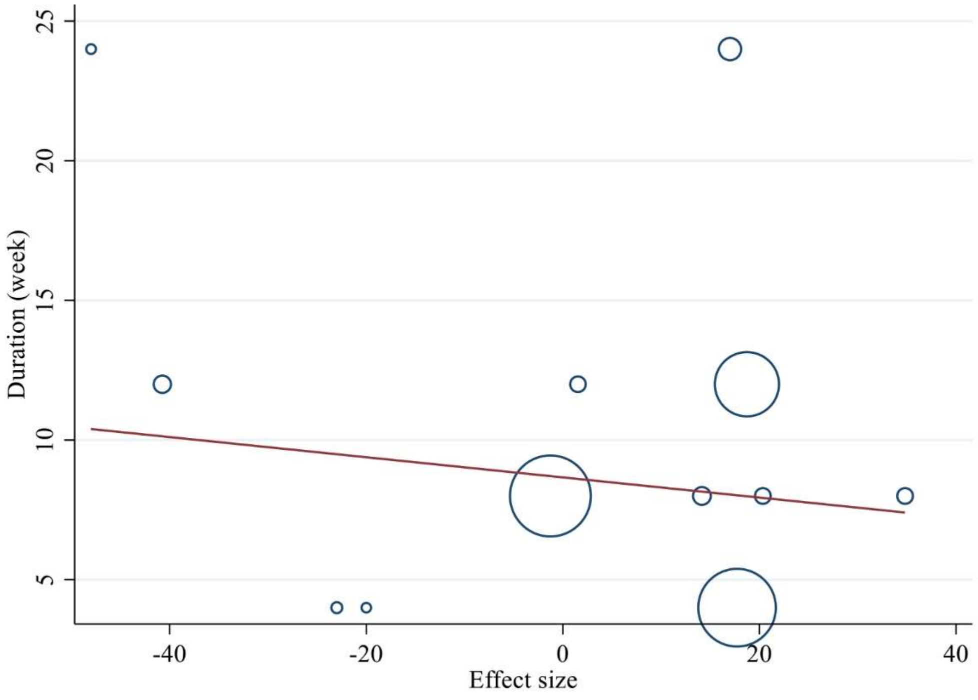

Duration (week)
25
20
15
10
5
-40
-20
0
20
40
Effect size

6. g

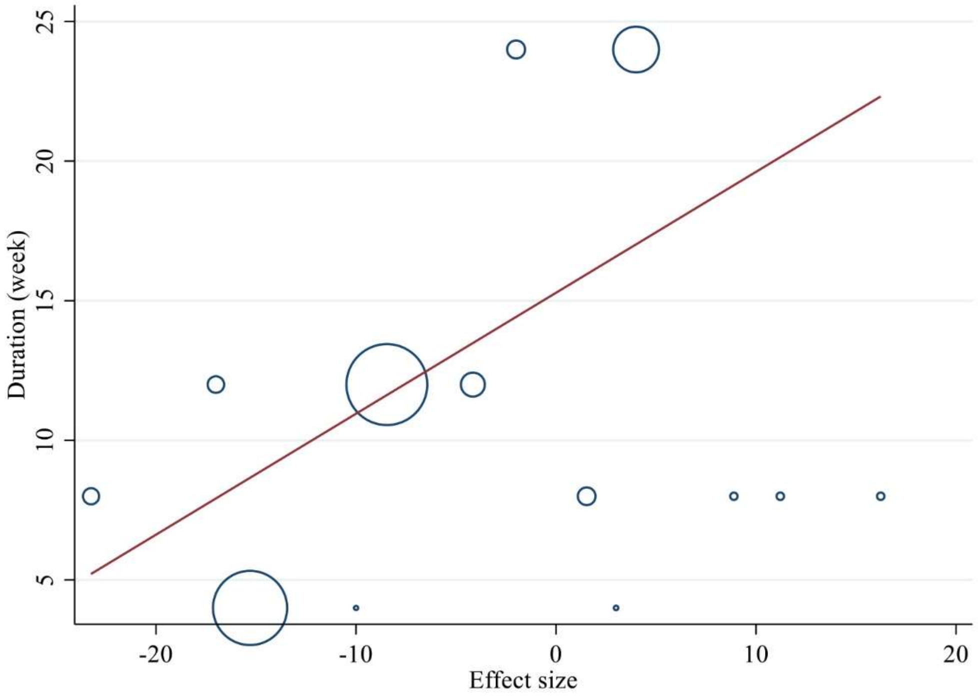

25
20
15
10
5
Duration (week)
-20
-10
0
10
20
Effect size

6. h

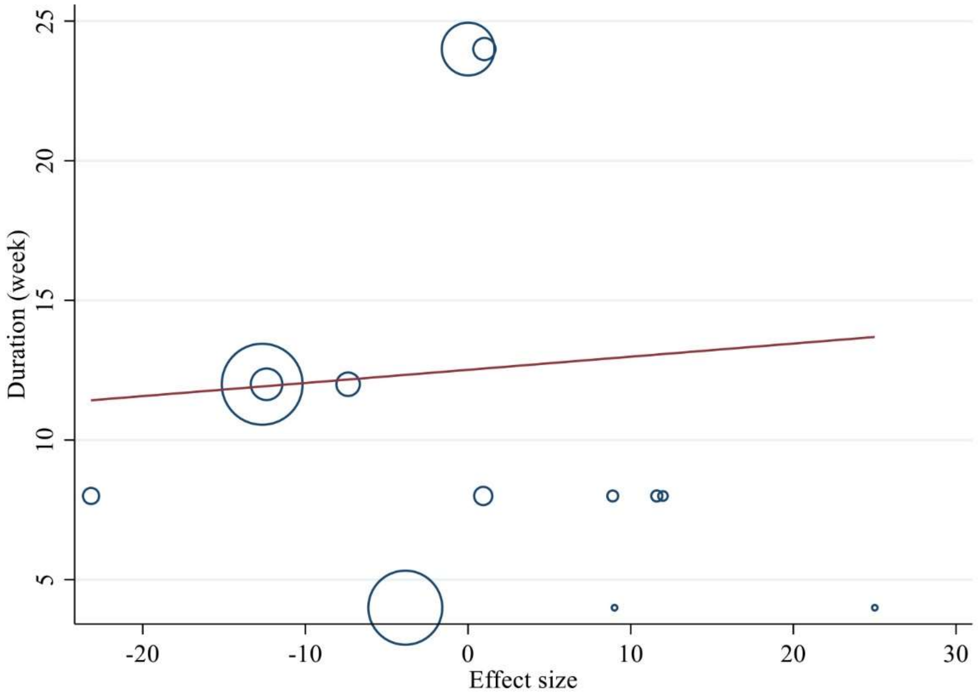

25
20
15
10
5
Duration (week)
-20
-10
0
10
20
30
Effect size

6. i

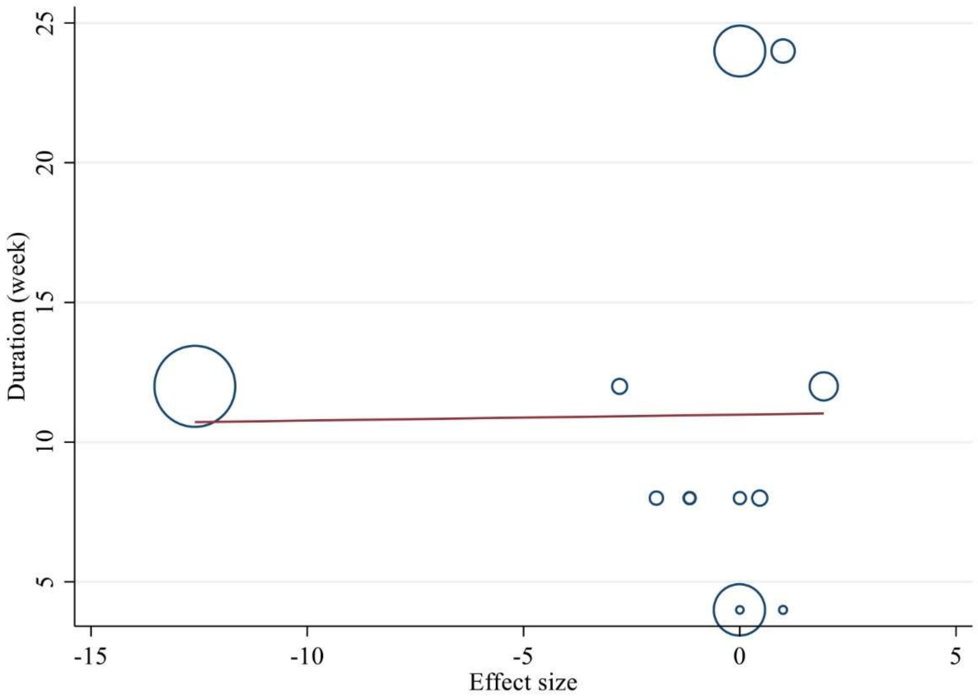

25
20
15
10
5
Duration (week)
-15
-10
-5
0
5
Effect size

6. j

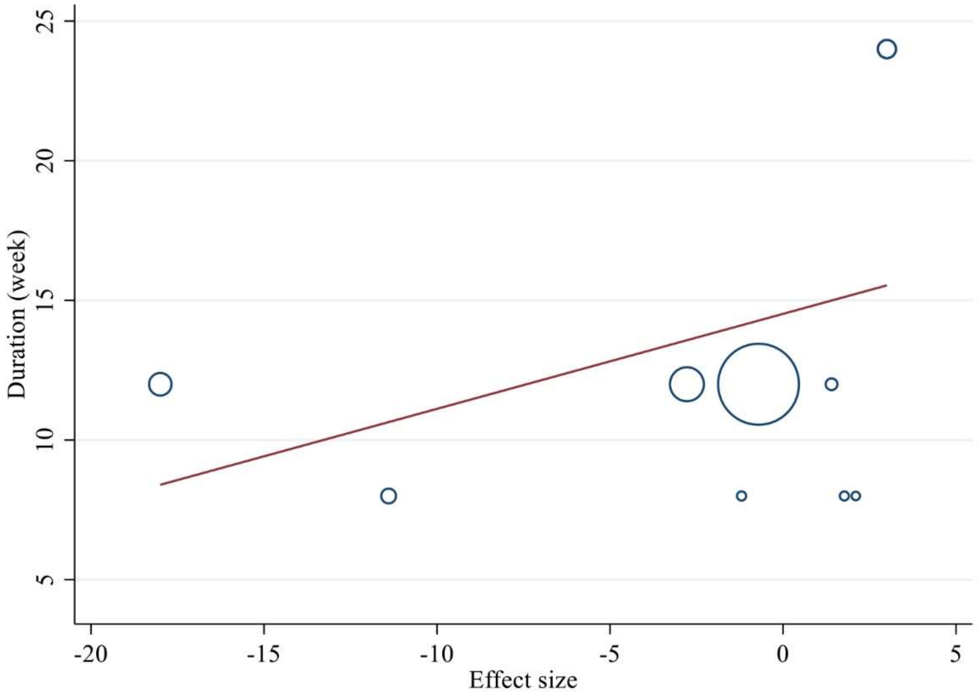

25
20
15
10
5
Duration (week)
-20
-15
-10
-5
0
5
Effect size

6. k

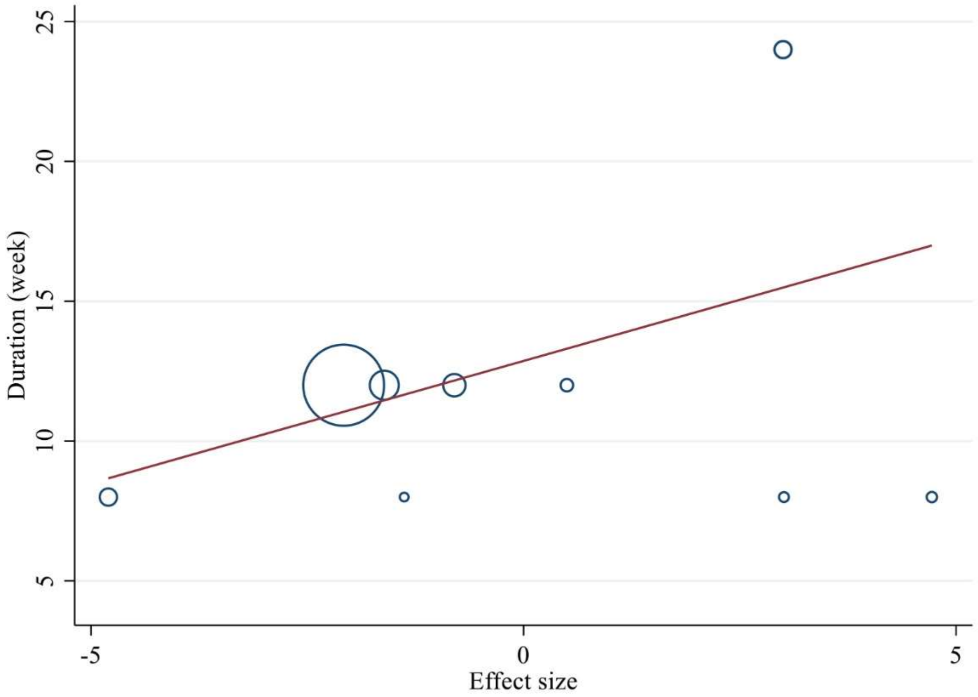

a: fasting plasma glucose (FPG); b: oral glucose tolerance test (OGTT); c: hemoglobin A1c (HbA1c); d: fasting insulin; e: homeostatic model assessment of insulin resistance (HOMA-IR); f: triglyceride (TG); g: total cholesterol (TC); h: low-density lipoprotein cholesterol (LDL-C); i: high-density lipoprotein cholesterol (HDL-C); j: systolic blood pressure (SBP); k: diastolic blood pressure (DBP)

Supplementary Table S1. Search terms used across the various databases.

PubMed	((Ginseng[Title/Abstract] OR ginsenoside[Title/Abstract]) AND ("Type 2 diabetes"[Title/Abstract] OR T2DM[Title/Abstract] OR diabetes OR[Title/Abstract]) AND (Intervention[Title/Abstract] OR "controlled trial"[Title/Abstract] OR randomized[Title/Abstract] OR random[Title/Abstract] OR randomly[Title/Abstract] OR placebo[Title/Abstract] OR "clinical trial"[Title/Abstract] OR Trial[Title/Abstract] OR "randomized clinical trial"[Title/Abstract] OR RCT[Title/Abstract] OR trial[Title/Abstract] OR trials "Cross-Over Studies"[Title/Abstract] OR "Cross-Over"[Title/Abstract] OR "Cross-Over Study"[Title/Abstract] OR parallel[Title/Abstract] OR "parallel study"[Title/Abstract] OR "parallel trial"[Title/Abstract])
Scopus	(TITLE-ABS-KEY (probiotics OR probiotic OR synbiotics OR synbiotic OR lactobacillus OR bifidobacterium) AND TITLE-ABS-KEY (intervention OR "controlled trial" OR randomized OR random OR randomly OR placebo OR "clinical trial" OR trial OR "randomized clinical trial" OR rct OR trial OR trials "Cross-Over Studies" OR "Cross-Over" OR "Cross-Over Study" OR parallel OR "parallel study" OR "parallel trial"))
Web of science	TITLE: (Probiotics OR probiotic OR Synbiotics OR synbiotic OR Lactobacillus OR Bifidobacterium) AND TITLE: ("controlled trial" OR randomized OR random OR randomly OR placebo OR "clinical trial" OR Trial OR "randomized clinical trial" OR RCT OR trial OR trials "Cross-Over Studies" OR "Cross-Over" OR "Cross-Over Study" OR parallel OR "parallel study" OR "parallel trial")

Supplementary Table S2. Risk of bias assessment.

studies	Random sequence generation	Allocation concealment	Selective reporting	Other sources of bias	Blinding (participants and personnel)	Blinding (outcome assessment)	Incomplete outcome data	General risk of bias
Sotaniemi et al. 1995	L	L	H	H	L	U	L	Moderate
Vuksan et al. 2008	L	L	L	H	L	U	L	Low
Ma et al. 2008	L	L	H	H	L	U	L	Moderate
Reedes et al. 2011	L	L	L	H	L	U	L	Low
Yoon et al. 2012	L	L	L	H	L	U	L	Low
Hosseini et al. 2013	L	L	L	L	L	U	L	Low
Mucalo et al. 2013	L	L	H	H	L	U	L	Moderate
Park et al. 2014	L	L	H	H	L	U	L	Moderate
Bang et al. 2014	L	L	L	L	L	U	L	Low
Oh et al. 2014	L	L	L	H	L	U	L	Low
Mucalo et al. 2014	L	L	H	H	L	U	L	Moderate
Hosseini et al. 2016	L	L	H	H	L	U	L	Moderate
Hosseini et al. 2017	L	L	H	H	L	U	L	Moderate
Choi et al. 2018	L	L	L	H	L	U	L	Low
Vuksan et al. 2019	L	L	H	H	L	U	L	Moderate
Bessell et al. 2020	L	L	H	H	L	U	L	Moderate
Park et al. 2020	L	L	L	H	L	U	L	Low
Park et al. 2020	L	L	L	H	L	U	L	Low
Jovanovski et al. 2020	L	L	H	H	L	U	L	Moderate
Jovanovski et al. 2021	L	L	H	H	L	U	L	Moderate

*General Low Risk<2 high risk, General moderate risk=2 high risk, General high risk>2 high risk

Supplementary Table S3. Publication bias assessment.

Publication bias		
	Begg's Test	Egger's test
BW	1.000	0.799
BMI	0.754	0.578
WC	0.296	0.488
FPG	0.763	**0.035**
OGTT	0.721	0.258
A1c	0.921	0.681
Fasting Insulin	0.893	0.938
HOMA-IR	0.474	0.060
TG	0.193	0.409
TC	1.000	0.298
LDL-C	0.760	0.192
HDL-C	0.050	0.094
SBP	0.721	0.607
DBP	0.283	**0.050**
HR	0.296	0.403
CRP	1.000	0.219
IL6	0.133	**0.006**
TNF-α	0.260	0.558
Adiponectin	1.000	0.444
Leptin	0.296	0.261
ALT	0.072	0.182
AST	1.000	0.482
GGT	1.000	0.581

Abbreviations: body weight (BW); body mass index (BMI); waist circumference (WC); fasting plasma glucose (FPG); oral glucose tolerance test (OGTT); hemoglobin A1c (HbA1c); fasting insulin; homeostatic model assessment of insulin resistance (HOMA-IR); triglyceride (TG); total cholesterol (TC); low-density lipoprotein cholesterol (LDL-C); high-density lipoprotein cholesterol (HDL-C); systolic blood pressure (SBP); diastolic blood pressure (DBP); heart rate (HR); C-reactive protein (CRP); interlukin-6 (IL-6); tumor necrosis α (TNF-α); adiponectin; leptin; alanine aminotransferase (ALT); aspartate aminotransferase (AST); gamma-glutamyl transferase (GGT)
